# Targeting programmed cell death with natural products: a potential therapeutic strategy for diminished ovarian reserve and fertility preservation

**DOI:** 10.3389/fphar.2025.1546041

**Published:** 2025-05-29

**Authors:** Wenhan Ju, Shuai Zhao, Danping Li, Jinfu Zhang, Shan Xiang, Fang Lian

**Affiliations:** ^1^ Guanghua Hospital Affiliated to Shanghai University of Traditional Chinese Medicine, Shanghai, China; ^2^ The First Clinical Medical College, Shandong University of Traditional Chinese Medicine, Jinan, China; ^3^ Shenzhen Hospital Affiliated to Shanghai University of Traditional Chinese Medicine, Shenzhen, China; ^4^ Department of Reproduction and Genetics, Affiliated Hospital of Shandong University of Traditional Chinese Medicine, Jinan, China

**Keywords:** programmed cell death, natural products, diminished ovarian reserve, female fertility, IVF

## Abstract

The depletion of ovarian reserve is a major factor contributing to the decline in female fertility. It is characterized by a simultaneous reduction in the quantity and quality of oocytes and the follicular pools. The cyclic recruitment of primordial follicles and the preservation of oocyte quality involve complex and tightly regulated biological processes. Granulosa cells, which surround the oocytes, play a pivotal role in follicular development and the determination of follicular fate. Programmed cell death (PCD), a genetically regulated process of cell elimination, is a key factor in the regulation of ovarian reserve dynamics. Emerging evidence suggests that natural products derived from medicinal plants, dietary components, animals, and microorganisms may modulate PCD in granulosa cells through various molecular mechanisms and signaling pathways. These natural products have demonstrated preliminary effects in delaying ovarian aging and preserving ovarian reserve in preclinical models. This review discusses the roles and underlying mechanisms of various forms of PCD in diminished ovarian reserve, while summarizing the current findings on natural products that influence granulosa cells PCD to protect ovarian function. These insights may contribute to the future development of novel, targeted strategies aimed at preserving female reproductive potential.

## 1 Introduction

Ovarian reserve, defined by the quantity of primordial follicles and the quality of oocytes within the ovary, is used as an indicator of female fertility, with depletion recognized as a primary contributor to reduced fertility (Steiner et al., 2017). Under physiological conditions, the cyclic recruitment of primordial follicles and the gradual reduction in oocyte quality result in a year-by-year decrease in ovarian reserve, ultimately leading to its exhaustion after menopause. In pathological conditions, premature depletion of the ovarian reserve leads to early cessation of ovulation and estrogen deficiency. This depletion presents a significant challenge for women of advanced reproductive age seeking to conceive, highlighting the importance of fertility preservation as a major global health concern.

For female fertility preservation related to ovarian reserve, ovarian tissue cryopreservation is a well-established treatment. However, it is not suitable for women over 40 years of age and does not fundamentally improve fertility or extend ovarian endocrine function (Oktay et al., 2021). Stem cell transplants and stem cell-derived factors have been shown to hold potential for repairing or regenerating ovarian tissue (Buigues et al., 2021; Fàbregues et al., 2020; Liu H. et al., 2021). However, research in this field remains in its early stages, and further studies are required to establish their safety and efficacy. Additionally, some drugs, such as recombinant LH (Musters et al., 2012; Humaidan et al., 2017) and growth hormone (Bassiouny et al., 2016; Choe et al., 2018), have been reported to improve ovarian response and oocyte quality in women with diminished ovarian reserve (DOR). Despite these clinical reports, these drugs are not highly effective in improving fertility outcomes. Furthermore, they are costly and require prolonged use, imposing significant financial burdens on women with DOR who have fertility needs. Thus, it is important to elucidate the pathogenesis of DOR and the mechanisms underlying female fertility decline to develop new and effective treatments aimed at enhancing reproductive health.

The follicle comprises the oocytes and the surrounding granulosa cells, both of which are essential for female fertility and hormone production. The growth of granulosa cells and the maturation of oocytes within the follicle directly influence these processes. Oocytes secrete various factors into the follicular microenvironment to regulate granulosa cells function and maintain their development. Granulosa cells provide nutritional support to the oocytes, contribute to hormone synthesis, and play a role in follicular maturation and ovulation. Several mechanisms, including natural aging, alterations in the hypothalamic-pituitary-ovarian axis (Liu W. et al., 2021), oxidative stress (OS) (Wang et al., 2023a), DNA damage (Wu et al., 2024; Yao et al., 2023) and chronic inflammation (Wang D. et al., 2020), can affect granulosa cells function to varying degrees (Chen W. et al., 2023; Zhang T. et al., 2021). In addition, dysfunction in both oocytes and granulosa cells may disrupt the balance between primordial follicle dormancy and activation, resulting in premature depletion of the primordial follicle pools and impaired ovarian reproductive function. Moreover, granulosa cells dysfunction can directly reduce oocytes quality (Guo et al., 2023; Haraguchi et al., 2019).

Programmed cell death (PCD), a genetically controlled process of cell death (Tower, 2015), plays an important role in regulating follicular development, maintaining hormone homeostasis, and modulating ovarian reserve (Li H. et al., 2022; Wang et al., 2023a). The precise regulation of PCD is essential for maintaining granulosa cell function and preserving oocyte quality, which may help improve ovarian function. Studies have demonstrated that targeting the molecular mechanisms and signaling pathways involved in PCD can effectively restore granulosa cells functionality and enhance ovarian reserve. Furthermore, natural products derived from medicinal plants, dietary components, animals and microorganisms have shown the ability to modulate PCD through diverse targets, exhibiting significant potential for improving female reproductive function (Eslami et al., 2021; Sammad et al., 2024; Chen et al., 2022).

**FIGURE 1:**
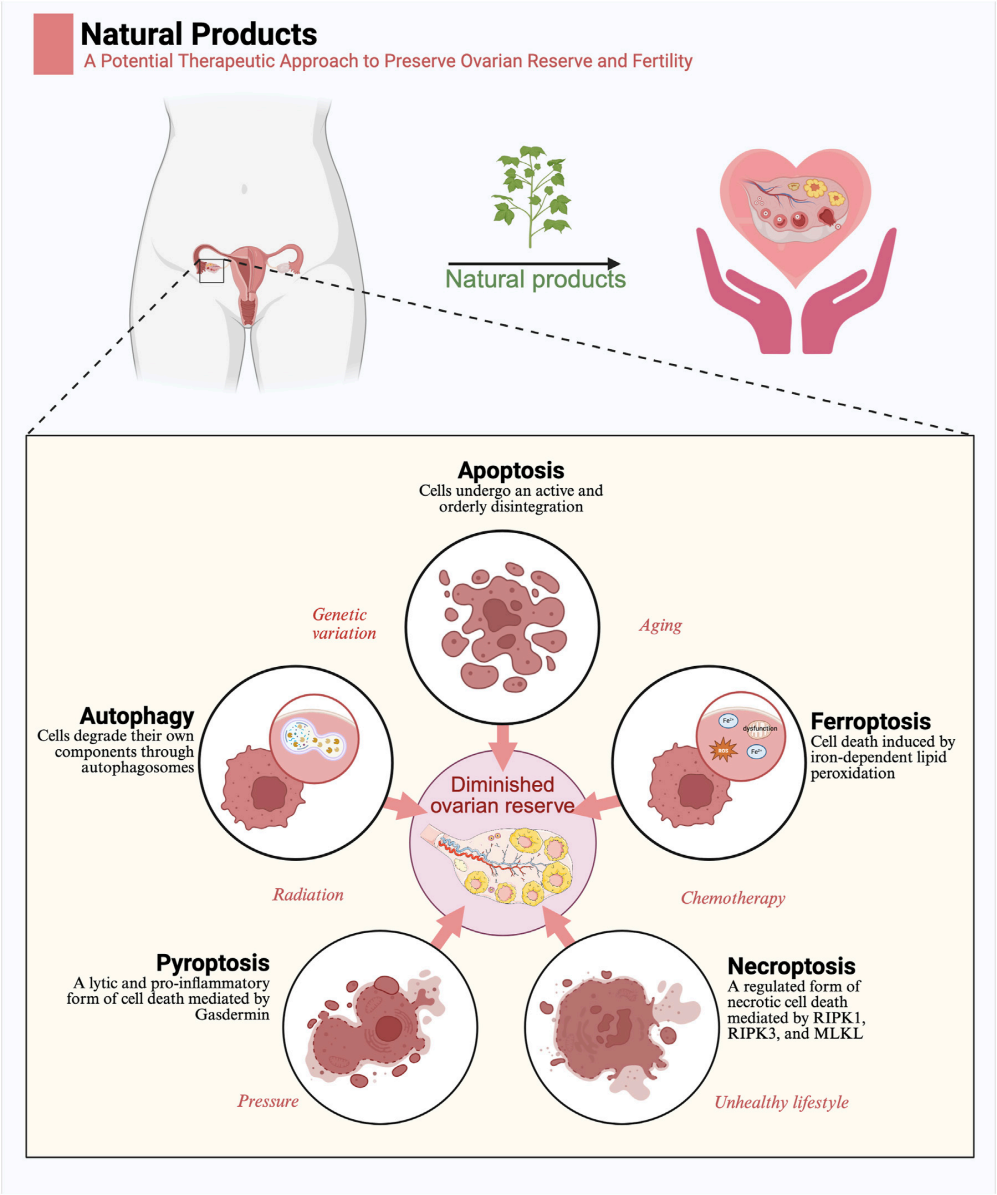
Natural products exert a protective function in diminished ovarian reserve by regulating programmed cell death: Five modes of programmed cell death, including apoptosis, autophagy, ferroptosis, pyroptosis, and necroptosis, are important pathogenetic mechanisms of diminished ovarian reserve. Factors such as genetic mutations, aging, radiation, chemotherapy, stress, and unhealthy lifestyle contribute to diminished ovarian reserve by influencing programmed cell death. Natural products may contribute to the preservation of ovarian reserve by regulating programmed cell death (Created in BioRender.com).

This article reviews the common forms of PCD involved in the pathogenesis of DOR, including apoptosis, autophagy, ferroptosis, pyroptosis, and necroptosis (Figure 1). The definitions, morphological features, biochemical characteristics, and major signaling pathways for these PCD types are summarized in Table 1. The involvement of granulosa cells PCD in DOR is discussed in detail, along with an overview of current findings on how natural products may modulate these processes to alleviate female reproductive dysfunction. Collectively, this review offers a comprehensive perspective that may inform the development of therapeutic approaches aimed at delaying reproductive aging in women.

**TABLE 1:** Modes and characteristics of programmed cell death.

Mode	Definition	Morphological features	Biochemical features	Main signalling pathways	References
Apoptosis	Cells undergo an active and orderly disintegration	Cell shrinkage and formation of apoptotic bodies	DNA fragmentation, activation of caspase enzymes	Extrisic pathway (death receptor pathway), intrisinc pathway (mitochondrial pathway), endoplasmic reticulum stress pathway	Galluzzi et al. (2012), Kulikov et al. (2012), Taneja et al. (2001)
Autophagy	Cells degrade their own components through autophagosomes	Organelle degradation and accumulation of autophagosomes	Autophagy-related protein (LC3, Beclin-1) activation	mTOR signalling pathway, AMPK signalling pathway	Yang and Klionsky (2010), Høyer-Hansen et al. (2007)
Ferroptosis	Cell death induced by iron-dependent lipid peroxidation	Lipid peroxidation and mitochondrial shrinkage with increased membrane density	Iron metabolism imbalance, GPX4 inactivation, lipid peroxide accumulation	Iron metabolic pathway, lipid metabolic pathway	Li et al. (2020a), Jiang et al. (2021)
Pyroptosis	A lytic and pro-inflammatory form of cell death mediated by Gasdermin	Cell swelling and plasma membrane rupture	Cleavage of the N-terminal domain of Gasdermin, caspase-1/11/4/5 activation, inflammatory factors IL-1β and IL-18 release	Caspase-1/11/4/5 signalling pathway	Kovacs and Miao (2017), Frank and Vince (2019)
Necroptosis	A regulated form of necrotic cell death mediated by RIPK1, RIPK3, and MLKL	Cellular swelling and plasma membrane rupture	RIPK1/RIPK3/MLKL activation, caspase-independence	RIPK1/RIPK3/MLKL pathway	Brault and Oberst (2017), Galluzzi et al. (2018)

## 2 Methods

We conducted a comprehensive search for existing studies on natural products for the treatment of diminished ovarian reserve and protection of female fertility by modulating PCD. PubMed, EMBASE and MEDLINE scientific databases were searched individually and/or in combination using the following keywords: (Apoptosis OR Autophagic cell death OR Necroptosis OR Pyroptosis OR Ferroptosis) AND (Natural products OR Plant metabolites OR Flavonoid OR Bioactivity OR Structure-activity relationship) AND (granulosa cells OR oocytes OR diminished ovarian reserve OR premature ovarian insufficiency OR premature ovarian failure OR ovarian aging OR poor ovarian response OR poor responders undergoing IVF OR poor response to ovarian stimulation). We included original scientific papers written in English and published up to 31 August 2024, that covered the aforementioned topics. Exclusion criteria were set as non-English literature, conference abstracts, book chapters, and non-relevant studies. Two independent researchers carried out an initial screening of the titles and abstracts of the literature. The literature that met the inclusion criteria advanced to full-text evaluation. Initially, a total of 897 potentially relevant publications were retrieved. Ultimately, after reviewing both the abstracts and full texts, 56 original research papers on the treatment of diminished ovarian reserve (DOR) with natural products were selected and included in the manuscript for review. In this manuscript, we meticulously traced the origin of each natural product mentioned in all the original research papers and precisely provided their authoritative taxonomic and family information (Supplementary Appendix 1–5).

In addition, we conducted a systematic search of randomized controlled trials (RCTs) to evaluate the clinical efficacy of natural products in patients with DOR or poor ovarian response (POR) undergoing assisted reproductive technology (ART). We searched databases including PubMed, EMBASE, and MEDLINE up to 31 August 2024, and only included studies published in English. The search strategy involved using the following keywords either individually or in combination: (Coenzyme Q10 OR Dehydroepiandrosterone OR Melatonin OR Resveratrol OR 2 - Oxoglutaric acid OR Allantoin OR Apigenin OR Berberine OR Capsaicin OR Chrysin OR Curculigoside OR Curcumin OR Daphnetin OR Diosgenin OR Epigallocatechin Gallate OR Eugenol OR Gallotannin OR Honokiol OR Icariin OR leonurine hydrochloride OR Nicotinamide mononucleotide OR Nobiletin OR Paeoniflorin OR Procyanidin OR Pterostilbene OR Puerarin OR Rutin OR Scutellarin OR Spermidine OR Sphingosine 1-phosphate OR α-Ketoglutarate) AND (Diminished ovarian reserve OR Poor ovarian response) AND (IVF OR ICSI OR ART OR Assisted reproductive technology) AND (Randomized controlled trial OR RCT). The inclusion criteria were as follows: the study type must be an RCT; the study subjects were women diagnosed with DOR or POR; the intervention was the supplementation of natural products as an adjuvant treatment for infertility; the control group received an IVF/ICSI protocol without the use of natural products or received a placebo; the outcome measures must include the live-birth rate or clinical pregnancy rate. The exclusion criteria were: non - RCT studies (such as observational studies, case reports, reviews, etc.); the study subjects were not clearly diagnosed with DOR/POR, or the study population was highly heterogeneous; the intervention involved non-natural products; the study data were incomplete, lacking the main outcome measures; there were duplicate publications of the study, and only the version with the most complete data was retained. The initial search yielded 162 relevant articles. After removing duplicates and screening the titles and abstracts, 56 articles remained for full-text evaluation. Finally, according to the inclusion and exclusion criteria, 8 RCTs were selected for the meta - analysis. In the meta - analysis, we used Review Manager 5.4 for statistical analysis. For binary variables (such as clinical pregnancy rate and live - birth rate), the odds ratio (OR) and 95% confidence interval (CI) were used for pooled analysis. Heterogeneity was assessed using the I^2^ statistic. An I^2^ value > 50% was considered to indicate significant heterogeneity. If the heterogeneity was large (I^2^ > 75%), a random - effects model was used; otherwise, a fixed - effects model was applied. Publication bias was evaluated using a funnel plot test, and sensitivity analysis was used to assess the robustness of the studies.

## 3 Natural products targeting apoptosis in cells with DOR

Apoptosis is a highly regulated form of PCD that plays a crucial role in maintaining tissue homeostasis and eliminating damaged or unnecessary cells. It is essential for various physiological processes, including embryonic development, immune regulation, and tissue remodeling. Dysregulation of apoptosis is closely linked to various pathological conditions, including cancer, neurodegenerative diseases, and reproductive disorders such as DOR. Understanding the molecular mechanisms of apoptosis provides valuable insights for developing targeted therapies to regulate cell survival and death in disease management.

### 3.1 Overview of apoptosis

**FIGURE 2:**
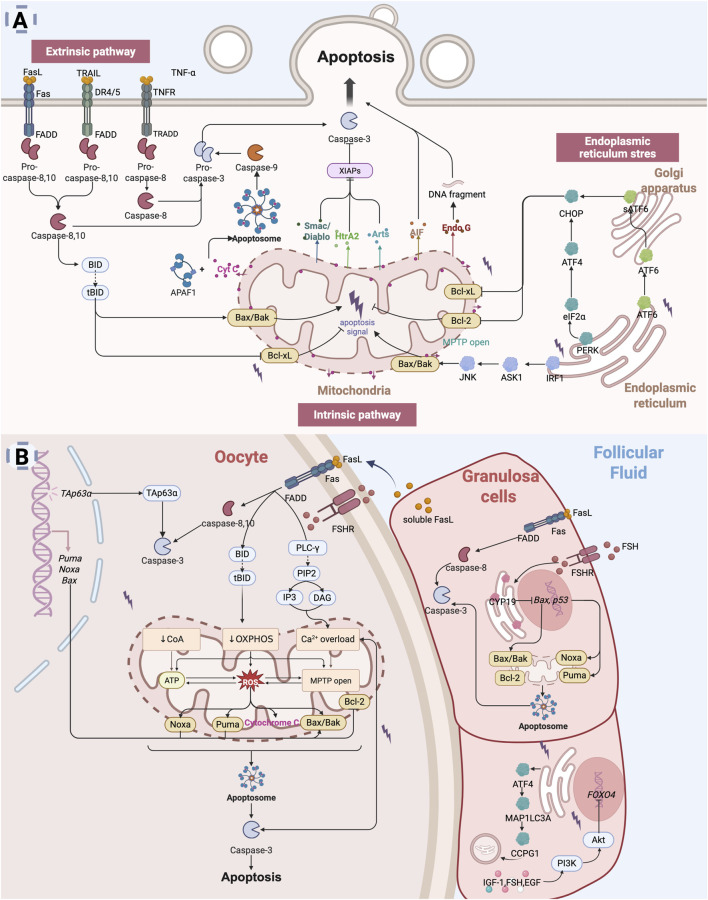
Apoptosis leads to diminished ovarian reserve: **(A)** Apoptosis is primarily mediated through three key pathways: the extrinsic pathway (death receptor pathway), the intrinsic pathway (mitochondrial pathway), and the endoplasmic reticulum stress (ER) pathway. The extrinsic pathway is initiated by signals from death receptors on the cell surface, which directly activate caspases, thereby initiating the apoptotic process (Galluzzi et al., 2012; Shakibaei et al., 2005). The intrinsic pathway is typically activated by intracellular stressors such as oxidative stress or DNA damage (Kulikov et al., 2012). This pathway involves mitochondrial alterations, including the release of Cyt C, which activates downstream caspases to induce apoptosis. The ER stress pathway is triggered by dysfunction in the endoplasmic reticulum, leading to increased expression of intracellular pro-apoptotic factors (Huang S. et al., 2019; Liang et al., 2020). **(B)** Mechanisms of Apoptosis in diminished ovarian reserve. In the context of DOR, oocytes are lost through apoptosis, a process regulated by specific gene expression (Huang et al., 2023). Both the extrinsic and intrinsic apoptotic pathways can be activated to initiate oocyte apoptosis (Zhu et al., 2016). Granulosa cells apoptosis, which disrupts the supportive microenvironment of the oocyte, further accelerates oocyte apoptosis (Zhu et al., 2016; Zhu et al., 2015). Multiple apoptotic pathways, including extrinsic, intrinsic, and ER stress-mediated signaling, contribute to granulosa cell apoptosis and ultimately accelerate ovarian reserve depletion (Inoue et al., 2007; Matsuda et al., 2008; Hsu et al., 1996; Ratts et al., 1995; Kugu et al., 1998; Perez et al., 1999; Li H. et al., 2023) (Created in BioRender.com).

Apoptosis is characterized by distinct cellular changes, including cell shrinkage, chromatin condensation, nuclear fragmentation, and the formation of apoptotic bodies. This process involves the activation of a cascade of caspase enzymes, particularly caspase-3, which executing apoptosis through the cleavage of key intracellular substrates. Apoptosis can be initiated via three principal pathways: the extrinsic (death receptor) pathway, the intrinsic (mitochondrial) pathway, and the endoplasmic reticulum (ER) stress pathway (Figure 2A).

The intrinsic pathway (mitochondrial pathway) involves two primary mechanisms of apoptosis: cytochrome c (Cyt C) release and the opening of the mitochondrial permeability transition pore (MPTP) (Kulikov et al., 2012; Taneja et al., 2001). Mitochondria are central to the regulation of apoptosis, with Cyt C functioning as a key pro-apoptotic molecule. Bax, a pro-apoptotic Bcl-2 family protein, resides in the cytoplasm and translocates to the mitochondria in response to apoptotic stimuli, where it facilitates the release of Cyt C (Cory and Adams, 2002). BH3-only proteins such as Bad support this process by neutralizing anti-apoptotic Bcl-2 members. Following apoptotic stimuli (e.g., DNA damage, growth factor deficiency), Bax/Bak forms oligomeric complexes that insert into the mitochondrial outer membrane, leading to changes in mitochondrial osmotic pressure. These oligomeric complexes cause a loss of transmembrane potential, prompting the release of Cyt C from the mitochondria into the cytoplasm. Then, Cyt C binds to apoptotic protease activating factor-1 (Apaf-1) to form the apoptosome that activates the caspase-9, which in turn activates caspase-3 and caspase-7 and induces apoptosis (Shalini et al., 2015). Simultaneously, mitochondria release Smac/Diablo, HtrA2, and Arts, which promote apoptosis by inhibiting the X-linked inhibitor of apoptosis protein (XIAP) (Tower, 2015). XIAP is a potent apoptosis inhibitor that directly inhibits caspases and regulates apoptosis through multiple mechanisms. Additionally, mitochondria release apoptosis-inducing factor (AIF) and endonuclease G (Endo G), which mediate caspase-independent apoptosis (Joza et al., 2001). The permeability transition pore (PTP), located between the inner and outer mitochondrial membranes, mediates the formation of the MPTP (Taneja et al., 2001). Molecules smaller than 1.5 kD can pass through the MPTP unselectively. When the mitochondrial matrix experiences high osmotic pressure, the MPTP channel opens, leading to apoptosis.

The extrinsic pathway refers to apoptosis mediated through death receptors located on the cell membrane surface (Galluzzi et al., 2012). Death receptors (DRs) are part of the tumor necrosis factor receptor (TNFR) superfamily, characterized by an extracellular cysteine-rich domain and an intracellular death domain (Ashkenazi and Dixit, 1998). Key death receptor-ligand pairs include Fas (APO-1/CD95)-FasL (CD95L), TNFR1 (DR1)-TNF, TRAILR1 (DR4)-TRAIL (APO-2L), TRAILR2 (DR5)-TRAIL (APO-2L), and DR3 (APO-3/TRAMP)-TL1A (Newton et al., 2024). Among these, the most extensively studied pathways involve Fas, TNFR1, and TRAIL-mediated signaling (Shakibaei et al., 2005). Upon binding with specific death ligands, death receptors receive extracellular death signals and initiate apoptotic signaling by recruiting specific adaptor proteins, which subsequently activate the caspase cascade. FasL binding induces the trimerization of Fas, which recruits the adaptor protein FADD (Fas-associated death domain) and the initiator caspase-8 or caspase-10, which are oligomerized and activated through autocatalysis. Activated caspase-8/10 induces apoptosis through two parallel pathways (Legembre et al., 2002): it either directly cleaves and activates caspase-3 or cleaves the pro-apoptotic Bcl-2 family protein Bid to generate truncated Bid (tBid). tBid translocates to the mitochondria, triggering cytochrome c release and subsequent activation of caspase-9 and caspase-3. Similarly, TRAIL binding to DR4 or DR5 recruits FADD and activates caspase-8/10, triggering downstream apoptotic events (Gonzalvez and Ashkenazi, 2010). The binding of TNF-α to TNFR1 initiates apoptosis via the adaptor protein TRADD, which recruits FADD and subsequently activates caspase-8 to promote the apoptotic cascade (Shakibaei et al., 2005).

The ER stress response is a cellular mechanism triggered by ER dysfunction, such as protein misfolding or calcium ion imbalance. The accumulation of misfolded proteins leads to severe ER stress (ERS), which activates the unfolded protein response (UPR) (Oakes and Papa, 2015). Under prolonged or severe UPR conditions, three transmembrane ER proteins—PERK, IRE1 and ATF6—become activated, promoting the upregulation of pro-apoptotic factors, including CHOP (Chen et al., 2023b). IRE1 interacts with the adaptor protein TRAF2, leading to the activation of c-Jun N-terminal kinase (JNK) (Huang S. et al., 2019). Activated JNK facilitates the translocation of pro-apoptotic proteins Bax and Bak to the mitochondria and simultaneously inhibits the anti-apoptotic activity of Bcl-xL, thereby indirectly triggering the mitochondrial apoptotic pathway. PERK promotes the phosphorylation of eukaryotic initiation factor-2α (eIF2α), which subsequently induces the expression of ATF4, a key mediator of ER stress-related apoptosis (Fan and Jordan, 2022). ATF6, upon activation, translocates to the Golgi apparatus, where it is cleaved into its active form, sATF6. The sATF6 protein then translocates to the nucleus and mediates apoptosis by promoting CHOP expression (Liang et al., 2020).

### 3.2 Apoptosis and DOR

Apoptosis is an fundamental process in the ovary, regulating its development and function throughout the female life cycle. Oocytes in the human ovary originate from embryonic primordial germ cells, which undergo limited mitosis. The follicular reserve established at birth is finite, and a progressive decline in follicle numbers is a hallmark of diminished reproductive capacity (Hansen et al., 2008; Faddy et al., 1992). Primordial follicles (PMFs), which constitute the foundation of the ovarian reserve, are continuously recruited into the pool of growing follicles within the ovary. Apoptosis plays a pivotal role in follicular atresia and follicular depletion, with follicular activation and growth closely associated with cell proliferation and resistance to apoptosis (Krysko et al., 2008). The survival or apoptosis of PMFs is determined by the balance between the expression of pro-apoptotic and anti-apoptotic factors (Meng et al., 2018). It is well-established that granulosa cells apoptosis is the primary driver of atresia in growing follicles, while oocyes apoptosis contributes to primordial follicular atresia. Oocytes apoptosis results directly in germ cell loss, whereas granulosa cells apoptosis leads to follicular atresia. Excessive apoptosis accelerates the decline in ovarian reserve, a phenomenon particularly evident in conditions such as DOR, premature ovarian insufficiency (POI), and premature ovarian failure (POF).

When oocytes experience DNA damage due to chemotherapy, radiotherapy, environmental toxins, or natural aging, members of the p53 family, particularly the TAp63α isoform, are activated. This activation involves phosphorylation and nuclear accumulation of TAp63α in oocytes, which subsequently triggers the expression of pro-apoptotic Bcl-2 family members, including PUMA, NOXA, and Bax, initiating the apoptotic pathway (Suh et al., 2006; Gonfloni et al., 2009; Kerr et al., 2012; Huang et al., 2023). With ovarian aging, the fully glycosylated follicle-stimulating hormone (FSH) variant 24 (FSH24) increases, exhibiting reduced affinity for the FSH receptor (FSHR) (Agwuegbo et al., 2021). Decreased FSH activity leads to reduced activation of G protein-coupled receptor (GPCR) and adenylyl cyclase (AC) signaling, resulting in insufficient cAMP production, which makes oocytes more susceptible to oxidative stress-induced apoptosis. Increased intracellular Ca^2+^ levels, along with reduced cAMP, elevate mitochondrial reactive oxygen species (ROS) production (Jin et al., 2022), which upregulates the Bax/Bcl-2 ratio on the mitochondrial membrane, triggering mitochondrial transmembrane potential loss and Cyt C release. Released Cyt C activates the caspase cascade, particularly caspase-3, leading to apoptosis (Zhu et al., 2016). Activated caspase-3 further promotes Ca^2+^ release, creating a vicious cycle that amplifies caspase-3 activation and ultimately results in oocyte apoptosis.

Granulosa cells survival is crucial for oocytes viability, and their apoptosis disrupts the follicular microenvironment, accelerating follicular atresia (Yao et al., 2023; Zhao et al., 2024). Healthy growing follicles exhibit resistance to granulosa cells apoptosis, whereas in early atretic follicles, apoptosis begins in the inner granulosa cells layer and progressively affects most granulosa cells, leading to follicular atresia (Jiang et al., 2003; Inoue et al., 2011; Tilly et al., 1991). Aging leads to a loss of CYP19, a key enzyme in estrogen synthesis, resulting in the upregulation of pro-apoptotic genes such as p53 and Bax, thereby promoting granulosa cells apoptosis (Britt et al., 2000; Toda et al., 2001). Senescent oocyte-surrounding cumulus granulosa cells release soluble FasL (sFasL), which binds to Fas receptors on oocytes (Matsuda-Minehata et al., 2006; Hakuno et al., 1996), triggering ER Ca^2+^ release via the phospholipase C-γ (PLC-γ) pathway and Cyt C activation (Zhu et al., 2016; Matsuda-Minehata et al., 2006; Hakuno et al., 1996; Zhu et al., 2015), further driving oocytes apoptosis. FADD and caspase-8 also contribute to granulosa cells apoptosis (Inoue et al., 2007; Matsuda et al., 2008). Bcl-2-deficient mice exhibit a reduced number of oocytes and primordial follicles (Hsu et al., 1996; Ratts et al., 1995), while Bax-deficient mice display an excessive number of follicles (Kugu et al., 1998; Perez et al., 1999), further confirming the importance of apoptotic signaling balance in maintaining ovarian reserve. Furthermore, the ATF4/MAP1LC3A/CCPG1 pathway may induce apoptosis via ER autophagy activation (Li H. et al., 2023).

Regulatory factors in the ovarian microenvironment, such as estradiol (E_2_), insulin-like growth factor (IGF) (Glister et al., 2001; Monniaux and Pisselet, 1992), FSH, and epidermal growth factor (EGF) (Tilly et al., 1992; Park et al., 2005; Cunningham et al., 2003), are recognized as pro-survival factors for granulosa cells. However, levels of E_2_, IGF, and EGF are significantly reduced in DOR (Stadtmauer et al., 1998; Wang C. et al., 2023; Huang W. et al., 2019; Liu et al., 2017) The loss of these pro-survival factors disrupts the balance between anti-apoptotic and pro-apoptotic signaling, increasing granulosa cells susceptibility to apoptosis.

In general, apoptosis of granulosa cells reduces the availability of hormones and survival factors that are essential for oocytes growth and maturation, impairing oocytes meiotic and developmental competence. This, in turn, increased vulnerability of oocytes to apoptotic stimuli, exacerbating follicular atresia and contributes to the decline of ovarian reserve function (Figure 2B). Suppressing apoptosis-related pathways and enhancing granulosa cell viability and resistance to apoptosis represent promising therapeutic strategies to preserve follicular numbers and prolong fertility. Targeting apoptosis-related mechanisms may effectively delay ovarian reserve decline, offering a viable approach to fertility preservation and reproductive health management.

### 3.3 Regulation of apoptosis by natural products

As previously discussed, apoptosis in oocytes and granulosa cells can be triggered by various factors, including chemotherapy, radiotherapy, environmental toxins, and natural aging. Apoptosis plays a significant role in DOR. Consequently, preventing follicular atresia by inhibiting apoptosis in oocytes and granulosa cells represents a promising strategy for preserving ovarian reserve. Among the numerous candidate therapeutic resources, natural products derived from plants and dietary sources have gained significant attention due to their long history of use and relatively high safety profile. These plant metabolites are increasingly recognized as important adjunctive interventions for managing DOR, offering potential benefits in supporting ovarian function and improving reproductive outcomes.

Currently, numerous natural products have been investigated as potential modulators of DOR, primarily in preclinical models (Supplementary Appendix 1). Allantoin has been reported to reduces granulosa cells apoptosis in cyclophosphamide (CTX)-injured rats by downregulating the Bax/Bcl-2 ratio (Wang et al., 2023c). α-Ketoglutarate may contribute to the restoration of ovarian reserve and improve pregnancy rates in a CTX-induced POI rat model, potentially by inhibiting Caspase-3 activity (Li T. et al., 2023). Apigenin, curcumin, and resveratrol appear to attenuate oxidative stress- or chemotherapy-induced granulosa cells apoptosis, possibly by modulating apoptotic signaling pathways including the Bax/Bcl-2 ratio or inhibiting the caspase cascade (Fabová et al., 2023; Talebi et al., 2020; Saman et al., 2023; Li X. et al., 2022; Mantawy et al., 2019; Mobasher et al., 2022; Liang et al., 2024; Yan et al., 2018; Duan et al., 2024; Tsui et al., 2017; Barberino et al., 2022; Sirotkin et al., 2021; Ibrahim et al., 2021; Liu W. et al., 2022; Liu X. et al., 2018; Zhang et al., 2016; Faghani et al., 2022; Shang et al., 2023; Xin et al., 2023; Li F. et al., 2024; Ding et al., 2024; Xu G. et al., 2023; Bai et al., 2024; Li Y. et al., 2020; Xi et al., 2024; Wu et al., 2018; Chen et al., 2021; Zhang et al., 2024; Yi et al., 2021; Wu et al., 2021; Zhao et al., 2022; Sirotkin et al., 2020; Zhou et al., 2023). However, these compounds are widely distributed in nature and often suffer from poor bioavailability, raising questions about the translational relevance of their observed effects *in vitro*. Whether these effects translate into clinically meaningful outcomes remains uncertain. Additionally, honokiol, *Cuscuta chinensis* Lam. extract, *Panax ginseng* C.A.Mey. extract, and peptides have demonstrated protective effects on ovarian function in animal studies, potentially via activation of the Nrf2/HO-1 pathway (Liang et al., 2024; Shang et al., 2023; Xin et al., 2023; Zhao et al., 2022). Combinatorial strategies, such as resveratrol with *Citrus × limon (L.) Osbeck* peel extract, have been proposed to enhance efficacy through synergistic inhibition of apoptotic signaling pathways like iNOS/caspase-3 (Mobasher et al., 2022). Nonetheless, mechanistic evidence for such synergy is limited and primarily inferential. Melatonin and puerarin have also been associated with reductions in ovarian cell apoptosis, possibly through modulation of the eIF2α/ATF4 and Wnt/β-catenin signaling pathway respectively (Ding et al., 2024; Wu et al., 2018).

Notably, most studies on natural products have been conducted in experimental animals, with only one study focusing on primary granulosa cells from human follicular fluid (Liang et al., 2023). Mechanistic interpretations in these studies typically rely on indirect evidence, such as changes in protein markers (e.g., Bax/Bcl-2 ratio, caspase activity), while direct causal validation—through gene silencing, overexpression, or target binding assays—is rare (Xu G. et al., 2023). Moreover, none of the included studies conducted systematic analyses of small molecule–protein interactions, and the pharmacokinetic relevance of the doses used *in vitro* versus *in vivo* studies remains largely unaddressed. Among the ten *in vivo* studies, although various dosages were tested, neither a clear dose-response relationship nor long-term toxicity assessments (e.g., liver and kidney function indicators) were reported (Wang et al., 2023c; Li X. et al., 2022; Duan et al., 2024; Barberino et al., 2022; Faghani et al., 2022; Shang et al., 2023; Li Y. et al., 2020; Chen et al., 2021; Wu et al., 2021; Zhao et al., 2022). In the five *in vitro* studies, although a decrease in the apoptosis rate of granulosa cells was observed at specific doses, it was not confirmed whether this effect could translate into an improvement in oocyte quality *in vivo* (Fabová et al., 2023; Tsui et al., 2017; Sirotkin et al., 2021; Xu G. et al., 2023; Sirotkin et al., 2020). Taken together, while these natural products exhibit potential biological activity relevant to DOR, current evidence remains preliminary and largely exploratory. Further studies involving robust mechanistic validation, pharmacokinetic profiling, and clinical translation are essential to determine whether these compounds possess genuine therapeutic potential or if their effects are limited to experimental settings. Therefore, their biological relevance *in vivo* remains uncertain and requires further investigation.

## 4 Natural products targeting autophagy in cells with reduced ovarian reserve function

Apoptosis is not the sole mechanism underlying the development and progression of DOR. In recent years, studies have shown that autophagy also plays a crucial role in maintaining ovarian function and regulating the progression of DOR. By degrading damaged organelles and clearing intracellular harmful substances, autophagy helps maintain cellular homeostasis and can influence the apoptotic process to some extent. Therefore, modulating autophagy may offer a novel intervention strategy for improving DOR.

### 4.1 Overview of autophagy

Autophagy is a conserved intracellular degradation process that maintains cellular homeostasis by degrading and recycling damaged organelles and misfolded or excess proteins via autophagosomes (Yang and Klionsky, 2020). This process is activated under conditions such as nutrient deprivation, OS, or other cellular stressors. By breaking down intracellular components, autophagy sustains bioenergetic balance and promotes cell survival until favorable conditions are restored (Dikic and Elazar, 2018). Autophagy is categorized into three types based on its mechanism and mode of degradation: macroautophagy, microautophagy, and chaperone-mediated autophagy (CMA). Among these, macroautophagy is the most extensively studied, particularly in nutrient deficiencies and cellular stress responses. Herein, we focus on macroautophagy, referred to simply as “autophagy,” due to its central role in regulating cellular responses to stress and its relevance to ovarian reserve function.

Autophagy is a highly conserved catabolic process involving four key steps: initiation, autophagosome formation and maturation, fusion with lysosomes, and degradation of autophagic cargo. The mechanistic target of rapamycin (mTOR) functions as a major negative regulator of autophagy. In nutrient-rich conditions, mTOR remains active, suppressing autophagy initiation, while under stress conditions such as energy deficiency or DNA damage, the activation of AMP-activated protein kinase (AMPK), p53 or inhibition of pathways like PI3K/Akt and ERK reduces mTOR activity, which then releases the inhibition on autophagy and facilitates its initiation (Yang and Klionsky, 2010). Additionally, ER stress can induce AMPK activation via increased cytosolic Ca^2+^ levels, providing an alternative route for autophagy initiation (Høyer-Hansen et al., 2007). Once activated, AMPK stimulates the ULK1 complex, including ULK1, Atg13, Atg101, and FIP200, marking autophagy initiation. The activated ULK1 complex subsequently activates downstream signaling molecules. Among these, the class III phosphatidylinositol 3-kinase (PI3K-III) complex, comprising Vps34, Beclin-1, Vps15 and Atg14, is responsible for generating phosphatidylinositol 3-phosphate (PI3P) (Klionsky et al., 2021). PI3P facilitates the recruitment of Atg proteins to the developing autophagic membrane, promoting vesicle formation and expansion. Two ubiquitin-like conjugation systems are crucial for vesicle elongation (Mizushima and Komatsu, 2011). In the first, Atg7 and Atg10 mediate the conjugation of Atg12 to Atg5. The Atg12-Atg5 conjugate then binds to Atg16, forming a complex that interacts with the second conjugation system. In this system, Atg4, Atg7, and Atg3 mediate the conjugation of soluble LC3 (microtubule-associated protein 1 light chain 3) to phosphatidylethanolamine (PE), producing LC3-II. LC3-II is a key marker protein of autophagosomes, enabling the encapsulation of cytoplasmic materials targeted for degradation (Mizushima and Komatsu, 2011). After autophagosome formation, SNARE proteins such as VAMP8, STX17, and SNAP29 facilitate the fusion of autophagosomes with lysosomal membranes to form autolysosomes (Itakura et al., 2012). The small GTPase Rab7 regulates the transport of autophagosomes and promotes their interaction with lysosomes (Wang et al., 2016). In autolysosomes, lysosomal hydrolases degrade the contents of autophagosomes, including damaged organelles, misfolded proteins, and other cellular debris. The resulting degradation products, such as amino acids and fatty acids, are recycled to fulfill the metabolic and synthetic demands of the cell, thus maintaining cellular homeostasis.

**FIGURE 3:**
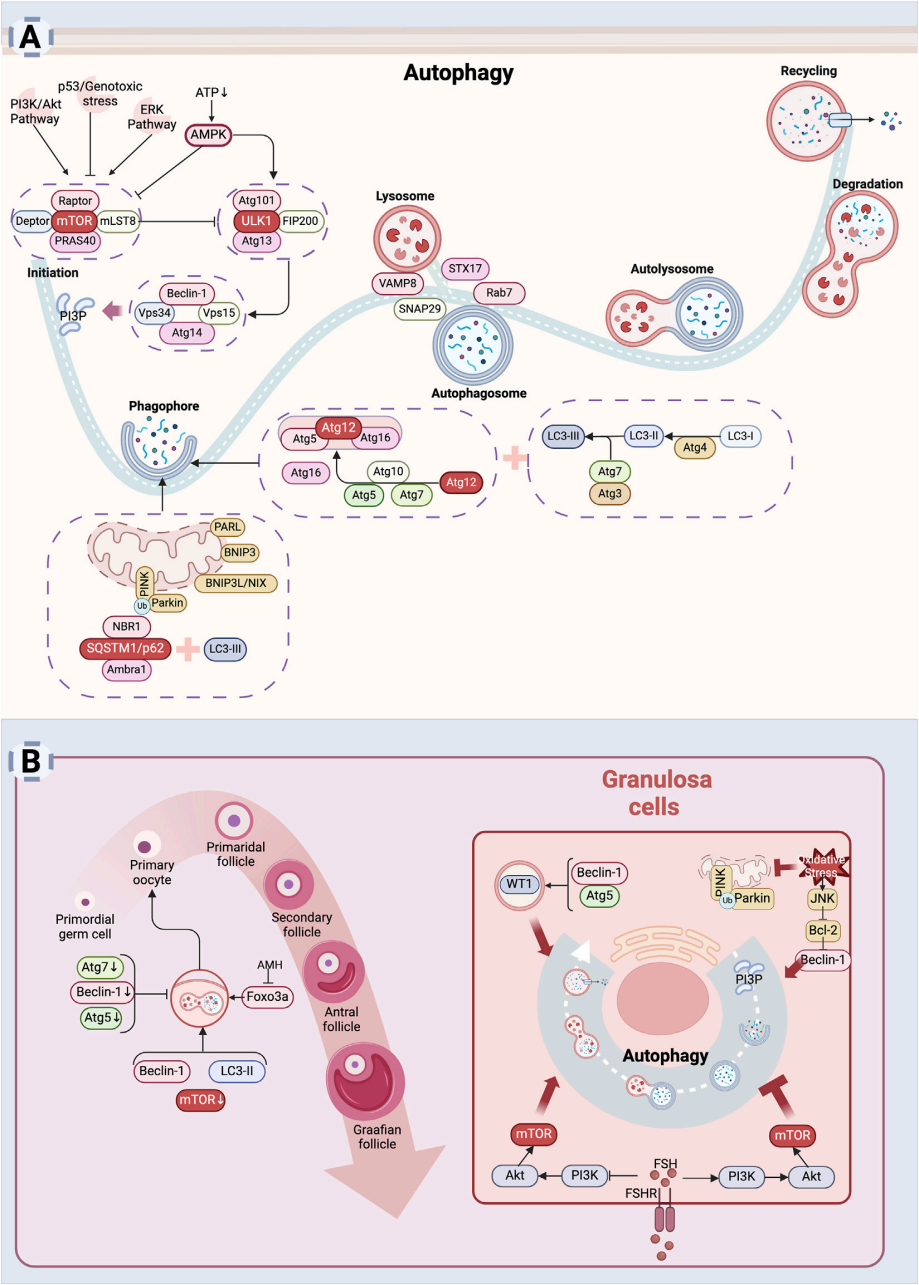
Autophagy leads to diminished ovarian reserve: **(A)** Mechanism of autophagy. Autophagy is a process by which cells degrade themselves. Signals such as AMPK, PI3K/Akt, ERK, p53, etc. influence autophagy by regulating mTOR activity (Yang and Klionsky, 2010). After autophagy is initiated, autophagosomes are assembled inside the cell to wrap damaged organelles or aggregated proteins inside the cell (Mizushima and Komatsu, 2011). Subsequently, autophagosomes fuse with lysosomes to form autophagic lysosomes, and the internal material is degraded by lysosomal enzymes (Itakura et al., 2012). Finally, the degradation products are released back into the cytoplasm for re-synthesis of substances needed by the cell or to provide energy. **(B)** Mechanism of cellular autophagy in ovarian reserve hypoplasia. Autophagy is involved in the maintenance of the primordial follicle number (Cao et al., 2017; Sonigo et al., 2019). Autophagy helps granulosa cells adapt to adverse external stimuli and promotes granulosa cells survival (Shao et al., 2022). Excessive autophagy also induces granulosa cells death (Cao et al., 2018) (Created in BioRender.com).

Mitochondrial autophagy, also known as mitophagy, is a selective process that removes damaged mitochondria (Lu et al., 2023). Under normal conditions, PTEN-induced putative kinase 1 (PINK1) is continuously degraded by presenilin-associated rhomboid-like protein (PARL). When mitochondrial damage occurs, PINK1 accumulates on the mitochondrial outer membrane and recruits the E3 ubiquitin ligase Parkin (Picca et al., 2023). Parkin ubiquitinates outer mitochondrial membrane proteins, including voltage-dependent anion channel 1 (VDAC1) and mitofusins (Mfn1 and Mfn2), thereby initiating mitophagy. Following ubiquitination, receptor proteins such as sequestosome-1 (SQSTM1 or p62) bind to LC3-II via their LC3-interacting region (LIR) (Wang S. et al., 2023). Additionally, BCL2/adenovirus E1B 19 kDa protein-interacting protein 3 (BNIP3), BNIP3-like protein (BNIP3L or NIX), and FUN14 domain-containing protein 1 (FUNDC1), which also contain LIR motifs, promote the formation of autophagosomes (Wang S. et al., 2023). These mechanisms are detailed in Figure 3A.

### 4.2 Autophagy and DOR function

Autophagy is necessary for maintaining oocyte development, follicular growth and differentiation, follicular atresia, and the reproductive cycle (Zhou et al., 2019; Choi et al., 2010). Impaired autophagy in the follicle can decrease oocyte quality, contributing to infertility in women (Li et al., 2019). Evidence has shown a relationship between autophagy and oocyte development and survival. Atg7 is expressed at all follicular stages, from primordial follicles (PMF) to antral follicles, without significant variation, while Beclin-1 shows the highest relative expression in PMFs (Cao et al., 2017; Song et al., 2015). Deficiency in key autophagy molecules, such as Atg7 and Beclin-1, may contribute to DOR (Cao et al., 2017; Gawriluk et al., 2011; Texada et al., 2019), as confirmed in animal studies. Atg7 knockout leads to a significant decrease in oocyte numbers, ultimately causing infertility in mice (Song et al., 2015). Additionally, Atg5 knockout affects steroid hormone synthesis due to cholesterol deficiency caused by impaired autophagy (Texada et al., 2019). Under stress conditions, autophagy is activated to maintain PMF numbers. Serum starvation promotes PMF formation in neonatal mouse ovaries by activating autophagy and inhibiting apoptosis *in vitro* (Rodrigues et al., 2009). AMH contributes to preserving PMF numbers by inhibiting Foxo3a phosphorylation-induced autophagy in the ovaries (Sonigo et al., 2019). Furthermore, the autophagy enhancer rapamycin inhibits the mTOR pathway, upregulates the expression of LC3-Ⅱ and Beclin-1, triggers autophagy, facilitates the aggregation of PMF, and improves the survival rate of oocytes (Sun et al., 2018).

Granulosa cells play a key role in providing nutritional support, hormone synthesis and regulation, follicle maturation, and ovulation, making them crucial in determining follicular fate. Autophagy mediates granulosa cell differentiation by degrading the transcription factor WT1, with Atg5 and Beclin-1 being essential genes in this process (Shao et al., 2022). Rab7 regulates mitophagy, which influences oocyte quality (Jin et al., 2022). During follicular atresia, autophagy and apoptosis often occur simultaneously in granulosa cells, promoting follicular degradation and ovarian remodeling (Tanner et al., 2011). Granulosa cells autophagy is predominantly observed in medium-sized follicles (2–6 mm in diameter), while apoptosis is more common in large follicles (>6 mm in diameter) (Zheng et al., 2019). With aging, accumulated oxidative damage leads to a decline in granulosa cell autophagy. Plasma levels of advanced oxidation protein products (AOPP) are elevated in women with POI, inhibiting granulosa cells autophagic flux and lysosomal biosynthesis (Zhou X. Y. et al., 2024). Therefore, maintaining a certain level of autophagy in granulosa cells is crucial for female fertility regulation.

Autophagy is influenced by various factors. Smoking induces autophagy, reducing granulosa cell numbers (Gannon et al., 2012). Excess oxidized low-density lipoprotein (oxLDL) in the follicular fluid of obese women increases ROS levels via NADPH oxidase, further enhancing granulosa cell autophagy (Lai et al., 2018). Melatonin blocks JNK-mediated autophagy, protecting granulosa cells from oxidative stress damage (Cao et al., 2018), while also promoting mitophagy to protect mitochondrial function (Xu G. et al., 2023). FSH exhibits dual regulatory effects on granulosa cells autophagy. During follicular development, FSH activates the PI3K/AKT/mTOR pathway to inhibit autophagy, promoting granulosa cells proliferation and supporting follicular development (Texada et al., 2019; Choi et al., 2014; Shen et al., 2017; Shen et al., 2016). However, in atretic follicles, FSH may activate autophagy via different signaling pathways, leading to granulosa cells degradation and triggering follicular atresia (Choi et al., 2010; Choi et al., 2014). This suggests that autophagy plays a dual role in different stages of follicular development—promoting survival during follicle formation while facilitating atresia under appropriate conditions to regulate ovarian reserve (Duerrschmidt et al., 2006).

Overall, the role of autophagy in establishing the follicular pool and promoting follicular survival highlights its potential as a novel therapeutic target for addressing DOR in vulnerable populations, such as those exposed to chemotherapy, environmental toxins, or genetic disorders (Figure 3B). However, excessive activation of autophagy may result in adverse effects, as the process also contributes to follicular atresia and granulosa cells death. It is important to note that most current insights into autophagy are based on the detection of autophagy-related factors, and accurately quantifying autophagic flux remains a significant challenge. Therefore, the dual role of autophagy in ovarian reserve requires further investigation. The application of autophagy-related molecules as therapeutic targets to counteract DOR is still in its early stages and warrants additional research to clarify both their therapeutic potential and associated risks.

### 4.3 Regulation of autophagy by natural products

Autophagy plays a pivotal role in female follicular processes, including the establishment of the follicular pool, follicular development, and follicular apoptosis, reflecting a complex and dualistic nature. This dualistic function suggests that modulation of autophagy by natural products could potentially influence ovarian reserve through either activation or inhibition pathways. Current findings related to 13 natural products are summarized in Supplementary Appendix 2.

Eight natural products have been reported to enhance autophagic activity in granulosa cells, which may help mitigate oxidative stress, aging, and chemotherapy-induced damage. ROS is considered a major physiological inducer of cellular senescence (Sun D. et al., 2023), with hydrogen peroxide (H_2_O_2_) widely used to establish OS models due to its stability and prolonged activity (Zhou J. et al., 2022). In H_2_O_2_-induced oxidative stress models, curcumin has been shown to promote autophagy, potentially via inhibition of the AMPK/mTOR pathway in granulosa cells (Duan et al., 2024). Paeoniflorin and melatonin may stimulate mitophagy by upregulating PINK1 and Parkin, enhancing lysosomal fusion and mitochondrial turnover (Xu G. et al., 2023; Xi et al., 2024). Similarly, melatonin, procyanidin B2, quercetin, and spermidine have been associated with increased expression of LC3-II and Beclin-1, suggesting enhanced autophagic flux and improved granulosa cells viability under oxidative stress conditions (Zhang et al., 2016; Li P. et al., 2024; Cai et al., 2024). In aging and chemotherapy models, natural products also appear to modulate autophagic dysfunction. D-galactose is a classical aging model (Wang Y. et al., 2022), and aging in mammals is often accompanied by decreased autophagic flux, which also occurs in ovarian tissues.

Spermidine, a natural polyamine known to decrease with age in various model organisms and humans (Eisenberg et al., 2009; Gupta et al., 2013),has been reported to promote autophagy and reduce the number of atretic follicles in aged mice (Madeo et al., 2018; Madeo et al., 2019; Jiang et al., 2023; Niu et al., 2023). Similarly, nicotinamide mononucleotide (NMN), a key intermediate in cellular energy metabolism, also decreases with age (Yoshino et al., 2018; Imai and Guarente, 2014). NMN supplementation has been linked to enhanced mitophagy in ovarian granulosa cells of naturally aging mice (Huang et al., 2022). Nobiletin, a common natural flavonoid, has been found to activate mitophagy via AMPK and SIRT1 pathways, potentially alleviating D-galactose-induced mitochondrial damage in granulosa cells and delaying age-related apoptosis (Bai et al., 2024). Additionally, resveratrol and its modified form, resveratrol-βcd, may also enhance autophagy by inhibiting mTOR signaling, with preliminary evidence of improved ovarian reserve in POI mouse models (Park et al., 2016; Hu et al., 2024).

Conversely, five natural products have demonstrated inhibitory effects on autophagy in ovarian cells, a mechanism that may also offer protective benefits under certain pathological conditions. Allantoin has been associated with mitochondrial membrane potential (MMP), and suppression of mitophagy and ROS levels, contributing to improved ovarian function in CTX-induced POI models (Wang et al., 2023c). Curculigoside has been reported to downregulate Beclin-1 expression and the LC3-II/I ratio, potentially reducing autophagy-related damage (Meng et al., 2024). Dehydroepiandrosterone (DHEA) may inhibit mitophagy via suppression of AMPK/SIRT1 pathway (Ma et al., 2024). Procyanidins appears to modulate autophagy in aging or oxidative stress conditions by regulating FoxO1 suppression or PI3K-Akt pathway activation, potentially promoting ROS clearance (Zhou S. et al., 2022). Melatonin has also been implicated in the suppression of autophagy under various stress conditions, including nicotine exposure, serum starvation, excessive autophagy, and unpredictable stress, possibly through modulation of miR-15a-5p, PI3K/Akt/mTOR, and eIF2α/ATF4 pathways (Wang et al., 2018; Wu et al., 2022; Liu Y. et al., 2022; Xie et al., 2021; Ding et al., 2024).

Overall, autophagy, as a crucial degradative pathway for maintaining cellular homeostasis, with both insufficient and excessive activity contributing to cellular dysfunction. Natural products appear to exert modulatory effects on this system, either by activating protective autophagy to clear damaged organelles and reduce oxidative load, or by inhibiting excessive autophagy to prevent unnecessary cellular degradation. However, current evidence is primarily derived from *in vitro* models or animal studies, and often relies on indirect indicators such as protein expression changes (e.g., LC3-II, Beclin-1). Although pharmacological inhibitors like 3-MA have been used to infer involvement of autophagy (Duan et al., 2024; Cai et al., 2024), most studies lack gene-level validation (e.g., siRNA knockdown, gene overexpression) to establish causality. Moreover, while a few studies have conducted preliminary pharmacokinetic analyses (Hu et al., 2024), systematic evaluation of drug metabolism, long-term toxicity, and *in vivo* efficacy remains limited. These gaps underscore the need for more rigorous approaches, including gene-editing technologies, multi-omics analysis, and *in vitro* and *in vivo* models to elucidate the molecular mechanisms by which natural products may regulate autophagy and to assess their potential translational relevance in ovarian function preservation.

## 5 Natural products targeting ferroptosis in cells with DOR

Besides autophagy, ferroptosis, a newly identified form of PCD, also plays a crucial role in the occurrence and progression of DOR. Increasing evidence suggests that OS, mitochondrial dysfunction, and the inflammatory microenvironment can induce ferroptosis in ovarian granulosa cells. Meanwhile, natural products hold significant potential in regulating ferroptosis and maintaining ovarian function.

### 5.1 Overview of ferroptosis

Ferroptosis is an iron-dependent form of cell death driven by the accumulation of lipid peroxides (Dixon et al., 2012). It is initiated by excessive lipid peroxidation and impaired antioxidant defense mechanisms, particularly the inactivation of glutathione peroxidase 4 (GPX4), a key enzyme that reduces lipid hydroperoxides (Li J. et al., 2020). Unlike apoptosis, ferroptosis does not involve nucleus fragmentation or the formation of apoptotic bodies and is independent of classical apoptotic pathway proteins such as caspases.

Ferroptosis depends on iron-dependent lipid peroxidation, with its key signaling regulation falling into three broad categories: iron metabolism, glutathione metabolism, and lipid metabolism (Jiang et al., 2021). First, cells take up iron from the extracellular environment via transferrin (TF) and the transferrin receptor (TFRC). Once inside the cell, iron is stored in ferritin, or its release is regulated by ferroptosis-related proteins such as nuclear receptor coactivator 4 (NCO4). Increased levels of free iron generate large amounts of ROS through the Fenton reaction, which in turn promotes lipid peroxidation (Jiang et al., 2021). Secondly, glutathione metabolism plays a critical role in intracellular ferroptosis. System xc^−^ is an essential amino acid antiporter composed of two subunits, SLC7A11 and SLC3A2. SLC7A11 is the primary transporter protein, highly specific for cystine and glutamate, while SLC3A2 is a chaperone. System xc^−^ facilitates the exchange of intracellular glutamate (Glu) for extracellular cystine (Cys) in a 1:1 ratio. Cystine is then utilized to synthesize glutathione (GSH) through the enzymatic actions of glutamate cysteine ligase (GCL) and glutathione synthetase (GSS) (Wang L. et al., 2020). Glutathione peroxidase 4 (GPX4), a key enzyme in the inhibition of ferroptosis, uses GSH to convert lipid peroxides into harmless lipid alcohols (Liang et al., 2022; Xie et al., 2023). When GSH is depleted or GPX4 is inactivated, lipid peroxides accumulate, triggering ferroptosis (Liang et al., 2022). Further, polyunsaturated fatty acids (PUFAs) are essential precursors for lipid peroxidation in ferroptosis. Acyl coenzyme A synthetase long-chain family member 4 (ACSL4) and lysophosphatidylcholine acyltransferase 3 (LPCAT3) are intermediate factors essential for activating fatty acids to drive lipid peroxidation and execute ferroptosis. PUFAs are ligated to coenzyme A (CoA) by ACSL4, while LPCAT3 modifies phospholipid-linked products. Lipid peroxidation is further amplified by lipid oxygenases (LOXs) and ROS generated through the Fenton reaction, driving the process of ferroptosis (Chen et al., 2020a).

Apart from GPX4, several key intracellular factors inhibit ferroptosis. Ferroportin (FPN), the only known cellular iron efflux protein, directly reduces intracellular iron levels to inhibit ferroptosis (Bao et al., 2021). Coenzyme Q10 (CoQ10), a lipophilic electron carrier, exists in oxidized and reduced forms. Its reduced form, CoQ10H_2_, functions as a radical-trapping antioxidant (RTA) that neutralizes lipid peroxyl radicals. FSP1 (ferroptosis suppressor protein 1) facilitates the NADPH-dependent reduction of CoQ10 to CoQ10H_2_, a lipophilic radical-trapping antioxidant that prevents lipid peroxidation (Doll et al., 2019). Dihydroorotate dehydrogenase (DHODH), the rate-limiting enzyme in the pyrimidine *de novo* synthesis pathway, works alongside GPX4 to inhibit mitochondrial lipid peroxidation. It achieves this by reducing ubiquinone to ubiquinol in a coenzyme Q-dependent manner, thereby preventing ferroptosis in the inner mitochondrial membrane (Wang and Min, 2021). GTP cyclohydrolysase 1 (GCH1), the rate-limiting enzyme for BH4 synthesis, also functions as a free-radical trapping antioxidant to inhibit ferroptosis (Kraft et al., 2020). The expression of GCH1 induces the production of potent endogenous RTA, such as BH4/BH2, which block lipid peroxidation and ferroptosis. In addition, Nrf2, a widely studied transcription factor, plays an important role in regulating antioxidant responses. The downstream targets of Nrf2, including SLC7A11 and heme oxygenase 1 (HO-1), have been implicated in intracellular iron metabolism and GPX4 synthesis, both of which are essential in ferroptosis regulation (Zhang et al., 2022).

**FIGURE 4:**
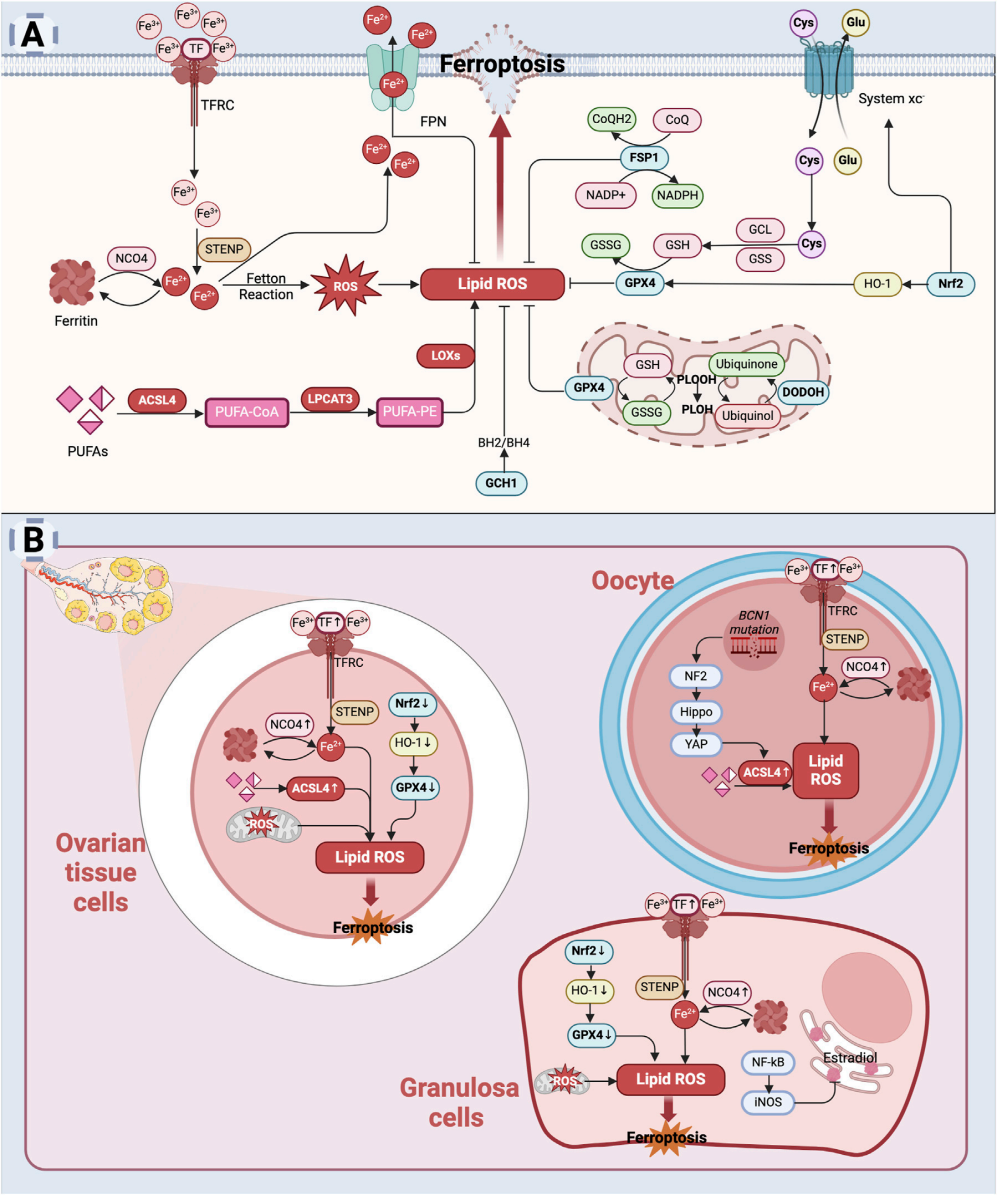
Ferroptosis and its role in diminished ovarian reserve: **(A)** Mechanism of ferroptosis: Excessive intracellular iron accumulation promotes the generation of free radicals, particularly lipid peroxides (Jiang et al., 2021). Glutathione peroxidase 4 (GPX4) plays a critical role in preventing lipid peroxidation; however, the inhibition of GPX4 activity during ferroptosis exacerbates lipid peroxidation (Liang et al., 2022; Xie et al., 2023). The accumulation of lipid peroxides causes structural damage to the cellular membrane, ultimately resulting in cell death. **(B)** Mechanism of cellular ferroptosis in diminished ovarian reserve: In the ovary, iron accumulation may increase due to aging, oxidative stress, or pathological conditions (Sze et al., 2022; Zhang et al., 2018). This excess iron triggers oxidative stress, leading to damage in ovarian cells, including oocytes and granulosa cells (Wang F. et al., 2022). Concurrently, reduced levels of glutathione (GSH) and inhibition of GPX4 activity weaken the antioxidant defense system, further promoting ferroptosis (Niu et al., 2023; Zhang S. et al., 2023). This process contributes to cellular damage and diminished ovarian reserve (Created in BioRender.com).

Taken together, ferroptosis is a PCD mechanism intricately associated with iron metabolism, lipid peroxidation, and antioxidant systems (Figure 4A). Elucidating its regulatory pathways may offer new therapeutic opportunities for improving DOR.

### 5.2 Ferroptosis and DOR

Abnormalities in iron metabolism are associated with various degenerative diseases, and granulosa cells, oocytes, and their supporting cells in the ovary are particularly sensitive to OS (Stockwell et al., 2017). Ferroptosis mediates cell damage and death primarily through lipid peroxidation. Previously, single-cell RNA sequencing of mouse ovarian oocytes during the transition from the pre-follicular stage to the follicular stage indicated that follicular atresia may be associated with the ferroptosis pathway (Wang JJ. et al., 2020). *In vitro* studies have shown that the ferroptosis inducer erastin can inhibit the proliferation of granulosa cells and promote their ferroptosis (Pan et al., 2024). This suggests that ferroptosis of ovarian cells may affect ovarian reserve function.

Several key factors influencing germ cell fate, including age, genetic variation, oxidative stress, radiation, and cancer treatment, are associated with ferroptosis. Increased ACSL4 expression and ferroptosis markers have been observed in aged rat ovaries (Zheng et al., 2021). In naturally aged rat ovaries, abnormal upregulation of transferrin (TF) and iron regulatory protein 2 (IRP2)-mediated transferrin receptor 1 (TfR1) leads to iron accumulation (Sze et al., 2022). Ferritin upregulation inhibits estradiol synthesis via the NF-κB/iNOS pathway and downregulates the Nrf2/GPX4 pathway, exacerbating ovarian damage (Sze et al., 2022). Spatial transcriptomics combined with single-cell RNA sequencing identified high expression levels of ferroptosis-related genes, including TFRC, NCO4, and SLC3A2, in granulosa cells of patients with ovarian senescence, while GPX4 expression was notably low (Lin et al., 2023). Genetic factors also influence ovarian function through ferroptosis. A study in a Chinese POI pedigree identified *BNC1* mutations that lead to premature follicular activation and atresia (Zhang et al., 2018). Further research using *BNC1* ^−/−^ mice demonstrated that *BNC1* defects promote intracellular iron accumulation and induce oocyte ferroptosis by activating the Merlin (NF2)/Hippo/YAP/TFRC/ACSL4 pathway (Wang F. et al., 2022). And ferroptosis inhibitor ferrostatin-1 (Fer-1) partially reversed this effect (Wang F. et al., 2022). Similarly, ovarian oxidative stress model mice exhibit inhibition of the Nrf2/HO-1/GPX4 pathway (Niu et al., 2023), suggesting that GPX4 deficiency-induced ferroptosis may contribute to reduced ovarian reserve. Radiation is another known inducer of ovarian damage. Studies have shown that X-ray irradiation reduces KGN cells viability, alters mitochondrial morphology, induces intracellular iron accumulation, and triggers lipid peroxidation, ultimately leading to ferroptosis (Zhao et al., 2023). The chemotherapeutic drug cisplatin (Cis) also induces ferroptosis in granulosa cells, causing ovarian fibrosis and significantly reducing ovarian reserve (Zhang S. et al., 2023; Wang et al., 2024). Cis increases ACSL4 expression while suppressing GPX4 expression and promotes mitochondrial dysfunction through excessive ROS production (Zhang S. et al., 2023; Wang et al., 2024). Further studies revealed that treatment with N-acetylcysteine (NAC), a GSH precursor, significantly upregulates the GSH/GSSG ratio and increases the expression of GPX4, NRF2, and HO-1, effectively inhibiting ferroptosis (Zhang S. et al., 2023). Similarly, the antitumor drug CTX induces ferroptosis in granulosa cells both *in vivo* and *in vitro* by suppressing GPX4 expression (Niu et al., 2023; Chen et al., 2024; Dai et al., 2024).

Overall, there is a strong association between common causes of ovarian reserve loss, such as aging, genetic variation, OS, radiation and chemotherapy, and ferroptosis, with disturbances in intracellular iron metabolism playing a central role in this process. Ferroptosis contributes to follicular depletion, ultimately leading to DOR (Figure 4B). Future research focusing on ferroptosis-related pathways may provide innovative therapeutic strategies for restoring ovarian function and preserving fertility.

### 5.3 Regulation of ferroptosis by natural products

In recent years, ferroptosis has emerged as a critical focus in drug development. Ferroptosis mediates cell injury and death through lipid peroxidation, which adversely affects follicular survival. Preliminary studies suggest that inhibiting ferroptosis may offer protective effects on ovarian function, although the translational relevance of these findings remains to be established.

Current research has found that ferroptosis caused by the downregulation of the Nrf2/HO-1/GPX4 pathway is closely associated with diminished ovarian reserve (Niu et al., 2023; Zhang S. et al., 2023). However, many natural plant metabolites have been reported to inhibit ferroptosis by activating this pathway. The flavonoid glycoside icariin has been shown to increase the expression of Nrf2, HO-1, and GPX4, in cisplatin-treated mouse ovarian tissues and KGN cells, which may contribute to reduced OS and ferroptosis (Li F. et al., 2024). *In vitro* experiments have demonstrated that another natural flavonoid glycoside, rutin, may alleviate oxidative stress induced by ferroptosis in small white follicles of aging laying chickens via the Nrf2/HO-1 pathway (Wu Y. et al., 2023). Similarly, the methylated derivative of resveratrol, pterostilbene, has been observed to upregulate Nrf2, HO-1, GSH, and GPX4 while reducing ACSL4 expression and iron content in H_2_O_2_-treated COV434 and KGN cells, suggesting a potential anti-ferroptotic effect (Chen et al., 2023c).

In addition to the Nrf2/HO-1/GPX4 pathway, other pathways have been implicated. Spermidine may regulate the Akt/FHC/ACSL4 pathway to inhibit ferroptosis in porcine ovarian granulosa cells, with possible implications for ovarian reserve and fertility (Niu et al., 2023). Another naturally occurring sphingolipid, sphingosine-1-phosphate, has been shown *in vitro* to modulate the expression of GPX4 and FPN1, and TFRC, which may contribute to reduce radiation-induced ferroptosis in KGN cells (Zhao et al., 2023). Furthermore, female patients with hepatolenticular degeneration (HLD) often experience reproductive dysfunction (Roseira et al., 2022; Bandmann et al., 2015). Berberine, a natural alkaloid, has been studied in the context of hepatolenticular degeneration (HLD), where it was associated with decreased ovarian iron content and ER stress (via the PERK pathway), along with increased GSH and GPX4 expression (Liu Q. Z. et al., 2024). These molecular changes coincided with histological improvements in ovarian morphology; however, the precise mechanisms and functional consequences remain to be elucidated.

In conclusion, six natural products have been investigated for their potential anti-ferroptotic effects in the context of ovarian protection (Supplementary Appendix 3). Among them, the study of Niu et al. is the only one that combines *in vitro* and *in vivo* models (Niu et al., 2023). However, this study—like most others in the field—lacked gene-level functional validation, such as gene silencing or overexpression experiments. The majority of existing research remains limited to *in vitro* cellular assays and relies primarily on indirect markers, including GPX4 expression changes or the use of pharmacological inhibitors, to infer mechanistic pathways. Furthermore, no studies to date have reported pharmacokinetic parameters (e.g., absorption, bioavailability, tissue distribution), nor have they conducted long-term toxicity assessments or evaluated reproductive outcomes *in vivo*. These methodological gaps substantially limit the interpretation, reproducibility, and translational potential of the current findings. Although ferroptosis has emerged as a potentially valuable target in ovarian-related therapeutic research, current investigations into the regulatory effects of natural products on ferroptosis remain preliminary. Future studies should emphasize comprehensive mechanistic validation—such as gene-level modulation—alongside *in vivo* efficacy testing and translational assessments, including pharmacokinetic profiling and toxicological evaluation. These efforts will be essential to determine the feasibility and relevance of natural compounds as modulators of ferroptosis in the context of ovarian function.

## 6 Natural products targeting pyroptosis in cells with DOR

Pyroptosis is also considered an important mechanism contributing to ovarian function decline. Pyroptosis is a highly inflammatory form of cell death that can be abnormally activated by oxidative stress, endoplasmic reticulum stress, and pro-inflammatory cytokines, thereby impairing ovarian function. Below, we introduce the inhibition of pyroptosis as a novel strategy for improving DOR.

### 6.1 Overview of pyroptosis

Pyroptosis is a lytic and pro-inflammatory form of programmed cell death often associated with infections and immune responses. It is initiated by the activation of inflammasomes, which activate inflammatory caspases (such as caspase-1, -4, -5, and -11), leading to cleavage of Gasdermin D (GSDMD). These caspases cleave GSDMD, releasing its N-terminal fragment. This fragment oligomerizes and inserts into the plasma membrane, forming pores that disrupt membrane integrity, increase ion influx, and lead to cell swelling and eventual rupture (Kovacs and Miao, 2017). During this rupture, pro-inflammatory cytokines such as IL-1β and IL-18 are released, triggering local or systemic inflammatory responses. The affected cells rapidly absorb water, swell, and rupture their membranes (Kovacs and Miao, 2017). Unlike the DNA ladder fragmentation observed in apoptosis, DNA degradation in pyroptotic cells is fragmented but not orderly.

**FIGURE 5:**
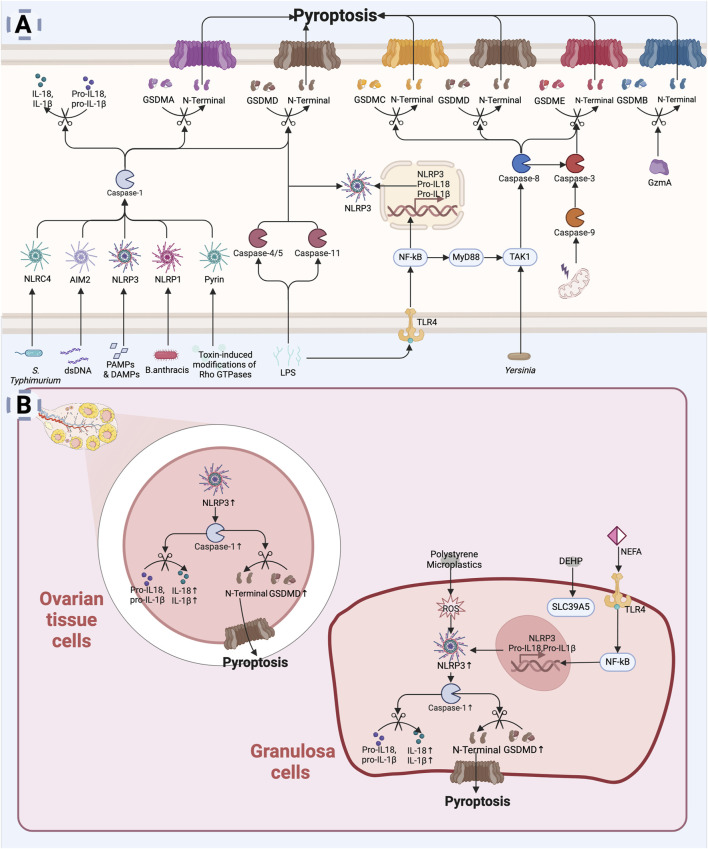
Pyroptosis leading to diminished ovarian reserve: **(A)** Mechanism of pyroptosis. Pyroptosis is initiated by the activation of inflammasomes (e.g., NLRP3, AIM2) in response to infection or cellular injury (Frank and Vince, 2019). Activated inflammasomes contribute to the activation of proteases such as caspase-1. Caspase-1 cleaves the N-terminal structural domain of the GSDMD to insert into the cell membrane, forming a pore that leads to rupture of the membrane and release of the cellular contents (Kovacs and Miao, 2017). At the same time, caspase-1 promotes the processing, maturation and release of IL-1β and IL-18 (Kovacs and Miao, 2017). LPS can directly activate Caspase-4/5 (human) or Caspase-11 (mouse) in host cells to cleave GSDMD (Sahoo et al., 2023). Caspase-8, Caspase-3, and GzmA are also involved in GSDM protein cleavage leading to cellular pyroptosis (Orning et al., 2018; Zhang JY. et al., 2021; Wang et al., 2021; Wang et al., 2017; Zhou et al., 2020). **(B)** Mechanisms of pyroptosis in diminished ovarian reserve. In the ovary, environmental pollutants, oxidative stress, or chronic inflammation can activate inflammasomes (e.g., NLRP3), leading to intracellular signaling (Navarro-Pando et al., 2021; Lliberos et al., 2021b; Khallaf et al., 2023; Chi et al., 2023; Liu K. et al., 2024; Xie et al., 2024). Pyroptosis not only directly affects the survival of granulosa cells, but may also lead to changes in the ovarian microenvironment, affecting ovarian reserve function (Zhou C. et al., 2024) (Created in BioRender.com).

Pyroptosis can be initiated via two pathways: classical and non-classical. The classical pyroptosis pathway is triggered by pathogen-associated molecular patterns (PAMPs) or damage-associated molecular patterns (DAMPs) recognized by specific receptors (Frank and Vince, 2019), leads to the assembly of inflammasomes, which then recruit and activate caspase-1, a key executioner protein in pyroptosis. Caspase-1 cleaves the N-terminal structural domain of GSDMD, and the cleaved N-terminal fragment inserts into the cell membrane, to form pores, ultimately causing membrane rupture and the release of pro-inflammatory intracellular contents. Caspase-1 also processes and promotes the maturation and release of IL-1β and IL-18, enhancing the local inflammatory response (Kovacs and Miao, 2017). There are five main types of inflammasomes: NLRP1, NLRP3, NLRC4, Pyrin, and AIM2 (Nozaki et al., 2022). These inflammasomes can recognize different pathogens during immune responses and initiate pyroptosis. Among them, NLRP3 is a core member of the caspase-1-dependent inflammasome (Coll et al., 2022). Transcription of NLRP3 and inflammatory factors is upregulated through the Toll-like receptor (TLR)-mediated NF-κB pathway in response to various cellular stresses, including ROS, mitochondrial damage, and lysosomal destabilization, promoting inflammasome oligomerization and recruitment of other components (Coll et al., 2022). In the non-classical pathway, lipopolysaccharide (LPS) directly activates caspase-4/5 in humans or caspase-11 in mice, bypassing inflammasomes (Sahoo et al., 2023). Activated caspase-4/5/11 cleaves GSDMD, with its N-terminal fragment forming membrane pores, leading to pyroptosis. Caspase-4/5/11 can also activate the NLRP3 inflammasome, which subsequently activates caspase-1, resulting in the production and release of IL-1β and IL-18 (Sahoo et al., 2023). Additionally, alternative pyroptosis pathways exist. For example, caspase-8 cleaves GSDMC, GSDMD, and GSDME (Orning et al., 2018; Zhang JY. et al., 2021; Wang et al., 2021), while caspase-3 induces Gasdermin E (GSDME) cleavage (Wang et al., 2017). Granzyme A (GzmA) cleaves GSDMB (Zhou et al., 2020), and caspase-1 cleaves GSDMA (Billman et al., 2024). The N-terminal fragments formed by cleavage participate in pore formation in the cell membrane, ultimately leading to pyroptotic cell death. The specific cellular mechanisms of pyroptosis are illustrated in Figure 5A.

### 6.2 Pyroptosis and DOR

There is a strong relationship between pyroptosis and inflammation. With maternal aging, the ovarian microenvironment gradually transitions to a low-level inflammatory state (Lliberos et al., 2021a; Umehara et al., 2022). Single-cell RNA sequencing has shown age-related activation of the macrophage pyroptosis pathway, which accelerates stromal cell senescence and reproductive decline (Zhou C. et al., 2024). Levels of NLRP3, caspase-1, and IL-1β in granulosa cells of DOR patients (Navarro-Pando et al., 2021). In mice, specific knockout of *GSDMD* or *NLRP3* reduces ovarian pro-inflammatory cytokines (IL-6, IL-18, TNF-α), decreases caspase-1 activity, increases follicle numbers, and delays reproductive aging (Zhou C. et al., 2024; Navarro-Pando et al., 2021; Lliberos et al., 2021b). High expression of NLRP3, GSDMD, caspase-1, IL-18, and IL-1β has been observed in ovarian tissues of POI animal models (Khallaf et al., 2023; Chi et al., 2023; Liu K. et al., 2024; Xie et al., 2024). These studies offer fresh perspectives on the bidirectional regulatory mechanisms between pyroptosis and inflammation in DOR.

Additionally, aging and life stress-induced OS appear to contribute to DOR by promoting granulosa cell pyroptosis. OS can activate the NLRP3 inflammasome, triggering caspase-1 activation and pyroptosis (Bauernfeind et al., 2011; Won et al., 2015). Non-esterified fatty acids (NEFAs) activate the TLR4/NF-κB pathway in granulosa cells, increasing NLRP3 and caspase-1 levels, along with the release of IL-1β and IL-6, leading to OS, pyroptosis, and inflammation (Wang Y. et al., 2020). The addition of the antioxidant N-acetylcysteine was shown to reverse NEFA-induced OS and pyroptosis (Valckx et al., 2014; Yang et al., 2012). Environmental pollutants may also impair female reproduction via pyroptosis. Polystyrene microplastics are internalized by ovarian granulosa cells, inducing pyroptosis via OS-mediated NLRP3/caspase-1 activation (Hou et al., 2021). Similarly, di-(2-ethylhexyl) phthalate (DEHP) treatment upregulates solute carrier family 39 member 5 (SLC39A5), activating the NF-κB/NLRP3 axis and leading to ovarian dysfunction (Sun J. et al., 2023).

Moreover, high glucose levels activate the NLRP3 inflammasome, promoting pyroptosis and disrupting estradiol synthesis (Xu R. et al., 2023; Baddela et al., 2020). Pyroptosis activation during ovarian tissue cryopreservation and autotransplantation can be mitigated by pyroptosis inhibitors (Wang et al., 2019), improving ovarian function.

Collectively, age-related inflammatory states, OS, metabolic disorders, and environmental pollutants appear to contribute to ovarian dysfunction by inducing pyroptosis in granulosa cells (Figure 5B). Further investigation into the role of cellular pyroptosis in regulating follicular atresia may reveal promising therapeutic targets for delaying the onset of DOR.

### 6.3 Regulation of pyroptosis by natural products

Cellular pyroptosis, a recently discovered mode of PCD, has garnered increasing attention, in the context of DOR regulation. However, its precise role in DOR and the therapeutic potential targeting pyroptosis pathways remain underexplored. While certain natural products have shown promising results in *in vitro* models, attributing them with specific therapeutic relevance, especially in *in vivo* ovarian reserve preservation, remains speculative without clearer mechanistic evidence (Supplementary Appendix 4).

Leonurine has demonstrated the ability to inhibit NLRP3 inflammasome activation (Liu Y. et al., 2018; Chi et al., 2023). Intraperitoneal injection of leonurine was shown to improve fertility and maintained follicle numbers in cyclophosphamide-induced POI mice, primarily via the suppression of the NLRP3/caspase-1/GSDMD pathway and the reduction of IL-18 and IL-1β levels (Liu Y. et al., 2018; Chi et al., 2023). Although promising, these findings require further validation to determine whether Leonurine can be translated into a viable therapeutic option for human ovarian reserve preservation. α-Ketoglutarate, an intermediate metabolite of the tricarboxylic acid cycle, has been shown to suppress NLRP3 inflammasome activation and improve ovarian reserve in POI rat models (Liu K. et al., 2024). Similarly, quercetin and Coenzyme Q10 have been found to reverse mitochondrial dysfunction and decrease expression of pyroptosis markers in cyclophosphamide-induced POI mice (Chen et al., 2022). Their effects, while promising, are primarily observed in preclinical settings, and their clinical relevance in preserving ovarian function remains to be validated (Chen et al., 2022). Allantoin has been shown to downregulate IL-1β and caspase-1 expression (Wang et al., 2023c). Interestingly, it also upregulated NLRP3 expression, possibly due to its role in enhancing autophagy (Wang et al., 2023c).

In conclusion, five natural products have been reported to modulate pyroptosis-related pathways and improve ovarian reserve in preclinical models. Notably, all these experimental studies have certain limitations in their design. None of them evaluated the long-term toxicity, nor did they conduct gene knockdown or overexpression experiments to verify the regulatory mechanisms of natural products (Chen et al., 2022; Wang et al., 2023c; Chi et al., 2023; Liu K. et al., 2024). The study by Liu et al. did not set up gradient doses (Liu K. et al., 2024). Even in the other three studies where gradient concentrations were set, dose-response analysis was lacking. These shortcomings reduce the mechanistic clarity and translational value of the results. Further elucidation of these mechanisms may enhance our understanding of pyroptosis in DOR and aid in the development of natural product-based therapies targeting pyroptosis-related factors.

## 7 Natural products targeting necroptosis in DOR

Necroptosis has been shown to play a crucial role in various degenerative diseases. However, its specific mechanisms in DOR remain unclear. In recent years, some natural plant metabolites have been found to regulate necroptosis-related factors and may have potential value in improving ovarian reserve. In the following section, we will introduce several promising natural plant metabolites and their mechanisms of action.

### 7.1 Overview of necroptosis

Necroptosis represents a distinct form of PCD that combines features of both necrosis and apoptosis. It is mediated by specific signaling molecules, notably, including receptor-interacting protein kinases 1 and 3 (RIPK1 and RIPK3) and mixed lineage kinase domain-like protein (MLKL), which drive necroptosis by activating related signaling pathways in response to severe stress or pathological stimuli (Brault and Oberst, 2017). Morphologically, necroptosis is characterized by cell and organelle swelling, followed by plasma membrane rupture. This process also triggers a robust immune response by releasing DAMPs (Galluzzi et al., 2018).

**FIGURE 6:**
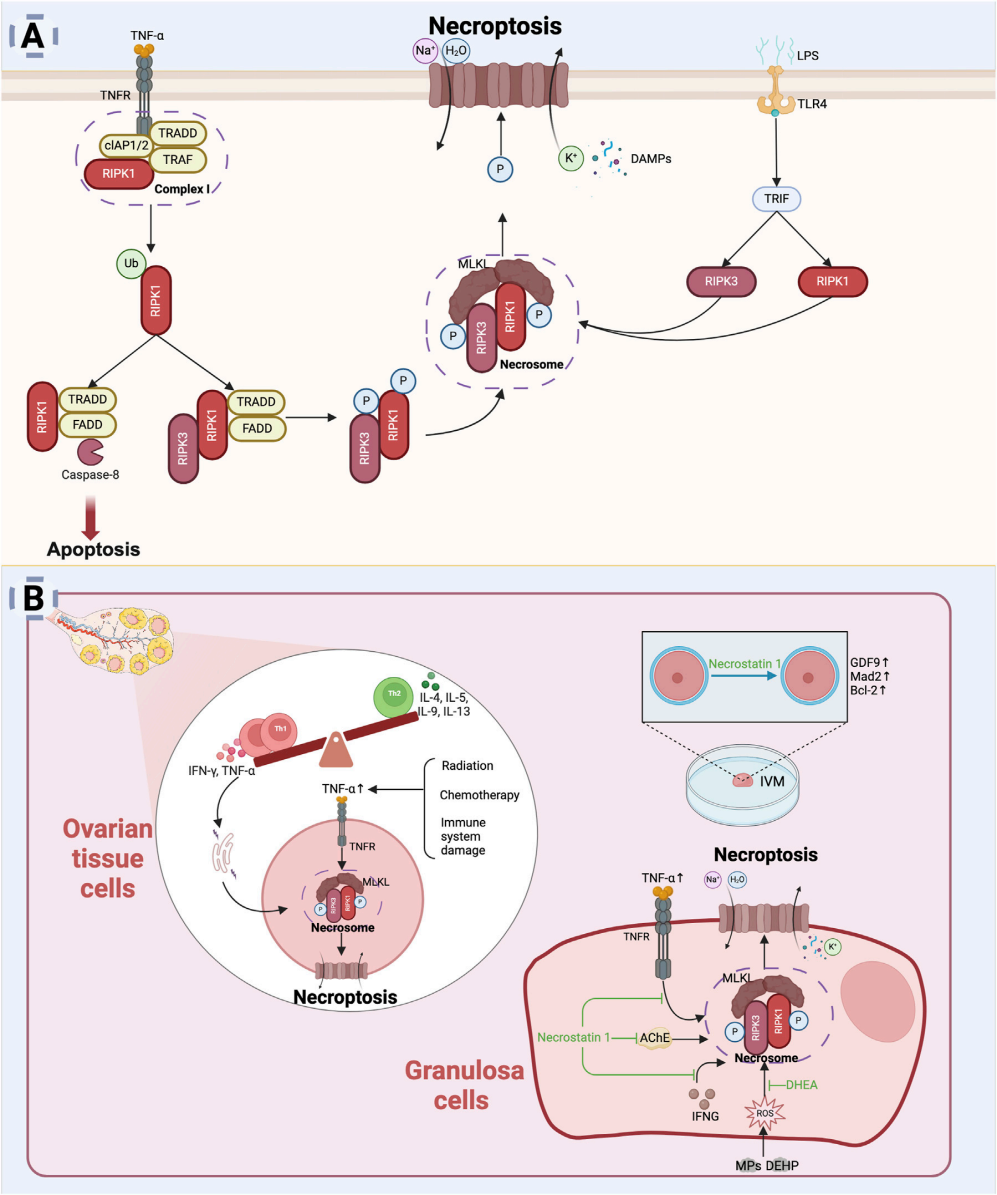
Necroptosis and diminished ovarian reserve. **(A)** Mechanism of necroptosis: TNF-α and its receptor TNFR mediate necroptosis (Nailwal and Chan, 2019), with RIPK1 phosphorylation activating RIPK3 to form the necrosome (Nailwal and Chan, 2019). RIPK3 phosphorylation activates MLKL, which translocates to the cell membrane, causing membrane rupture, leakage of cellular contents, and cell death (Murao et al., 2021). TLR4 can also induce necroptosis through TRIF interaction with the necrosome (Solon et al., 2024). **(B)** Mechanisms of necroptosis in diminished ovarian reserve: In the ovarian microenvironment, chronic inflammation or harmful stimuli may trigger TNF-α release, activating necroptotic pathways (Khallaf et al., 2023; Li X. et al., 2023; Akdemir et al., 2022; Tao et al., 2024). Necrostatin-1 promotes granulosa cells survival and improves IVM oocyte quality by inhibiting necroptosis (Hojo et al., 2016; Jo et al., 2015) (Created in BioRender.com).

TNF-α is the primary upstream mediator of in necroptosis (Newton and Manning, 2016). Under normal conditions, TNF-α signaling facilitates the recruitment of RIPK1 kinase to the plasma membrane, forming membrane-associated complex Ⅰ, which includes RIPK1, TRADD, TNF receptor-associated factor (TRAF), and cellular inhibitors of apoptosis proteins (cIAP1/2) (Holler et al., 2000). Within complex I, RIPK1 is ubiquitinated by cIAP1/2, promoting cell survival. However, under specific conditions, deubiquitinated RIPK1 dissociates from TRADD and complex I, leading to the initiation of apoptosis or necroptosis. RIPK1 can form a secondary complex with activated caspase-8, which drives apoptosis. However, during cellular stress, inhibition of caspase-8 activity shifts the regulatory role of RIPK1 from apoptosis to necroptosis (Newton and Manning, 2016). This shift facilitates the formation of the necrosome, where in RIPK1 and RIPK3 undergo auto- and cross-phosphorylation. Phosphorylated RIPK3 subsequently recruits and phosphorylates MLKL, which translocates to the plasma membrane to form pore complexes, disrupting membrane integrity. These disruptions lead to water and sodium influx, potassium efflux, cell swelling, membrane potential changes, and eventual cell lysis, which then releases intracellular contents, eliciting an inflammatory phenotype through DAMP release and triggering immune responses (Murao et al., 2021). Additionally, toll-like receptor 4 (TLR4) can induce necroptosis via interaction between the toll receptor-associated activator of interferon (TRIF) and the necrosome (Solon et al., 2024). Even in the absence of infection, DAMPs can independently activate necroptosis, eliciting a RIPK1-independent inflammatory response (Nailwal and Chan, 2019). The specific mechanisms underlying necroptosis are presented in Figure 6A.

### 7.2 Necroptosis and DOR

Necroptosis typically occurs during cellular stress, inflammation, or immune responses, all of which may accelerate the decline in ovarian reserve. Evidence indicates that the expression of RIPK1 and RIPK3 mRNA, key mediators of necroptosis, is significantly elevated in bovine atretic follicles compared with healthy follicles (McEvoy et al., 2021). TNF-α can trigger necroptosis via the RIPK1-mediated pathway, impacting oocytes development and reducing the number of mature follicles (Terranova, 1997; Marcinkiewicz et al., 2002; Bagavant et al., 1999). Necrostatin-1 (Nec-1), a necroptosis inhibitor, blocks the interaction between RIPK1 and RIPK3 during necroptosis activation. *In vitro* studies have demonstrated that Nec-1 treatment inhibits TNF-α and interferon-γ (IFNG)-mediated expression of RIPK1 and RIPK3 in bovine granulosa cells (Hojo et al., 2016). Immune damage, radiation, and chemotherapy can lead to ovarian reserve decline and increased TNF-α levels, suggesting that necroptosis may be an important mechanism behind ovarian dysfunction caused by these injury-related factors (Khallaf et al., 2023; Li X. et al., 2023; Akdemir et al., 2022; Tao et al., 2024).

Additionally, oxidative stress can induce necroptosis through ROS accumulation, further damaging oocyte quality. Poor lifestyle choices, psychological stress, and other factors promote cortisol release and elevate ROS levels (Prasad et al., 2016). In in vitro maturation (IVM) cultures, the addition of Necrostatin-1 significantly increased the expression of GDF9, Mad2, and Bcl-2 in immature oocytes in mice and prevented oxidative stress-mediated deterioration of oocyte quality (Jo et al., 2015). Prolonged starvation can also induce ROS accumulation and activate necroptosis, while DHEA successfully reverses these effects (Tsui et al., 2017). Granulosa cells both produce and respond to the neurotransmitter acetylcholine (ACh), which plays a key role in their function (Mayerhofer and Fritz, 2002). ACh regulates Ca^2+^ levels and transcription factor expression through muscarinic receptors, while acetylcholinesterase (AChE) promotes the breakdown of ACh. AChE expression is upregulated under oxidative stress and accumulates in serum with age, inducing RIPK1/MLKL-dependent necroptosis (Härtl et al., 2011; Sklan et al., 2004). Both Nec-1 and the MLKL inhibitor can block this process effectively, promoting follicular development *in vitro* from secondary to small antral stages (Du et al., 2018; Blohberger et al., 2015).

In recent years, environmental pollution has become a major concern due to its impact on human health. Long-term exposure to pollutants like microplastics (MPs) and DEHP promotes excessive ROS production through the CNR1/CRBN/YY1/CYP2E1 axis, oxidizing YAP1, which ultimately leads to necroptosis and impaired ovarian function in granulosa cells (Wu H. et al., 2023). Particulate matter in air pollution can also increase MLKL expression in ovarian tissues and reduce follicle count (Namvar et al., 2024). Exposure to cadmium (Cd) and excess molybdenum (Mo) disrupts ovarian micronutrient balance, shifts the Th1/Th2 immune balance toward Th1, and activates ER stress, triggering ovarian necroptosis through the RIPK1/RIPK3/MLKL pathway, ultimately impairing ovarian function (Cui et al., 2024).

In conclusion, external factors such as environmental pollutants, immune system damage, and stress-induced OS can trigger necroptosis. The activation of necroptotic cell death pathways in the ovary contributes to the deterioration of ovarian function, further accelerating conditions like DOR (Figure 6B).

### 7.3 Regulation of necroptosis by natural products

Research on the role of necroptosis in DOR and its potential therapeutic targeting through natural products remains limited. To date, only two natural products have been preliminarily implicated in necroptosis inhibition with potential relevance to ovarian protection (Supplementary Appendix 5). DHEA, the most abundant steroid hormone in primates, is primarily synthesized by the adrenal cortex. While DHEA can disrupt ovarian function in polycystic ovary syndrome (Yuan et al., 2016), it appears to exhibit a contrasting effect in the context of DOR. In serum starvation-induced HO-23 cells, DHEA was found to reduce the expression of necroptosis markers RIPK1 and RIPK3 (Tsui et al., 2017). However, this study neither established a dose - response relationship nor confirmed whether DHEA can truly improve ovarian function *in vivo*. Scutellarin, a flavonoid compound, was shown to mitigate zearalenone (ZEA)-induced granulosa cells apoptosis *in vitro* (Yi et al., 2021). Transcriptomic data suggested the involvement of MAPK and heat shock protein-necroptotic pathways in ZEA toxicity and the response to scutellarin (Yi et al., 2022). Interestingly, knockdown of RIPK1 exacerbated ZEA-induced toxicity but did not diminish the protective effect of scutellarin, implying potential pathway redundancy or alternative mechanisms of action. However, these findings were not validated in animal models, and key pharmacological parameters—including fertility outcomes, long-term toxicity, and pharmacokinetics—remain uncharacterized.

Currently, research on the role of natural products in regulating necroptosis remains insufficient. In contrast, the small-molecule compound Necrostatin-1 has shown potential in inhibiting necroptosis and protecting ovarian reserve. It functions by binding to the hydrophobic pocket of the RIPK1 kinase domain (Degterev et al., 2008), preventing its dimerization and necroptosis activation. This underscores the potential value of structure-based design and rational modification of natural products for targeting necroptosis, warranting further exploration in this emerging area.

## 8 The interactive mechanisms of different PCD forms in DOR

In the previous sections, we systematically analyzed the regulatory roles of various molecular patterns of PCD in the pathological process of DOR. Although these PCD forms differ in execution mechanisms and signaling pathways, they are not isolated from each other. They form a complex network of interregulation through shared mitochondrial functional networks and oxidative stress signals.

Firstly, apoptosis, autophagy, and ferroptosis primarily establish an interactive regulatory network via mitochondrial dysfunction, a key hub. During the occurrence of DOR, the deterioration of mitochondrial function and the intensification of oxidative stress provide a platform for signaling crosstalk among various PCD forms (Seli et al., 2019). Mitochondrial damage can trigger apoptosis cascades through BAX/BAK oligomerization (Zhu et al., 2016; Zhu et al., 2015), while also increasing lipid peroxidation levels, which synergistically induces ferroptosis (Zhang S. et al., 2023). Additionally, under normal conditions, mitophagy helps suppress PCD by clearing damaged mitochondria. However, during ovarian aging, defects in PINK1/Parkin-mediated mitophagy further promote ferroptosis and apoptosis (Khan et al., 2023; Zhang Y. et al., 2023). Moreover, under oxidative stress, although autophagy initiation signals are activated in granulosa cells, impaired lysosomal acidification and mitophagy defects disrupt autophagic flux (Xu G. et al., 2023; Zheng et al., 2023). This blockage in autophagic flux ultimately leads to BAX/BAK oligomerization, activation of Caspase-3-mediated apoptosis, as verified in a POF model rat (Zhou et al., 2023). Notably, the ROS generated during ferroptosis further exacerbate mitochondrial damage (Jiang et al., 2021), forming a positive feedback loop of ‘mitochondrial dysfunction-ferroptosis-excessive ROS-mitochondrial damage’. These studies emphasize the ovarian reserve decline induced by PCD.

Secondly, complex signaling interactions and transformations occur between apoptosis, necroptosis, and pyroptosis, with Caspase-8 serving as a critical switch point. Activation of Caspase-8 promotes Caspase-3-mediated apoptosis (Legembre et al., 2002), while inhibiting the RIPK1/RIPK3 axis (necroptosis) (Newton and Manning, 2016) and the NLRP3/Caspase-1 axis (pyroptosis) (Frank and Vince, 2019). When Caspase-8 is inhibited, cells are more prone to shift toward necroptosis or pyroptosis. Mitochondrial dysfunction can induce Cyt C release (promoting apoptosis) (Zhu et al., 2016; Zhu et al., 2015), NLRP3 inflammasome activation (promoting pyroptosis) (Hou et al., 2021), and RIPK1 activation (promoting necroptosis) (Jo et al., 2015), accelerating granulosa cell death. Overall, these three forms of cell death interact and transform during DOR progression, collectively driving the loss of follicular granulosa cells and the decline of ovarian reserve.

Additionally, different forms of PCD may dominate at different stages of DOR progression, but research on their temporal sequence remains limited. For instance, early oxidative stress may primarily induce mitochondrial damage and ferroptosis, subsequently triggering compensatory activation of mitophagy (Gross, 2024). However, due to blocked autophagic flux, this eventually leads to the initiation of apoptosis or pyroptosis (Xu G. et al., 2023; Zheng et al., 2023). Notably, dynamic transitions between PCD forms may be regulated by molecules such as p53, Beclin-1, and GPX4. For example, p53 can induce apoptosis and ferroptosis under DNA damage conditions (Suh et al., 2006; Gonfloni et al., 2009; Kerr et al., 2012; Liu and Gu, 2022). Beclin-1 promotes autophagy but can also facilitate ferroptosis by inhibiting System Xc^−^ activity (Cao et al., 2017; Sun et al., 2018; Song et al., 2018). GPX4 prevents ferroptosis by reducing phospholipid peroxidation (Liang et al., 2022). GPX4 deficiency can induce apoptosis, necroptosis, and pyroptosis in certain cells (Xie et al., 2023). However, specific studies on the roles of these key molecules at different stages of DOR progression are still lacking.

Therefore, future research should further explore the mechanisms of PCD transitions, key regulatory factors, and the influence of the ovarian microenvironment on PCD interactions. This will help reveal the core molecular events underlying DOR progression and provide new intervention strategies for targeting PCD-mediated granulosa cells dysfunction.

## 9 Comparative study of natural products regulating PCD in the treatment of DOR

In previous sections, we systematically discussed the role of natural products in regulating PCD processes related to DOR. However, most studies focus on the mechanisms of individual natural products, lacking direct comparative studies. Only three studies have compared the relative efficacy of different natural products in PCD pathways. For example, quercetin and coenzyme Q10 both have been reported to exert inhibitory effects on pyroptosis and apoptosis in a CTX-induced ovarian injury animal model (Chen et al., 2022). These effects may involve the modulation of mitochondrial function, inhibition of NLRP3 inflammasome activation, and regulation of the BAX/BCL-2 pathway. However, conclusive evidence regarding their relative effectiveness in preserving ovarian reserve is lacking, and the precise molecular mechanisms remain to be fully elucidated. Additionally, combined *Citrus × limon (L.) Osbeck* peel extract and resveratrol have been shown to reduce iNOS/Caspase-3-mediated apoptosis in a CTX-induced POI model, regulate oxidative stress markers, and restore hormone levels (Mobasher et al., 2022). Moreover, the combined treatment of these two agents proved more effective than single interventions, suggesting potential synergistic effects (Mobasher et al., 2022). Furthermore, capsaicin combined with quercetin has been suggested to attenuate the inflammatory response through the TRPV1 signaling and reduce oxidative stress and P53-related apoptotic signaling, potentially contributing to improved ovarian function (Saman et al., 2023). Despite these promising findings, current evidence is preliminary, and the mechanistic basis for any comparative or synergistic advantages remains speculative. Robust, head-to-head studies are needed to clarify the therapeutic potential and mechanistic diversity of these compounds before their clinical application in DOR management can be meaningfully assessed.

## 10 Focus on natural products in assisted reproductive technology

DOR-associated infertility is primarily linked to a reduction in both the number and quality of oocytes, which significantly limits a woman’s ability to conceive (Steiner et al., 2017). In reproductive clinics, controlled ovarian hyperstimulation is recommended for patients with DOR to rapidly obtain usable embryos. However, despite advancements in *in vitro* fertilization and embryo transfer (IVF-ET) techniques, the challenges of retrieving a low number of oocytes and obtaining low-quality oocytes persist, presenting ongoing difficulties for the reproductive medicine community (Butts et al., 2014; Butts et al., 2013). There is a growing interest in the potential benefits of adjuvant therapeutic approaches to optimize infertility treatments. In 2024, the results of a systematic review showed that among women with DOR, only controlled ovarian stimulation protocols with the use of testosterone as an adjunct therapy demonstrated better clinical outcomes in terms of achieving pregnancy (Conforti et al., 2025).

Current research indicates that four natural metabolites [coenzyme Q10 (Xu et al., 2018), DHEA (Wiser et al., 2010; Kara et al., 2014; Yeung et al., 2014; Zhang et al., 2014; Tartagni et al., 2015; Kotb et al., 2016; Narkwichean et al.,2017), melatonin (Bao et al., 2022; Espino et al., 2019; Fernando et al., 2018), and resveratrol (Conforti et al., 2024)] may be helpful in improving ART outcomes (Table 2). Oral administration of coenzyme Q10 at a dose of 600 mg/day for 2 months prior to IVF treatment was found to increase the number of high-quality embryos in women with poor ovarian reserve and aged < 35 years old (Xu et al., 2018). DHEA is typically administered orally at 75 mg/day for 2–6 months before ovarian stimulation, although its effectiveness varies across clinical trials (Wiser et al., 2010; Kara et al., 2014; Yeung et al., 2014; Zhang et al., 2014; Tartagni et al., 2015; Kotb et al., 2016; Narkwichean et al., 2017). Two studies reported that DHEA contributed to increased live birth rates (Wiser et al., 2010; Tartagni et al., 2015). Melatonin is used before ovarian stimulation through to egg retrieval, but the dosage and effectiveness are highly variable (Espino et al., 2019; Fernando et al., 2018). Melatonin added at a concentration of 10^−7^ M in IVF culture media has been shown to improve clinical pregnancy rates (Bao et al., 2022). Resveratrol, administered at 150 mg/day for 3 months before IVF in women aged > 35 years with good ovarian reserve (AMH > 1.2 ng/mL), did not show any improvement in clinical pregnancy rates or live birth rates (Bao et al., 2022).

**TABLE 2:** Clinical application of natural products as supplements for the treatment of DOR.

Natural product	Type of research	Patients	Number of cases	Protocol	Treatment	Results	References
Coenzyme Q10	Multi-center randomized controlled trial	Poor ovarian reserve women with age < 35 years old	169 patients (76 treated with CoQ10 vs. 93 controls)	IVF, ICSI	Coenzyme Q10 600 mg/d for 60 days via p.o	Statistically significant increases in number of retrieved oocyte, fertilization rate, high-quality embryos, available cryopreserved embryos, and a statistically significant decrease in cancelled embryo transfer rate	Xu et al. (2018)
Dehydroepiandrosterone	Single-center randomized controlled trial	Poor ovarian reserve women	33 patients (17 treated with DHEA vs. 16 controls)	IVF	DHEA 75 mg/d for 6 or 16–18 weeks via p.o	A significantly higher live birth rate and improved embryo quality	Wiser et al. (2010)
Dehydroepiandrosterone	Single-center randomized controlled trial	DOR patients	208 patients (104 treated with DHEA vs. 104 controls)	IVF, ICSI	DHEA 75 mg/d for 12 weeks via p.o	No statistical difference	Kara et al. (2014)
Dehydroepiandrosterone	Single-center randomized controlled trial	Poor ovarian reserve women	32 patients (16 treated with DHEA vs. 16 controls)	IVF	DHEA 75 mg/d for 12 weeks via p.o	No statistical difference	Yeung et al. (2014)
Dehydroepiandrosterone	Single-center randomized controlled trial	DOR patients	95 patients (42 treated with DHEA vs. 53 controls)	IVF	DHEA 75 mg/d for 3 months via p.o	Statistically significant increases in BMP-15 in follicular fluid, and the accumulated score of embryos	Zhang et al. (2014)
Dehydroepiandrosterone	Single-center randomized controlled trial	Infertile women aged 36–40 years with normal ovarian reserve	109 patients (53 treated with DHEA vs. 56 controls)	IVF	DHEA 75 mg/d 8 weeks before starting the IVF cycle and during treatment via p.o	A significantly higher live birth rate, and a significantly decrease in miscarriage rate	Tartagni et al. (2015)
Dehydroepiandrosterone	Multi-center randomized controlled trial	Poor ovarian reserve women	140 patients (70 treated with DHEA vs. 70 controls)	IVF, ICSI	DHEA 75 mg/d for 12 weeks via p.o	Statistically significant increases in the number of oocytes, fertilization rate, fertilized oocytes, clinical pregnancy rate and ongoing pregnancy rate	Kotb et al. (2016)
Dehydroepiandrosterone	Single-center randomized controlled trial	Poor ovarian reserve women	52 patients (27 treated with DHEA vs. 25 controls)	IVF	DHEA 75 mg/d for 12 weeks via p.o	No statistical difference	Narkwichean et al. (2017)
Dehydroepiandrosterone	Multi-center randomized controlled trial	Poor ovarian reserve women	811 patients (410 treated with DHEA vs. 411 controls)	IVF	DHEA 75 mg/d for 4–12 weeks via p.o	No statistical difference	Wang et al. (2022c)
Melatonin	Multi-center randomized controlled trial	Repeated-poor-quality-embryo patients	42 patients (48 melatonin cycles vs. 133 non-melatonin cycles)	IVF	10^−7^ M melatonin added to the culture medium	A statistically significant increase in high-quality embryos, the rate of available blastocysts and clinical pregnancy rate	Bao et al. (2022)
Melatonin	Single-center randomized controlled trial	Patients with unexplained infertility	30 patients (10 took a daily dose of 3 mg of melatonin, 10 took a daily dose of 6 mg of melatonin, 10 did not take melatonin)	IVF	Melatonin 3 mg/day or 6 mg/day	Statistically significant increases in average retrieved oocytes, mature oocytes, and fertilized oocytes	Espino et al. (2019)
Melatonin	Single-center randomized controlled trial	Infertile women	160 patients (41 took a daily dose of 4 mg of melatonin, 39 took a daily dose of 8 mg of melatonin, 40 took a daily dose of 16 mg of melatonin, 40 did not take melatonin)	IVF, ICSI	Melatonin 4 mg/day, 8 mg/day or 16 mg/day	No statistical difference	Fernando et al. (2018)
Resveratrol	Single-center randomized controlled trial	Women > 35 years with good ovarian reserve (AMH > 1.2 ng/mL)	70 patients (37 treated with resveratrol vs. 33 controls)	IVF	Resveratrol supplementation 150 mg/d for 3 months via p.o	A statistically significant increase in the follicle output rate (FORT) and follicle-to oocyte index (FOI)	Conforti A et al. (2024)

**FIGURE 7:**
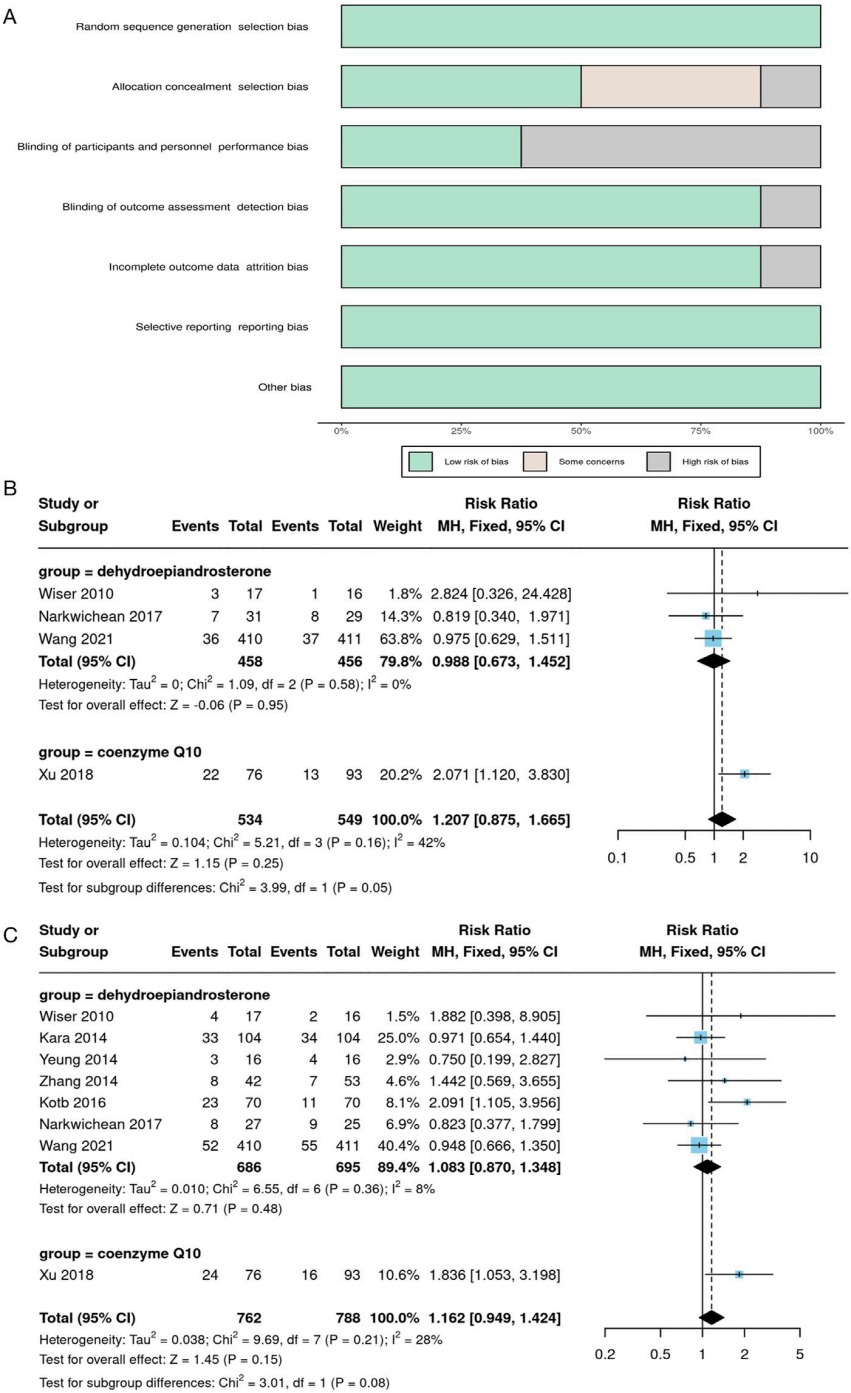
Meta analysis results of natural product treatment for DOR. **(A)** Risk bias analysis; **(B)** Forest Plot of live birth rate; **(C)** Forest Plot of clinical pregnancy rate.

In the meta-analysis of this study, eight randomized controlled trials (RCTs) met the inclusion criteria, all of which examined the effects of coenzyme Q10 or DHEA on ART outcomes in DOR/POR patients (Xu et al., 2018; Wiser et al., 2010; Kara et al., 2014; Yeung et al., 2014; Zhang et al., 2014; Kotb et al., 2016; Narkwichean et al., 2017; Wang Z. et al., 2022). Studies involving melatonin and resveratrol were excluded due to high heterogeneity in their populations and because they were not specifically focused on DOR/POR patients (Bao et al., 2022; Espino et al., 2019; Fernando et al., 2018; Conforti et al., 2024). Unfortunately, supplementation with DHEA or coenzyme Q10 did not show higher live birth rates (odds ratio: 1.207, 95% confidence interval [CI] = 0.875–1.665, P = 0.25) or clinical pregnancy rates (odds ratio: 1.162, 95% CI = 0.949–1.424, P = 0.15) compared to women who did not receive supplementation (Figure 7). It is important to note that the timing of use, dosage, and specific target populations for these natural products in IVF treatments lack standardized protocols, which may hinder the ability to draw definitive conclusions. Further research is needed to determine their impact on clinical treatment outcomes.

## 11 Outlook

Delayed childbearing has become a global trend due to economic and social developments. At the same time, the detrimental effects of adverse factors such as aging, obesity, smoking, alcohol consumption, psychological stress, and environmental or occupational exposures on female ovarian function are increasingly recognized (Sharma et al., 2013). Fertility preservation has thus emerged as a critical global health concern. Throughout a woman’s reproductive lifespan, only about 400 primordial follicles progress to preovulatory follicles and undergo ovulation, releasing oocytes capable of fertilization. The process of follicle formation and selection are highly complex, and it is widely accepted that various types of PCD are involved. Premature depletion of ovarian reserve leads to an early cessation of ovulation and subsequent infertility—events that are intricately associated with the activation of PCD pathways (Marcozzi et al., 2018).

Current clinical studies on DOR primarily focus on genome sequencing and mutation identification, while the underlying disease mechanisms and signaling pathways remain underexplored. Encouragingly, *in vitro* and *in vivo* models have facilitated the identification of several PCD-related genes and regulatory pathways implicated in DOR. Apoptosis, the most common form of PCD, is particularly significant, as granulosa cells apoptosis deprives oocytes of the hormones and survival factors essential for their meiotic and developmental competence, which increases the susceptibility of oocytes to apoptotic factors, ultimately impairing ovarian reserve function. Autophagy exhibits a dual role in DOR, influencing follicular development and atresia in different ways. Ferroptosis, on the other hand, accelerates follicular depletion and is closely associated with common adverse factors implicated in DOR, such as aging, genetic mutations, OS, radiation, and cancer therapies. While the mechanisms of pyroptosis and necroptosis in DOR are less understood, they are known to contribute to follicular atresia. Natural products that modulate PCD have shown promise in alleviating DOR symptoms through several PCD-dependent mechanisms, offering new directions for clinical research and treatment.

It is important to acknowledge that several challenges and controversies remain unresolved. Current DOR research primarily relies on animal models, such as aged mice, spontaneously aging rats, and chemotherapy-induced DOR models, as well as *in vitro* granulosa cell cultures. However, due to physiological differences between species, future studies should further optimize *in vitro* and organoid models of DOR to better replicate the human ovarian microenvironment. Additionally, integrating gene editing and single-cell sequencing technologies will be crucial for systematically elucidating the specific roles of PCD-related genes in the progression of DOR. Current knowledge of PCD is largely based on hallmark signaling pathways, such as BAX-mediated mitochondrial apoptosis and GPX4-mediated ferroptosis. However, different PCD pathways exhibit crosstalk and mutual regulation. For example, autophagy can induce apoptosis (Mariño et al., 2014), and ferroptosis can trigger autophagy (Park and Chung, 2019). Accurately measuring a specific form of PCD remains challenging. Therefore, future research should explore the interplay among various PCD pathways, identify key molecular nodes, and leverage multi-omics technologies to construct a comprehensive PCD regulatory network associated with DOR. Until an efficient and precise method for detecting PCD is established, its value as a diagnostic and therapeutic evaluation marker for DOR will remain underappreciated.

Understanding the relationship between PCD and declining fertility remains a significant challenge. In certain genetic disorders, such as TP63 and CLPP mutations, dysregulated PCD is associated with infertility as a clinical manifestation (Huang et al., 2023; Yuan et al., 2023). Additionally, environmental exposures—including air pollution and chemical toxins—have been linked to premature menopause, premature ovarian failure, and reduced fertility (Sharma et al., 2013). Unhealthy lifestyle factors—such as extreme dieting, sedentary behavior, and smoking—can contribute to ovarian dysfunction. These detrimental factors, which damage ovarian tissue, can influence multiple PCD pathways. For example, cigarette smoke exposure has been shown to reduce ovarian reserve in mice by inducing apoptosis (Xu et al., 2024; Gannon et al., 2013). Similarly, cyclophosphamide, a widely used chemotherapeutic agent, inhibits autophagy while simultaneously triggering apoptosis and pyroptosis, ultimately leading to DOR (Liu W. et al., 2021; Chen et al., 2024; Chi et al., 2023). Moreover, different forms of PCD are intricately interconnected, collectively shaping follicular fate. For instance, autophagy in granulosa cells is predominantly observed in medium-sized porcine follicles (2–6 mm in diameter), whereas apoptosis is more commonly seen in large follicles (>6 mm in diameter) (Zheng et al., 2019). Autophagy suppresses the induction of apoptosis, while the activation of apoptosis-associated cystathionase halts the autophagic process (Mariño et al., 2014). In certain scenarios, autophagy or autophagy-related proteins may paradoxically promote cellular apoptosis or necrosis. This interplay highlights that dysregulated PCD is likely a key contributor to the pathogenesis of DOR, rather than a singular causative factor. DOR, a form of reproductive dysfunction often resulting from prolonged exposure to adverse factors, cannot likely be treated by targeting a single PCD pathway. Therefore, the intricate relationship between PCD-induced reproductive dysfunction and genetic, environmental, and lifestyle factors warrants further investigation. To elucidate the role of PCD in DOR development, it is essential to conduct long-term epidemiological studies, establish correlations between reproductive health and PCD-related biomarkers, and develop predictive models. Ultimately, these efforts should guide the development of targeted preventive strategies and more precise interventions to protect fertility.

Currently, natural products remain a critical source for the discovery of novel therapeutics for various diseases. In this review, we systematically summarize the latest research on the regulation of PCD by natural products in DOR-related animal and cell models, highlighting their potential roles in preserving oocyte quality and mitigating ovarian function decline. These preliminary findings contribute to a deeper understanding of the biological processes underlying DOR and may offer a theoretical basis for the future development of targeted therapeutic strategies. Compared with chemically synthesized drugs, natural product-derived therapeutics offer significant advantages, including superior biocompatibility and functional diversity, attributes shaped by evolutionary optimization through long-term natural selection (Chen et al., 2020b). However, natural products also present challenges such as structural complexity, poor stability, and limited bioavailability (Yao et al., 2017). Moreover, most natural products interact with multiple protein targets, exerting intricate biological effects *in vivo* (Wang Z. et al., 2023). With ongoing advancements in screening technologies, chemical modification techniques, and drug delivery systems, future research should aim to optimize the structure and function of natural products to improve their bioavailability, specificity, and therapeutic index. Additionally, the adoption of standardized DOR models would facilitate more consistent and reproducible evaluations across studies. Head-to-head comparisons and combination therapy investigations are particularly needed to better understand the relative efficacy and potential synergistic interactions among different natural products. Such approaches could ultimately support their clinical translation and expand therapeutic options in reproductive medicine.

Before clinical application, the safety of natural products should be a primary concern. Current research on the regulation of PCD by natural products primarily focuses on their short-term effects, while comprehensive safety evaluations for long-term use remain insufficient. Although few studies have examined the potential side effects of natural products, some experimental data indicate that they may exhibit dose-dependent cytotoxicity. For instance, 5 μg/mL of grape seed extract (GSE) inhibited d-galactose-induced apoptosis *in vitro*, but higher concentrations (50 and 100 μg/mL) of GSE and GSPB2 for 24 h reduced the survival rates of hGC cells and KGN cell lines by approximately 20% and 50%, respectively (Barbe et al., 2019). Additionally, resveratrol has been shown to inhibit apoptosis, promote autophagy, and restore ovarian reserve in mice. However, resveratrol administration during pregnancy led to a 42% increase in fetal pancreatic mass in nonhuman primates (Roberts et al., 2014). Given the complexity of the female reproductive system, it is essential to thoroughly investigate the effects of natural products on maternal metabolic processes before clinical use. Furthermore, individual metabolic differences and potential interactions between natural products and ART-related drugs are critical factors that require careful consideration. Future large-scale, long-term observational studies should be conducted to assess the potential adverse effects of natural products and ensure their safety and efficacy in clinical applications. Furthermore, systematic investigations into the pharmacological interactions between natural products and ART-related drugs is necessary to optimize their combined application strategies.

Currently, there are a small number of randomized controlled trials exploring the effects of natural products as adjuncts to IVF treatment. Due to inconsistencies in DOR diagnostic criteria, small sample sizes, and limited follow-up durations, the results should be interpreted with caution. In 2016, the POSEIDON criteria introduced a new stratification system based on oocyte quantity and quality to define poor ovarian reserve, but implementing large randomized controlled trials using this criterion presents significant challenges (Poseidon Group Patient-Oriented Strategies Encompassing IndividualizeD Oocyte Number et al., 2016). Furthermore, poor ovarian response, characterized by low follicular sensitivity to hormonal signals and impaired ovulation, does not fully capture the reduction in follicular numbers associated with DOR. These definitional differences may affect the applicability of studies. Our meta-analysis indicates that while supplementation with DHEA and CoQ10 did not significantly improve live birth or clinical pregnancy rates in unselected DOR/POR patients, these supplements may still hold clinical value for specific patient subgroups. Future research should focus on precise patient stratification, optimizing dosage and treatment timing, and exploring their potential for combination with ART. Establishing standardized usage protocols will be crucial for successful clinical translation. Future clinical trials should refine DOR diagnostic criteria and advocate for the standardized use of the POSEIDON scoring system to stratify IVF patients, thereby reducing heterogeneity. To enhance the quality of evidence, larger sample sizes and multi-center, double-blind RCTs should be conducted. Additionally, standardized protocols for integrating natural products with ART should be developed, clearly defining optimal dosages, administration timing, and treatment durations. To ensure a comprehensive evaluation of the long-term effects of natural products on ovarian function, pregnancy outcomes, and offspring health, trials should incorporate a follow-up period of at least 12 months. Furthermore, the integration of omics technologies and biomarker-based screening strategies may aid in identifying patient subgroups most likely to benefit from natural product-based interventions, ultimately enabling more precise and personalized IVF management for DOR patients.

In conclusion, a growing body of evidence suggests that various natural products may exert beneficial effects on DOR by modulating PCD-related pathways. We call for more comprehensive studies to deepen our understanding of how PCD influences ovarian reserve function in both physiological and pathological conditions. Future research should also prioritize the identification and optimization of natural compounds with favorable pharmacokinetic profiles, low toxicity, and minimal risk to reproductive function. While preliminary findings are encouraging, the integration of PCD-targeting natural products into therapeutic strategies for DOR remains in the exploratory stage. Rigorous preclinical and clinical evaluations will be essential to determine their safety, efficacy, and potential contribution to improving reproductive outcomes in patients with DOR.

## Abbreviations

ART, assisted reproductive technology; IVF, *in vitro* fertilization; ET, embryo transfer; IVM, *in vitro* maturation; DOR, diminished ovarian reserve; POI, premature ovarian insufficiency; POF, premature ovarian failure; FSH, follicle-stimulating hormone; GCs, granulosa cells; OS, oxidative stress; ROS, reactive oxygen species; MMP, mitochondrial membrane potential; PAMPs, pathogen-associated molecular patterns; DAMPs, damage-associated molecular patterns; PCD, Programmed cell death.

## References

[B1] Agwuegbo U. T. Colley E. Albert A. P. Butnev V. Y. Bousfield G. R. Jonas K. C. (2021). Differential FSH glycosylation modulates FSHR oligomerization and subsequent cAMP signaling. Front. Endocrinol. 12, 765727. 10.3389/fendo.2021.765727 PMC8678890 34925235

[B2] Akdemir Y. Akpolat M. Elmas O. Kececi M. Buyukuysal C. Cetinkaya B. (2022). Capsaicin prevents radiotherapy-induced premature ovarian failure in rats. Reprod. Fertil. Dev. 34 (3), 350–361. 10.1071/RD21235 35101163

[B3] Ashkenazi A. Dixit V. M. (1998). Death receptors: signaling and modulation. Science 281 (5381), 1305–1308. 10.1126/science.281.5381.1305 9721089

[B4] Baddela V. S. Sharma A. Vanselow J. (2020). Non-esterified fatty acids in the ovary: friends or foes? Reprod. Biol. Endocrinol. 18, 60. 10.1186/s12958-020-00617-9 32505200 PMC7275390

[B5] Bagavant H. Adams S. Terranova P. Chang A. Kraemer F. W. Lou Y. (1999). Autoimmune ovarian inflammation triggered by proinflammatory (Th1) T cells is compatible with normal ovarian function in mice. Biol. Reprod. 61 (3), 635–642. 10.1095/biolreprod61.3.635 10456839

[B6] Bai J. Wang X. Chen Y. Yuan Q. Yang Z. Mi Y. (2024). Nobiletin ameliorates aging of chicken ovarian prehierarchical follicles by suppressing oxidative stress and promoting autophagy. Cells 13 (5), 415. 10.3390/cells13050415 38474379 PMC10930417

[B7] Bandmann O. Weiss K. H. Kaler S. G. (2015). Wilson’s disease and other neurological copper disorders. Lancet Neurol. 14 (1), 103–113. 10.1016/S1474-4422(14)70190-5 25496901 PMC4336199

[B8] Bao W. D. Pang P. Zhou X. T. Hu F. Xiong W. Chen K. (2021). Loss of ferroportin induces memory impairment by promoting ferroptosis in Alzheimer’s disease. Cell. Death Differ. 28 (5), 1548–1562. 10.1038/s41418-020-00685-9 33398092 PMC8166828

[B9] Bao Z. Li G. Wang R. Xue S. Zeng Y. Deng S. (2022). Melatonin improves quality of repeated-poor and frozen-thawed embryos in human, a prospective clinical trial. Front. Endocrinol. 13, 853999. 10.3389/fendo.2022.853999 PMC9136395 35634513

[B10] Barbe A. Ramé C. Mellouk N. Estienne A. Bongrani A. Brossaud A. (2019). Effects of grape seed extract and proanthocyanidin B2 on *in vitro* proliferation, viability, steroidogenesis, oxidative stress, and cell signaling in human granulosa cells. Int. J. Mol. Sci. 20 (17), 4215. 10.3390/ijms20174215 31466336 PMC6747392

[B11] Barberino R. S. Lins T. L. B. G. Monte A. P. O. Silva R. L. S. Andrade K. O. Campinho D. S. P. (2022). Epigallocatechin-3-gallate attenuates cyclophosphamide-induced damage in mouse ovarian tissue via suppressing inflammation, apoptosis, and expression of phosphorylated Akt, FOXO3a and rpS6. Reprod. Toxicol. Elmsford N. 113, 42–51. 10.1016/j.reprotox.2022.08.010 35981663

[B12] Bassiouny Y. A. Dakhly D. M. R. Bayoumi Y. A. Hashish N. M. (2016). Does the addition of growth hormone to the *in vitro* fertilization/intracytoplasmic sperm injection antagonist protocol improve outcomes in poor responders? A randomized, controlled trial. Fertil. Steril. 105 (3), 697–702. 10.1016/j.fertnstert.2015.11.026 26690008

[B13] Bauernfeind F. Bartok E. Rieger A. Franchi L. Núñez G. Hornung V. (2011). Cutting edge: reactive oxygen species inhibitors block priming, but not activation, of the NLRP3 inflammasome. J. Immunol. Balt. Md 1950 187 (2), 613–617. 10.4049/jimmunol.1100613 PMC3131480 21677136

[B14] Billman Z. P. Kovacs S. B. Wei B. Kang K. Cissé O. H. Miao E. A. (2024). Caspase-1 activates gasdermin A in non-mammals. eLife 12, RP92362. 10.7554/eLife.92362 38497531 PMC10948149

[B15] Blohberger J. Kunz L. Einwang D. Berg U. Berg D. Ojeda S. R. (2015). Read through acetylcholinesterase (AChE-R) and regulated necrosis: pharmacological targets for the regulation of ovarian functions? Cell. Death Dis. 6 (3), e1685. 10.1038/cddis.2015.51 25766324 PMC4385929

[B16] Brault M. Oberst A. (2017). Controlled detonation: evolution of necroptosis in pathogen defense. Immunol. Cell. Biol. 95 (2), 131–136. 10.1038/icb.2016.117 27909314 PMC6855669

[B17] Britt K. L. Drummond A. E. Cox V. A. Dyson M. Wreford N. G. Jones M. E. (2000). An age-related ovarian phenotype in mice with targeted disruption of the Cyp 19 (aromatase) gene. Endocrinology 141 (7), 2614–2623. 10.1210/endo.141.7.7578 10875266

[B18] Buigues A. Marchante M. de Miguel-Gómez L. Martinez J. Cervelló I. Pellicer A. (2021). Stem cell-secreted factor therapy regenerates the ovarian niche and rescues follicles. Am. J. Obstet. Gynecol. 225 (1), 65.e1–65.e14. 10.1016/j.ajog.2021.01.023 33539826

[B19] Butts S. F. Owen C. Mainigi M. Senapati S. Seifer D. B. Dokras A. (2014). Assisted hatching and intracytoplasmic sperm injection are not associated with improved outcomes in assisted reproduction cycles for diminished ovarian reserve: an analysis of cycles in the United States from 2004 to 2011. Fertil. Steril. 102 (4), 1041–1047. 10.1016/j.fertnstert.2014.06.043 25086790 PMC4184996

[B20] Butts S. F. Ratcliffe S. Dokras A. Seifer D. B. (2013). Diagnosis and treatment of diminished ovarian reserve in assisted reproductive technology cycles of women up to age 40 years: the role of insurance mandates. Fertil. Steril. 99 (2), 382–388. 10.1016/j.fertnstert.2012.09.026 23102859 PMC3561490

[B21] Cai M. Li Q. Cao Y. Huang Y. Yao H. Zhao C. (2024). Quercetin activates autophagy to protect rats ovarian granulosa cells from H2O2-induced aging and injury. Eur. J. Pharmacol. 966, 176339. 10.1016/j.ejphar.2024.176339 38272342

[B22] Cao B. Camden A. J. Parnell L. A. Mysorekar I. U. (2017). Autophagy regulation of physiological and pathological processes in the female reproductive tract. Am. J. Reprod. Immunol. N. Y. N. 1989 77 (5). 10.1111/aji.12650 28194822

[B23] Cao Y. Shen M. Jiang Y. Sun S. C. Liu H. (2018). Melatonin reduces oxidative damage in mouse granulosa cells via restraining JNK-dependent autophagy. Reprod. Camb. Engl. 155 (3), 307–319. 10.1530/REP-18-0002 29363570

[B24] Chen C. Li S. Hu C. Cao W. Fu Q. Li J. (2021). Protective effects of puerarin on premature ovarian failure via regulation of Wnt/β-catenin signaling pathway and oxidative stress. Reprod. Sci. Thousand Oaks Calif. 28 (4), 982–990. 10.1007/s43032-020-00325-0 32996063

[B25] Chen H. Nie P. Li J. Wu Y. Yao B. Yang Y. (2024). Cyclophosphamide induces ovarian granulosa cell ferroptosis via a mechanism associated with HO-1 and ROS-mediated mitochondrial dysfunction. J. Ovarian Res. 17 (1), 107. 10.1186/s13048-024-01434-z 38762721 PMC11102268

[B26] Chen W. Qiukai E. Sun B. Zhang P. Li N. Fei S. (2023a). PARP1-catalyzed PARylation of YY1 mediates endoplasmic reticulum stress in granulosa cells to determine primordial follicle activation. Cell. Death Dis. 14 (8), 524. 10.1038/s41419-023-05984-w 37582914 PMC10427711

[B27] Chen X. Shi C. He M. Xiong S. Xia X. (2023b). Endoplasmic reticulum stress: molecular mechanism and therapeutic targets. Signal Transduct. Target Ther. 8 (1), 352. 10.1038/s41392-023-01570-w 37709773 PMC10502142

[B28] Chen X. Song Q. L. Li Z. H. Ji R. Wang J. Y. Cao M. L. (2023c). Pterostilbene ameliorates oxidative damage and ferroptosis in human ovarian granulosa cells by regulating the Nrf2/HO-1 pathway. Arch. Biochem. Biophys. 738, 109561. 10.1016/j.abb.2023.109561 36898621

[B29] Chen X. Wang Y. Ma N. Tian J. Shao Y. Zhu B. (2020b). Target identification of natural medicine with chemical proteomics approach: probe synthesis, target fishing and protein identification. Signal Transduct. Target Ther. 5 (1), 72. 10.1038/s41392-020-0186-y 32435053 PMC7239890

[B30] Chen X. Yu C. Kang R. Tang D. (2020a). Iron metabolism in ferroptosis. Front. Cell. Dev. Biol. 8, 590226. 10.3389/fcell.2020.590226 33117818 PMC7575751

[B31] Chen Y. Zhao Y. Miao C. Yang L. Wang R. Chen B. (2022). Quercetin alleviates cyclophosphamide-induced premature ovarian insufficiency in mice by reducing mitochondrial oxidative stress and pyroptosis in granulosa cells. J. Ovarian Res. 15 (1), 138. 10.1186/s13048-022-01080-3 36572950 PMC9793602

[B32] Chi Y. N. Hai D. M. Ma L. Cui Y. H. Hu H. T. Liu N. (2023). Protective effects of leonurine hydrochloride on pyroptosis in premature ovarian insufficiency via regulating NLRP3/GSDMD pathway. Int. Immunopharmacol. 114, 109520. 10.1016/j.intimp.2022.109520 36513022

[B33] Choe S. A. Kim M. J. Lee H. J. Kim J. Chang E. M. Kim J. W. (2018). Increased proportion of mature oocytes with sustained-release growth hormone treatment in poor responders: a prospective randomized controlled study. Arch. Gynecol. Obstet. 297 (3), 791–796. 10.1007/s00404-017-4613-4 29264647

[B34] Choi J. Jo M. Lee E. Choi D. (2014). AKT is involved in granulosa cell autophagy regulation via mTOR signaling during rat follicular development and atresia. Reprod. Camb. Engl. 147 (1), 73–80. 10.1530/REP-13-0386 24131573

[B35] Choi J. Y. Jo M. W. Lee E. Y. Yoon B. K. Choi D. S. (2010). The role of autophagy in follicular development and atresia in rat granulosa cells. Fertil. Steril. 93 (8), 2532–2537. 10.1016/j.fertnstert.2009.11.021 20149359

[B36] Coll R. C. Schroder K. Pelegrín P. (2022). NLRP3 and pyroptosis blockers for treating inflammatory diseases. Trends Pharmacol. Sci. 43 (8), 653–668. 10.1016/j.tips.2022.04.003 35513901

[B37] Conforti A. Carbone L. Di Girolamo R. Iorio G. G. Guida M. Campitiello M. R. (2025). Therapeutic management in women with a diminished ovarian reserve: a systematic review and meta-analysis of randomized controlled trials. Fertil. Steril. 123 (3), 457–476. 10.1016/j.fertnstert.2024.09.038 39332623

[B38] Conforti A. Iorio G. G. Di Girolamo R. Rovetto M. Y. Picarelli S. Cariati F. (2024). The impact of resveratrol on the outcome of the *in vitro* fertilization: an exploratory randomized placebo-controlled trial. J. Ovarian Res. 17 (1), 81. 10.1186/s13048-024-01391-7 38622741 PMC11020196

[B39] Cory S. Adams J. M. (2002). The Bcl2 family: regulators of the cellular life-or-death switch. Nat. Rev. Cancer 2 (9), 647–656. 10.1038/nrc883 12209154

[B40] Cui T. Dai X. Guo H. Wang D. Huang B. Pu W. (2024). Molybdenum and cadmium co-induce necroptosis through Th1/Th2 imbalance-mediated endoplasmic reticulum stress in duck ovaries. J. Environ. Sci. China 142, 92–102. 10.1016/j.jes.2023.07.012 38527899

[B41] Cunningham M. A. Zhu Q. Unterman T. G. Hammond J. M. (2003). Follicle-stimulating hormone promotes nuclear exclusion of the forkhead transcription factor FoxO1a via phosphatidylinositol 3-kinase in porcine granulosa cells. Endocrinol. 144 (12), 5585–5594. 10.1210/en.2003-0678 12960025

[B42] Dai W. Xu B. Ding L. Zhang Z. Yang H. He T. (2024). Human umbilical cord mesenchymal stem cells alleviate chemotherapy-induced premature ovarian insufficiency mouse model by suppressing ferritinophagy-mediated ferroptosis in granulosa cells. Free Radic. Biol. Med. 220, 1–14. 10.1016/j.freeradbiomed.2024.04.229 38677487

[B43] Degterev A. Hitomi J. Germscheid M. Ch’en I. L. Korkina O. Teng X. (2008). Identification of RIP1 kinase as a specific cellular target of necrostatins. Nat. Chem. Biol. 4 (5), 313–321. 10.1038/nchembio.83 18408713 PMC5434866

[B44] Dikic I. Elazar Z. (2018). Mechanism and medical implications of mammalian autophagy. Nat. Rev. Mol. Cell. Biol. 19 (6), 349–364. 10.1038/s41580-018-0003-4 29618831

[B45] Ding S. M. Shi L. G. Xing F. Cui S. S. Cheng H. R. Liu Y. (2024). Melatonin protects against mitochondrial dyshomeostasis and ovarian damage caused by chronic unpredictable mild stress through the eIF2α-AFT4 signaling pathway in mice. Reprod. Sci. Thousand Oaks Calif. 31, 3191–3201. 10.1007/s43032-024-01647-z 39060751

[B46] Dixon S. J. Lemberg K. M. Lamprecht M. R. Skouta R. Zaitsev E. M. Gleason C. E. (2012). Ferroptosis: an iron-dependent form of non-apoptotic cell death. Cell 149 (5), 1060–1072. 10.1016/j.cell.2012.03.042 22632970 PMC3367386

[B47] Doll S. Freitas F. P. Shah R. Aldrovandi M. da Silva M. C. Ingold I. (2019). FSP1 is a glutathione-independent ferroptosis suppressor. Nature 575 (7784), 693–698. 10.1038/s41586-019-1707-0 31634899

[B48] Du Y. Bagnjuk K. Lawson M. S. Xu J. Mayerhofer A. (2018). Acetylcholine and necroptosis are players in follicular development in primates. Sci. Rep. 8, 6166. 10.1038/s41598-018-24661-z 29670172 PMC5906600

[B49] Duan H. Yang S. Yang S. Zeng J. Yan Z. Zhang L. (2024). The mechanism of curcumin to protect mouse ovaries from oxidative damage by regulating AMPK/mTOR mediated autophagy. Phytomed. Int. J. Phytother. Phytopharm. 128, 155468. 10.1016/j.phymed.2024.155468 38471315

[B50] Duerrschmidt N. Zabirnyk O. Nowicki M. Ricken A. Hmeidan F. A. Blumenauer V. (2006). Lectin-like oxidized low-density lipoprotein receptor-1-mediated autophagy in human granulosa cells as an alternative of programmed cell death. Endocrinol. 147 (8), 3851–3860. 10.1210/en.2006-0088 16690797

[B51] Eisenberg T. Knauer H. Schauer A. Büttner S. Ruckenstuhl C. Carmona-Gutierrez D. (2009). Induction of autophagy by spermidine promotes longevity. Nat. Cell. Biol. 11 (11), 1305–1314. 10.1038/ncb1975 19801973

[B53] Eslami M. Esfandyari S. Aghahosseini M. Rashidi Z. Hosseinishental S. H. Brenjian S. (2021). Astaxanthin protects human granulosa cells against oxidative stress through activation of NRF2/ARE pathway and its downstream phase II enzymes. Cell. J. 23 (3), 319–328. 10.22074/cellj.2021.7222 34308575 PMC8286460

[B54] Espino J. Macedo M. Lozano G. Ortiz Á. Rodríguez C. Rodríguez A. B. (2019). Impact of melatonin supplementation in women with unexplained infertility undergoing fertility treatment. Antioxid. Basel Switz. 8 (9), 338. 10.3390/antiox8090338 PMC6769719 31450726

[B55] Fabová Z. Kislíková Z. Loncová B. Bauer M. Harrath A. H. Sirotkin A. V. (2023). MicroRNA miR-152 can support ovarian granulosa cell functions and modify apigenin actions. Domest. Anim. Endocrinol., 84–85. 10.1016/j.domaniend.2023.106805 37354873

[B56] Fàbregues F. Ferreri J. Méndez M. Calafell J. M. Otero J. Farré R. (2020). *In vitro* follicular activation and stem cell therapy as a novel treatment strategies in diminished ovarian reserve and primary ovarian insufficiency. Front. Endocrinol. 11, 617704. 10.3389/fendo.2020.617704 PMC7943854 33716954

[B57] Faddy M. J. Gosden R. G. Gougeon A. Richardson S. J. Nelson J. F. (1992). Accelerated disappearance of ovarian follicles in mid-life: implications for forecasting menopause. Hum. Reprod. Oxf. Engl. 7 (10), 1342–1346. 10.1093/oxfordjournals.humrep.a137570 1291557

[B58] Faghani M. Saedi S. Khanaki K. Mohammadghasemi F. (2022). Ginseng alleviates folliculogenesis disorders via induction of cell proliferation and downregulation of apoptotic markers in nicotine-treated mice. J. Ovarian Res. 15 (1), 14. 10.1186/s13048-022-00945-x 35067219 PMC8785492

[B59] Fan P. Jordan V. C. (2022). PERK, beyond an unfolded protein response sensor in estrogen-induced apoptosis in endocrine-resistant breast cancer. Mol. Cancer Res. 20 (2), 193–201. 10.1158/1541-7786.MCR-21-0702 34728551 PMC8816868

[B60] Fernando S. Wallace E. M. Vollenhoven B. Lolatgis N. Hope N. Wong M. (2018). Melatonin in assisted reproductive technology: a pilot double-blind randomized placebo-controlled clinical trial. Front. Endocrinol. 9, 545. 10.3389/fendo.2018.00545 PMC6157331 30283403

[B61] Frank D. Vince J. E. (2019). Pyroptosis versus necroptosis: similarities, differences, and crosstalk. Cell. Death Differ. 26 (1), 99–114. 10.1038/s41418-018-0212-6 30341423 PMC6294779

[B62] Galluzzi L. Vitale I. Aaronson S. A. Abrams J. M. Adam D. Agostinis P. (2018). Molecular mechanisms of cell death: recommendations of the nomenclature committee on cell death 2018. Cell. Death Differ. 25 (3), 486–541. 10.1038/s41418-017-0012-4 29362479 PMC5864239

[B63] Galluzzi L. Vitale I. Abrams J. M. Alnemri E. S. Baehrecke E. H. Blagosklonny M. V. (2012). Molecular definitions of cell death subroutines: recommendations of the Nomenclature Committee on Cell Death 2012. Cell. Death Differ. 19 (1), 107–120. 10.1038/cdd.2011.96 21760595 PMC3252826

[B64] Gannon A. M. Stämpfli M. R. Foster W. G. (2012). Cigarette smoke exposure leads to follicle loss via an alternative ovarian cell death pathway in a mouse model. Toxicol. Sci. 125 (1), 274–284. 10.1093/toxsci/kfr279 22003194

[B65] Gannon A. M. Stämpfli M. R. Foster W. G. (2013). Cigarette smoke exposure elicits increased autophagy and dysregulation of mitochondrial dynamics in murine granulosa cells. Biol. Reprod. 88 (3), 63. 10.1095/biolreprod.112.106617 23325812

[B66] Gawriluk T. R. Hale A. N. Flaws J. A. Dillon C. P. Green D. R. Rucker E. B. (2011). Autophagy is a cell survival program for female germ cells in the murine ovary. Reprod. Camb. Engl. 141 (6), 759–765. 10.1530/REP-10-0489 21464117

[B67] Glister C. Tannetta D. S. Groome N. P. Knight P. G. (2001). Interactions between follicle-stimulating hormone and growth factors in modulating secretion of steroids and inhibin-related peptides by nonluteinized bovine granulosa cells. Biol. Reprod. 65 (4), 1020–1028. 10.1095/biolreprod65.4.1020 11566722

[B68] Gonfloni S. Di Tella L. Caldarola S. Cannata S. M. Klinger F. G. Di Bartolomeo C. (2009). Inhibition of the c-Abl-TAp63 pathway protects mouse oocytes from chemotherapy-induced death. Nat. Med. 15 (10), 1179–1185. 10.1038/nm.2033 19783996

[B69] Gonzalvez F. Ashkenazi A. (2010). New insights into apoptosis signaling by Apo2L/TRAIL. Oncogene 29 (34), 4752–4765. 10.1038/onc.2010.221 20531300

[B70] Gross P. (2024). Mitophagy protects against ferroptosis. Nat. Cell. Biol. 26 (9), 1374. 10.1038/s41556-024-01507-7 39266699

[B71] Guo B. Zhang S. Wang S. Zhang H. Fang J. Kang N. (2023). Decreased HAT1 expression in granulosa cells disturbs oocyte meiosis during mouse ovarian aging. Reprod. Biol. Endocrinol. 21 (1), 103. 10.1186/s12958-023-01147-w 37907924 PMC10617186

[B72] Gupta V. K. Scheunemann L. Eisenberg T. Mertel S. Bhukel A. Koemans T. S. (2013). Restoring polyamines protects from age-induced memory impairment in an autophagy-dependent manner. Nat. Neurosci. 16 (10), 1453–1460. 10.1038/nn.3512 23995066

[B73] Hakuno N. Koji T. Yano T. Kobayashi N. Tsutsumi O. Taketani Y. (1996). Fas/APO-1/CD95 system as a mediator of granulosa cell apoptosis in ovarian follicle atresia. Endocrinol. 137 (5), 1938–1948. 10.1210/endo.137.5.8612534 8612534

[B74] Hansen K. R. Knowlton N. S. Thyer A. C. Charleston J. S. Soules M. R. Klein N. A. (2008). A new model of reproductive aging: the decline in ovarian non-growing follicle number from birth to menopause. Hum. Reprod. Oxf. Engl. 23 (3), 699–708. 10.1093/humrep/dem408 18192670

[B75] Haraguchi H. Hirota Y. Saito-Fujita T. Tanaka T. Shimizu-Hirota R. Harada M. (2019). Mdm2-p53-SF1 pathway in ovarian granulosa cells directs ovulation and fertilization by conditioning oocyte quality. FASEB J. 33 (2), 2610–2620. 10.1096/fj.201801401R 30260703

[B76] Härtl R. Gleinich A. Zimmermann M. (2011). Dramatic increase in readthrough acetylcholinesterase in a cellular model of oxidative stress. J. Neurochem. 116 (6), 1088–1096. 10.1111/j.1471-4159.2010.07164.x 21198638

[B77] Hojo T. Siemieniuch M. J. Lukasik K. Piotrowska-Tomala K. K. Jonczyk A. W. Okuda K. (2016). Programmed necrosis - a new mechanism of steroidogenic luteal cell death and elimination during luteolysis in cows. Sci. Rep. 6, 38211. 10.1038/srep38211 27901113 PMC5128806

[B78] Holler N. Zaru R. Micheau O. Thome M. Attinger A. Valitutti S. (2000). Fas triggers an alternative, caspase-8-independent cell death pathway using the kinase RIP as effector molecule. Nat. Immunol. 1 (6), 489–495. 10.1038/82732 11101870

[B79] Hou J. Lei Z. Cui L. Hou Y. Yang L. (2021). Polystyrene microplastics lead to pyroptosis and apoptosis of ovarian granulosa cells via NLRP3/Caspase-1 signaling pathway in rats. Ecotoxicol. Environ. Saf. 212, 112012. 10.1016/j.ecoenv.2021.112012 33550074

[B80] Høyer-Hansen M. Bastholm L. Szyniarowski P. Campanella M. Szabadkai G. Farkas T. (2007). Control of macroautophagy by calcium, calmodulin-dependent kinase kinase-beta, and Bcl-2. Mol. Cell. 25 (2), 193–205. 10.1016/j.molcel.2006.12.009 17244528

[B81] Hsu S. Y. Lai R. J. Finegold M. Hsueh A. J. (1996). Targeted overexpression of Bcl-2 in ovaries of transgenic mice leads to decreased follicle apoptosis, enhanced folliculogenesis, and increased germ cell tumorigenesis. Endocrinol. 137 (11), 4837–4843. 10.1210/endo.137.11.8895354 8895354

[B82] Hu B. Zheng X. Zhang W. (2024). Resveratrol-βcd inhibited premature ovarian insufficiency progression by regulating granulosa cell autophagy. J. Ovarian Res. 17, 18. 10.1186/s13048-024-01344-0 38221630 PMC10789063

[B83] Huang C. Zhao S. Yang Y. Guo T. Ke H. Mi X. (2023). TP63 gain-of-function mutations cause premature ovarian insufficiency by inducing oocyte apoptosis. J. Clin. Invest. 133 (5), e162315. 10.1172/JCI162315 36856110 PMC9974095

[B84] Huang P. Zhou Y. Tang W. Ren C. Jiang A. Wang X. (2022). Long-term treatment of Nicotinamide mononucleotide improved age-related diminished ovary reserve through enhancing the mitophagy level of granulosa cells in mice. J. Nutr. Biochem. 101, 108911. 10.1016/j.jnutbio.2021.108911 34801690

[B85] Huang S. Xing Y. Liu Y. (2019a). Emerging roles for the ER stress sensor IRE1α in metabolic regulation and disease. J. Biol. Chem. 294 (49), 18726–18741. 10.1074/jbc.REV119.007036 31666338 PMC6901316

[B86] Huang W. Cao Y. Shi L. (2019b). Effects of FSHR polymorphisms on premature ovarian insufficiency in human beings: a meta-analysis. Reprod. Biol. Endocrinol. 17 (1), 80. 10.1186/s12958-019-0528-1 31629411 PMC6800985

[B87] Humaidan P. Chin W. Rogoff D. D’Hooghe T. Longobardi S. Hubbard J. (2017). Efficacy and safety of follitropin alfa/lutropin alfa in ART: a randomized controlled trial in poor ovarian responders. Hum. Reprod. Oxf. Engl. 32 (3), 544–555. 10.1093/humrep/dew360 PMC5850777 28137754

[B88] Ibrahim M. A. Albahlol I. A. Wani F. A. Abd-Eltawab Tammam A. Kelleni M. T. Sayeed M. U. (2021). Resveratrol protects against cisplatin-induced ovarian and uterine toxicity in female rats by attenuating oxidative stress, inflammation and apoptosis. Chem. Biol. Interact. 338, 109402. 10.1016/j.cbi.2021.109402 33587916

[B89] Imai S. Guarente L. (2014). NAD+ and sirtuins in aging and disease. Trends Cell. Biol. 24 (8), 464–471. 10.1016/j.tcb.2014.04.002 24786309 PMC4112140

[B90] Inoue N. Matsuda F. Goto Y. Manabe N. (2011). Role of cell-death ligand-receptor system of granulosa cells in selective follicular atresia in porcine ovary. J. Reprod. Dev. 57 (2), 169–175. 10.1262/jrd.10-198e 21551974

[B91] Inoue N. Matsuda-Minehata F. Goto Y. Sakamaki K. Manabe N. (2007). Molecular characteristics of porcine Fas-associated death domain (FADD) and procaspase-8. J. Reprod. Dev. 53 (2), 427–436. 10.1262/jrd.18136 17179649

[B92] Itakura E. Kishi-Itakura C. Mizushima N. (2012). The hairpin-type tail-anchored SNARE syntaxin 17 targets to autophagosomes for fusion with endosomes/lysosomes. Cell 151 (6), 1256–1269. 10.1016/j.cell.2012.11.001 23217709

[B93] Jiang D. Guo Y. Niu C. Long S. Jiang Y. Wang Z. (2023). Exploration of the antioxidant effect of spermidine on the ovary and screening and identification of differentially expressed proteins. Int. J. Mol. Sci. 24 (6), 5793. 10.3390/ijms24065793 36982867 PMC10051986

[B94] Jiang J. Y. Cheung C. K. M. Wang Y. Tsang B. K. (2003). Regulation of cell death and cell survival gene expression during ovarian follicular development and atresia. Front. Biosci. J. Virtual Libr. 8, d222–d237. 10.2741/949 12456353

[B95] Jiang X. Stockwell B. R. Conrad M. (2021). Ferroptosis: mechanisms, biology, and role in disease. Nat. Rev. Mol. Cell. Biol. 22 (4), 266–282. 10.1038/s41580-020-00324-8 33495651 PMC8142022

[B96] Jin X. Wang K. Wang L. Liu W. Zhang C. Qiu Y. (2022). RAB7 activity is required for the regulation of mitophagy in oocyte meiosis and oocyte quality control during ovarian aging. Autophagy 18 (3), 643–660. 10.1080/15548627.2021.1946739 34229552 PMC9037413

[B97] Jo J. W. Lee J. R. Jee B. C. Suh C. S. Kim S. H. (2015). Exposing mouse oocytes to Necrostatin 1 during *in vitro* maturation improves maturation, survival after vitrification, mitochondrial preservation, and developmental competence. Reprod. Sci. 22 (5), 615–625. 10.1177/1933719114556482 25394642 PMC4519766

[B98] Joza N. Susin S. A. Daugas E. Stanford W. L. Cho S. K. Li C. Y. (2001). Essential role of the mitochondrial apoptosis-inducing factor in programmed cell death. Nature 410 (6828), 549–554. 10.1038/35069004 11279485

[B99] Kara M. Aydin T. Aran T. Turktekin N. Ozdemir B. (2014). Does dehydroepiandrosterone supplementation really affect IVF-ICSI outcome in women with poor ovarian reserve? Eur. J. Obstet. Gynecol. Reprod. Biol. 173, 63–65. 10.1016/j.ejogrb.2013.11.008 24331115

[B100] Kerr J. B. Hutt K. J. Michalak E. M. Cook M. Vandenberg C. J. Liew S. H. (2012). DNA damage-induced primordial follicle oocyte apoptosis and loss of fertility require TAp63-mediated induction of Puma and Noxa. Mol. Cell. 48 (3), 343–352. 10.1016/j.molcel.2012.08.017 23000175 PMC3496022

[B101] Khallaf W. A. I. Sharata E. E. Attya M. E. Abo-Youssef A. M. Hemeida R. A. M. (2023). LCZ696 (sacubitril/valsartan) mitigates cyclophosphamide-induced premature ovarian failure in rats; the role of TLR4/NF-κB/NLRP3/Caspase-1 signaling pathway. Life Sci. 326, 121789. 10.1016/j.lfs.2023.121789 37201697

[B102] Khan S. A. Reed L. Schoolcraft W. B. Yuan Y. Krisher R. L. (2023). Control of mitochondrial integrity influences oocyte quality during reproductive aging. Mol. Hum. Reprod. 29 (9), gaad028. 10.1093/molehr/gaad028 37594790

[B103] Klionsky D. J. Petroni G. Amaravadi R. K. Baehrecke E. H. Ballabio A. Boya P. (2021). Autophagy in major human diseases. EMBO J. 40 (19), e108863. 10.15252/embj.2021108863 34459017 PMC8488577

[B104] Kotb M. M. M. Hassan A. M. A. AwadAllah A. M. A. (2016). Does dehydroepiandrosterone improve pregnancy rate in women undergoing IVF/ICSI with expected poor ovarian response according to the Bologna criteria? A randomized controlled trial. Eur. J. Obstet. Gynecol. Reprod. Biol. 200, 11–15. 10.1016/j.ejogrb.2016.02.009 26963897

[B105] Kovacs S. B. Miao E. A. (2017). Gasdermins: effectors of pyroptosis. Trends Cell. Biol. 27 (9), 673–684. 10.1016/j.tcb.2017.05.005 28619472 PMC5565696

[B106] Kraft V. A. N. Bezjian C. T. Pfeiffer S. Ringelstetter L. Müller C. Zandkarimi F. (2020). GTP cyclohydrolase 1/tetrahydrobiopterin counteract ferroptosis through lipid remodeling. ACS Cent. Sci. 6 (1), 41–53. 10.1021/acscentsci.9b01063 31989025 PMC6978838

[B107] Krysko D. V. Diez-Fraile A. Criel G. Svistunov A. A. Vandenabeele P. D’Herde K. (2008). Life and death of female gametes during oogenesis and folliculogenesis. Apoptosis Int. J. Program Cell. Death 13 (9), 1065–1087. 10.1007/s10495-008-0238-1 18622770

[B108] Kugu K. Ratts V. S. Piquette G. N. Tilly K. I. Tao X. J. Martimbeau S. (1998). Analysis of apoptosis and expression of bcl-2 gene family members in the human and baboon ovary. Cell. Death Differ. 5 (1), 67–76. 10.1038/sj.cdd.4400316 10200447

[B109] Kulikov A. V. Shilov E. S. Mufazalov I. A. Gogvadze V. Nedospasov S. A. Zhivotovsky B. (2012). Cytochrome c: the Achilles’ heel in apoptosis. Cell. Mol. Life Sci. 69 (11), 1787–1797. 10.1007/s00018-011-0895-z 22179840 PMC11114681

[B110] Lai Q. Xiang W. Li Q. Zhang H. Li Y. Zhu G. (2018). Oxidative stress in granulosa cells contributes to poor oocyte quality and IVF-ET outcomes in women with polycystic ovary syndrome. Front. Med. 12 (5), 518–524. 10.1007/s11684-017-0575-y 29260383

[B111] Legembre P. Moreau P. Daburon S. Moreau J. F. Taupin J. L. (2002). Potentiation of Fas-mediated apoptosis by an engineered glycosylphosphatidylinositol-linked Fas. Cell. Death Differ. 9 (3), 329–339. 10.1038/sj.cdd.4400960 11859415

[B112] Li F. Zhu F. Wang S. Hu H. Zhang D. He Z. (2024a). Icariin alleviates cisplatin-induced premature ovarian failure by inhibiting ferroptosis through activation of the Nrf2/ARE pathway. Sci. Rep. 14 (1), 17318. 10.1038/s41598-024-67557-x 39068256 PMC11283570

[B113] Li H. Jing Y. Qu X. Yang J. Pan P. Liu X. (2023a). The activation of reticulophagy by ER stress through the ATF4-MAP1LC3A-CCPG1 pathway in ovarian granulosa cells is linked to apoptosis and necroptosis. Int. J. Mol. Sci. 24 (3), 2749. 10.3390/ijms24032749 36769070 PMC9917250

[B114] Li H. Wang X. Mu H. Mei Q. Liu Y. Min Z. (2022a). Mir-484 contributes to diminished ovarian reserve by regulating granulosa cell function via YAP1-mediated mitochondrial function and apoptosis. Int. J. Biol. Sci. 18 (3), 1008–1021. 10.7150/ijbs.68028 35173533 PMC8771835

[B115] Li J. Cao F. Yin H. L. Huang Z. J. Lin Z. T. Mao N. (2020a). Ferroptosis: past, present and future. Cell. Death Dis. 11 (2), 88. 10.1038/s41419-020-2298-2 32015325 PMC6997353

[B116] Li P. Dou Q. Zhang D. Xiang Y. Tan L. (2024b). Melatonin regulates autophagy in granulosa cells from patients with premature ovarian insufficiency via activating Foxo3a. Aging 16 (1), 844–856. 10.18632/aging.205424 38206302 PMC10817365

[B117] Li T. Liu J. Liu K. Wang Q. Cao J. Xiao P. (2023b). Alpha-ketoglutarate ameliorates induced premature ovarian insufficiency in rats by inhibiting apoptosis and upregulating glycolysis. Reprod. Biomed. Online 46 (4), 673–685. 10.1016/j.rbmo.2023.01.005 36894359

[B118] Li X. Li X. Deng L. (2022b). Chrysin reduces inflammation and oxidative stress and improves ovarian function in D-gal-induced premature ovarian failure. Bioengineered 13 (4), 8291–8301. 10.1080/21655979.2021.2005991 35311454 PMC9161991

[B119] Li X. Qi J. Zhu Q. He Y. Wang Y. Lu Y. (2019). The role of androgen in autophagy of granulosa cells from PCOS. Gynecol. Endocrinol. Off. J. Int. Soc. Gynecol. Endocrinol. 35 (8), 669–672. 10.1080/09513590.2018.1540567 31056990

[B120] Li X. Ye H. Su T. Hu C. Huang Y. Fu X. (2023c). Immunity and reproduction protective effects of Chitosan Oligosaccharides in Cyclophosphamide/Busulfan-induced premature ovarian failure model mice. Front. Immunol. 14, 1185921. 10.3389/fimmu.2023.1185921 37228612 PMC10203494

[B121] Li Y. Qiu W. Zhang Z. Han X. Bu G. Meng F. (2020b). Oral oyster polypeptides protect ovary against d-galactose-induced premature ovarian failure in C57BL/6 mice. J. Sci. Food Agric. 100 (1), 92–101. 10.1002/jsfa.9997 31435952

[B122] Liang D. Minikes A. M. Jiang X. (2022). Ferroptosis at the intersection of lipid metabolism and cellular signaling. Mol. Cell. 82 (12), 2215–2227. 10.1016/j.molcel.2022.03.022 35390277 PMC9233073

[B123] Liang S. Wang F. Bao C. Han J. Guo Y. Liu F. (2020). BAG2 ameliorates endoplasmic reticulum stress-induced cell apoptosis in Mycobacterium tuberculosis-infected macrophages through selective autophagy. Autophagy 16 (8), 1453–1467. 10.1080/15548627.2019.1687214 31711362 PMC7469592

[B124] Liang Y. Shi Y. Guo R. Xu C. Fu M. Shen J. (2024). Wine- and stir-frying processing of Cuscutae Semen enhance its ability to alleviate oxidative stress and apoptosis via the Keap 1-Nrf2/HO-1 and PI3K/AKT pathways in H2O2-challenged KGN human granulosa cell line. BMC Complement. Med. Ther. 24, 189. 10.1186/s12906-024-04491-5 38750475 PMC11094956

[B125] Liang Y. Xu M. L. Gao X. Wang Y. Zhang L. N. Li Y. C. (2023). Resveratrol improves ovarian state by inhibiting apoptosis of granulosa cells. Gynecol. Endocrinol. 39 (1), 2181652. 10.1080/09513590.2023.2181652 36824010

[B126] Lin P. H. Su W. P. Li C. J. Lin L. T. Sheu J. J. C. Wen Z. H. (2023). Investigating the role of ferroptosis-related genes in ovarian aging and the potential for nutritional intervention. Nutrients 15 (11), 2461. 10.3390/nu15112461 37299424 PMC10255416

[B127] Liu H. Jiang C. La B. Cao M. Ning S. Zhou J. (2021a). Human amnion-derived mesenchymal stem cells improved the reproductive function of age-related diminished ovarian reserve in mice through Ampk/FoxO3a signaling pathway. Stem Cell. Res. Ther. 12 (1), 317. 10.1186/s13287-021-02382-x 34078462 PMC8173966

[B128] Liu H. Xu X. Han T. Yan L. Cheng L. Qin Y. (2017). A novel homozygous mutation in the FSHR gene is causative for primary ovarian insufficiency. Fertil. Steril. 108 (6), 1050–1055. 10.1016/j.fertnstert.2017.09.010 29157895

[B129] Liu K. Wu Y. Yang W. Li T. Wang Z. Xiao S. (2024b). α-Ketoglutarate improves ovarian reserve function in primary ovarian insufficiency by inhibiting NLRP3-mediated pyroptosis of granulosa cells. Mol. Nutr. Food Res. 68 (5), e2300784. 10.1002/mnfr.202300784 38314939

[B130] Liu Q. Z. Han H. Fang X. R. Wang L. Y. Zhao D. Yin M. Z. (2024a). Berberine alleviates ovarian tissue damage in mice with hepatolenticular degeneration by suppressing ferroptosis and endoplasmic reticulum stress. J. Integr. Med. 22 (4), 493–502. 10.1016/j.joim.2024.05.003 38853116

[B131] Liu W. Chen Q. Liu Z. Weng Z. Nguyen T. N. Feng J. (2021b). Zihuai recipe alleviates cyclophosphamide-induced diminished ovarian reserve via suppressing PI3K/AKT-mediated apoptosis. J. Ethnopharmacol. 277, 113789. 10.1016/j.jep.2021.113789 33422655

[B132] Liu W. Wang X. Chen Y. Zhang H. Chen J. Zhang J. (2022a). A combination containing natural extracts of clove, Sophora flower bud, and yam improves fertility in aged female mice via multiple mechanisms. Front. Endocrinol. 13, 945690. 10.3389/fendo.2022.945690 PMC9724743 36483000

[B133] Liu X. Lin X. Mi Y. Li J. Zhang C. (2018a). Grape seed proanthocyanidin extract prevents ovarian aging by inhibiting oxidative stress in the hens. Oxid. Med. Cell. Longev. 2018, 9390810. 10.1155/2018/9390810 29541349 PMC5818927

[B134] Liu Y. Duan C. Chen H. Wang C. Liu X. Qiu M. (2018b). Inhibition of COX-2/mPGES-1 and 5-LOX in macrophages by leonurine ameliorates monosodium urate crystal-induced inflammation. Toxicol. Appl. Pharmacol. 351, 1–11. 10.1016/j.taap.2018.05.010 29763636

[B135] Liu Y. Fang Y. Wei J. Zhang C. Wu D. Li Y. (2022b). Melatonin protects against primary ovarian insufficiency by activating the PI3K/Akt/mTOR pathway and inhibiting autophagy. Ann. Clin. Lab. Sci. 52 (6), 895–903. 36564068

[B136] Liu Y. Gu W. (2022). p53 in ferroptosis regulation: the new weapon for the old guardian. Cell. Death Differ. 29 (5), 895–910. 10.1038/s41418-022-00943-y 35087226 PMC9091200

[B137] Lliberos C. Liew S. H. Mansell A. Hutt K. J. (2021b). The inflammasome contributes to depletion of the ovarian reserve during aging in mice. Front. Cell. Dev. Biol. 8, 628473. 10.3389/fcell.2020.628473 33644037 PMC7905095

[B138] Lliberos C. Liew S. H. Zareie P. La Gruta N. L. Mansell A. Hutt K. (2021a). Evaluation of inflammation and follicle depletion during ovarian ageing in mice. Sci. Rep. 11, 278. 10.1038/s41598-020-79488-4 33432051 PMC7801638

[B139] Lu Y. Li Z. Zhang S. Zhang T. Liu Y. Zhang L. (2023). Cellular mitophagy: mechanism, roles in diseases and small molecule pharmacological regulation. Theranostics 13 (2), 736–766. 10.7150/thno.79876 36632220 PMC9830443

[B140] Ma Q. Shen M. Wu J. Ye C. Tan Y. (2024). Mechanism research of DHEA treatment improving diminished ovarian reserve by attenuating the AMPK-SIRT1 signaling and mitophagy. Reprod. Sci. Thousand Oaks Calif. 31 (7), 2059–2072. 10.1007/s43032-024-01473-3 38453773

[B141] Madeo F. Carmona-Gutierrez D. Hofer S. J. Kroemer G. (2019). Caloric restriction mimetics against age-associated disease: targets, mechanisms, and therapeutic potential. Cell. Metab. 29 (3), 592–610. 10.1016/j.cmet.2019.01.018 30840912

[B142] Madeo F. Eisenberg T. Pietrocola F. Kroemer G. (2018). Spermidine in health and disease. Science 359 (6374), eaan2788. 10.1126/science.aan2788 29371440

[B143] Mantawy E. M. Said R. S. Abdel-Aziz A. K. (2019). Mechanistic approach of the inhibitory effect of chrysin on inflammatory and apoptotic events implicated in radiation-induced premature ovarian failure: emphasis on TGF-β/MAPKs signaling pathway. Biomed. Pharmacother. 109, 293–303. 10.1016/j.biopha.2018.10.092 30396087

[B144] Marcinkiewicz J. L. Balchak S. K. Morrison L. J. (2002). The involvement of tumor necrosis factor-alpha (TNF) as an intraovarian regulator of oocyte apoptosis in the neonatal rat. Front. Biosci. J. Virtual Libr. 7, d1997–d2005. 10.2741/A894 12161343

[B145] Marcozzi S. Rossi V. Salustri A. De Felici M. Klinger F. G. (2018). Programmed cell death in the human ovary. Minerva Ginecol. 70 (5), 549–560. 10.23736/S0026-4784.18.04274-0 29999289

[B146] Mariño G. Niso-Santano M. Baehrecke E. H. Kroemer G. (2014). Self-consumption: the interplay of autophagy and apoptosis. Nat. Rev. Mol. Cell. Biol. 15 (2), 81–94. 10.1038/nrm3735 24401948 PMC3970201

[B147] Matsuda F. Inoue N. Goto Y. Maeda A. Cheng Y. Sakamaki K. (2008). cFLIP regulates death receptor-mediated apoptosis in an ovarian granulosa cell line by inhibiting procaspase-8 cleavage. J. Reprod. Dev. 54 (5), 314–320. 10.1262/jrd.20051 18603835

[B148] Matsuda-Minehata F. Inoue N. Goto Y. Manabe N. (2006). The regulation of ovarian granulosa cell death by pro- and anti-apoptotic molecules. J. Reprod. Dev. 52 (6), 695–705. 10.1262/jrd.18069 16926526

[B149] Mayerhofer A. Fritz S. (2002). Ovarian acetylcholine and muscarinic receptors: hints of a novel intrinsic ovarian regulatory system. Microsc. Res. Tech. 59 (6), 503–508. 10.1002/jemt.10228 12467026

[B150] McEvoy M. J. Sinderewicz E. Creedon L. McAfee M. Jonczyk A. W. Piotrowska-Tomala K. K. (2021). Death processes in bovine theca and granulosa cells modelled and analysed using a systems biology approach. Int. J. Mol. Sci. 22 (9), 4888. 10.3390/ijms22094888 34063056 PMC8125194

[B151] Meng L. Jan S. Z. Hamer G. van Pelt A. M. van der Stelt I. Keijer J. (2018). Preantral follicular atresia occurs mainly through autophagy, while antral follicles degenerate mostly through apoptosis. Biol. Reprod. 99 (4), 853–863. 10.1093/biolre/ioy116 29767707

[B152] Meng Y. Lyu Y. Gong J. Zou Y. Jiang X. Xiao M. (2024). Therapeutic effects of curculigoside on cyclophosphamide-induced premature ovarian failure in mice. Climacteric J. Int. Menopause Soc. 27 (4), 421–432. 10.1080/13697137.2024.2354742 38990052

[B153] Mizushima N. Komatsu M. (2011). Autophagy: renovation of cells and tissues. Cell 147 (4), 728–741. 10.1016/j.cell.2011.10.026 22078875

[B154] Mobasher M. A. Hassen M. T. Ebiya R. A. Alturki N. A. Alzamami A. Mohamed H. K. (2022). Ameliorative effect of Citrus lemon peel extract and resveratrol on premature ovarian failure rat model: role of iNOS/caspase-3 pathway. Molecules 28 (1), 122. 10.3390/molecules28010122 36615313 PMC9822383

[B155] Monniaux D. Pisselet C. (1992). Control of proliferation and differentiation of ovine granulosa cells by insulin-like growth factor-I and follicle-stimulating hormone *in vitro* . Biol. Reprod. 46 (1), 109–119. 10.1095/biolreprod46.1.109 1547308

[B156] Murao A. Aziz M. Wang H. Brenner M. Wang P. (2021). Release mechanisms of major DAMPs. Apoptosis Int. J. Program Cell. Death 26 (3-4), 152–162. 10.1007/s10495-021-01663-3 PMC8016797 33713214

[B157] Musters A. M. van Wely M. Mastenbroek S. Kaaijk E. M. Repping S. van der Veen F. (2012). The effect of recombinant LH on embryo quality: a randomized controlled trial in women with poor ovarian reserve. Hum. Reprod. Oxf. Engl. 27 (1), 244–250. 10.1093/humrep/der371 22095792

[B158] Nailwal H. Chan F. K. M. (2019). Necroptosis in anti-viral inflammation. Cell. Death Differ. 26 (1), 4–13. 10.1038/s41418-018-0172-x 30050058 PMC6294789

[B159] Namvar Z. Ramezani Tehrani F. Shahsavani A. Khodagholi F. Hashemi S. S. Binayi F. (2024). Reduction of ovarian reserves and activation of necroptosis to *in vivo* air pollution exposures. Int. J. Environ. Health Res. 34 (4), 2052–2066. 10.1080/09603123.2023.2210109 37204020

[B160] Narkwichean A. Maalouf W. Baumgarten M. Polanski L. Raine-Fenning N. Campbell B. (2017). Efficacy of Dehydroepiandrosterone (DHEA) to overcome the effect of ovarian ageing (DITTO): a proof of principle double blinded randomized placebo controlled trial. Eur. J. Obstet. Gynecol. Reprod. Biol. 218, 39–48. 10.1016/j.ejogrb.2017.09.006 28934714

[B161] Navarro-Pando J. M. Alcocer-Gómez E. Castejón-Vega B. Navarro-Villarán E. Condés-Hervás M. Mundi-Roldan M. (2021). Inhibition of the NLRP3 inflammasome prevents ovarian aging. Sci. Adv. 7 (1), eabc7409. 10.1126/sciadv.abc7409 33523841 PMC7775749

[B162] Newton K. Manning G. (2016). Necroptosis and inflammation. Annu. Rev. Biochem. 85, 743–763. 10.1146/annurev-biochem-060815-014830 26865533

[B163] Newton K. Strasser A. Kayagaki N. Dixit V. M. (2024). Cell death. Cell. 187 (2), 235–256. 10.1016/j.cell.2023.11.044 38242081

[B164] Niu C. Jiang D. Guo Y. Wang Z. Sun Q. Wang X. (2023). Spermidine suppresses oxidative stress and ferroptosis by Nrf2/HO-1/GPX4 and Akt/FHC/ACSL4 pathway to alleviate ovarian damage. Life Sci. 332, 122109. 10.1016/j.lfs.2023.122109 37741320

[B165] Nozaki K. Li L. Miao E. A. (2022). Innate sensors trigger regulated cell death to combat intracellular infection. Annu. Rev. Immunol. 40, 469–498. 10.1146/annurev-immunol-101320-011235 35138947 PMC9614550

[B166] Oakes S. A. Papa F. R. (2015). The role of endoplasmic reticulum stress in human pathology. Annu. Rev. Pathol. 10, 173–194. 10.1146/annurev-pathol-012513-104649 25387057 PMC5568783

[B167] Oktay K. H. Marin L. Petrikovsky B. Terrani M. Babayev S. N. (2021). Delaying reproductive aging by ovarian tissue cryopreservation and transplantation: is it prime time? Trends Mol. Med. 27 (8), 753–761. 10.1016/j.molmed.2021.01.005 33549473 PMC8427891

[B168] Orning P. Weng D. Starheim K. Ratner D. Best Z. Lee B. (2018). Pathogen blockade of TAK1 triggers caspase-8-dependent cleavage of gasdermin D and cell death. Science 362 (6418), 1064–1069. 10.1126/science.aau2818 30361383 PMC6522129

[B169] Pan Y. Gan M. Wu S. He Y. Feng J. Jing Y. (2024). tRF-Gly-GCC in atretic follicles promotes ferroptosis in granulosa cells by down-regulating MAPK1. Int. J. Mol. Sci. 25 (16), 9061. 10.3390/ijms25169061 39201747 PMC11354299

[B170] Park D. Jeong H. Lee M. N. Koh A. Kwon O. Yang Y. R. (2016). Resveratrol induces autophagy by directly inhibiting mTOR through ATP competition. Sci. Rep. 6, 21772. 10.1038/srep21772 26902888 PMC4763238

[B171] Park E. Chung S. W. (2019). ROS-mediated autophagy increases intracellular iron levels and ferroptosis by ferritin and transferrin receptor regulation. Cell. Death Dis. 10 (11), 822. 10.1038/s41419-019-2064-5 31659150 PMC6817894

[B172] Park Y. Maizels E. T. Feiger Z. J. Alam H. Peters C. A. Woodruff T. K. (2005). Induction of cyclin D2 in rat granulosa cells requires FSH-dependent relief from FOXO1 repression coupled with positive signals from Smad. J. Biol. Chem. 280 (10), 9135–9148. 10.1074/jbc.M409486200 15613482 PMC1564190

[B173] Perez G. I. Robles R. Knudson C. M. Flaws J. A. Korsmeyer S. J. Tilly J. L. (1999). Prolongation of ovarian lifespan into advanced chronological age by Bax-deficiency. Nat. Genet. 21 (2), 200–203. 10.1038/5985 9988273

[B174] Picca A. Faitg J. Auwerx J. Ferrucci L. D’Amico D. (2023). Mitophagy in human health, ageing and disease. Nat. Metab. 5 (12), 2047–2061. 10.1038/s42255-023-00930-8 38036770 PMC12159423

[B175] Poseidon Group (Patient-Oriented Strategies Encompassing IndividualizeD Oocyte Number) Alviggi C. Andersen C. Y. Buehler K. Conforti A. De Placido G. (2016). A new more detailed stratification of low responders to ovarian stimulation: from a poor ovarian response to a low prognosis concept. Fertil. Steril. 105 (6), 1452–1453. 10.1016/j.fertnstert.2016.02.005 26921622

[B176] Prasad S. Tiwari M. Pandey A. N. Shrivastav T. G. Chaube S. K. (2016). Impact of stress on oocyte quality and reproductive outcome. J. Biomed. Sci. 23, 36. 10.1186/s12929-016-0253-4 27026099 PMC4812655

[B177] Ratts V. S. Flaws J. A. Kolp R. Sorenson C. M. Tilly J. L. (1995). Ablation of bcl-2 gene expression decreases the numbers of oocytes and primordial follicles established in the post-natal female mouse gonad. Endocrinol. 136 (8), 3665–3668. 10.1210/endo.136.8.7628407 7628407

[B178] Roberts V. H. J. Pound L. D. Thorn S. R. Gillingham M. B. Thornburg K. L. Friedman J. E. (2014). Beneficial and cautionary outcomes of resveratrol supplementation in pregnant nonhuman primates. FASEB J. 28 (6), 2466–2477. 10.1096/fj.13-245472 24563374 PMC4021444

[B179] Rodrigues P. Limback D. McGinnis L. K. Plancha C. E. Albertini D. F. (2009). Multiple mechanisms of germ cell loss in the perinatal mouse ovary. Reprod. Camb. Engl. 137 (4), 709–720. 10.1530/REP-08-0203 19176312

[B180] Roseira J. Lopes R. Silva M. J. Vieira A. M. Sampaio M. Calinas F. (2022). Gynecological history to diagnosis and pregnancy outcomes in diagnosed Wilson’s disease patients under therapy - a bicentric matched-control cohort study. Rev. Esp. Enferm. Dig. 114 (4), 198–203. 10.17235/reed.2020.7444/2020 33393331

[B181] Sahoo G. Samal D. Khandayataray P. Murthy M. K. (2023). A review on caspases: key regulators of biological activities and apoptosis. Mol. Neurobiol. 60 (10), 5805–5837. 10.1007/s12035-023-03433-5 37349620

[B182] Saman M. Khazaei M. Rashidi Z. (2023). Synergistic effects of capsaicin and quercetin improved induced premature ovarian failure in rat. Cell. J. Yakhteh 25 (7), 496–504. 10.22074/CELLJ.2023.1989732.1234 PMC10404358 37543862

[B183] Sammad A. Ahmed T. Ullah K. Hu L. Luo H. Alphayo Kambey P. (2024). Vitamin C alleviates the negative effects of heat stress on reproductive processes by regulating amino acid metabolism in granulosa cells. Antioxid. Basel Switz. 13 (6), 653. 10.3390/antiox13060653 PMC11201207 38929092

[B184] Seli E. Wang T. Horvath T. L. (2019). Mitochondrial unfolded protein response: a stress response with implications for fertility and reproductive aging. Fertil. Steril. 111 (2), 197–204. 10.1016/j.fertnstert.2018.11.048 30691623

[B185] Shakibaei M. Schulze-Tanzil G. Takada Y. Aggarwal B. B. (2005). Redox regulation of apoptosis by members of the TNF superfamily. Antioxid. Redox Signal 7 (3-4), 482–496. 10.1089/ars.2005.7.482 15706096

[B186] Shalini S. Dorstyn L. Dawar S. Kumar S. (2015). Old, new and emerging functions of caspases. Cell. Death Differ. 22 (4), 526–539. 10.1038/cdd.2014.216 25526085 PMC4356345

[B187] Shang Z. Fan M. Zhang J. Wang Z. Jiang S. Li W. (2023). Red ginseng improves D-galactose-induced premature ovarian failure in mice based on network pharmacology. Int. J. Mol. Sci. 24 (9), 8210. 10.3390/ijms24098210 37175917 PMC10179375

[B188] Shao T. Ke H. Liu R. Xu L. Han S. Zhang X. (2022). Autophagy regulates differentiation of ovarian granulosa cells through degradation of WT1. Autophagy 18 (8), 1864–1878. 10.1080/15548627.2021.2005415 35025698 PMC9450966

[B189] Sharma R. Biedenharn K. R. Fedor J. M. Agarwal A. (2013). Lifestyle factors and reproductive health: taking control of your fertility. Reprod. Biol. Endocrinol. 11, 66. 10.1186/1477-7827-11-66 23870423 PMC3717046

[B190] Shen M. Jiang Y. Guan Z. Cao Y. Li L. Liu H. (2017). Protective mechanism of FSH against oxidative damage in mouse ovarian granulosa cells by repressing autophagy. Autophagy 13 (8), 1364–1385. 10.1080/15548627.2017.1327941 28598230 PMC5584866

[B191] Shen M. Jiang Y. Guan Z. Cao Y. Sun S. C. Liu H. (2016). FSH protects mouse granulosa cells from oxidative damage by repressing mitophagy. Sci. Rep. 6, 38090. 10.1038/srep38090 27901103 PMC5128862

[B192] Sirotkin A. V. Alexa R. Alwasel S. Harrath A. H. (2021). Fennel affects ovarian cell proliferation, apoptosis, and response to ghrelin. Physiol. Res. 70 (2), 237–243. 10.33549/physiolres.934546 33992047 PMC8820581

[B193] Sirotkin A. V. Alexa R. Harrath A. H. (2020). Puncturevine (*Tribulus terrestris* L.) affects the proliferation, apoptosis, and ghrelin response of ovarian cells. Reprod. Biol. 20 (1), 33–36. 10.1016/j.repbio.2019.12.009 31924507

[B194] Sklan E. H. Lowenthal A. Korner M. Ritov Y. Landers D. M. Rankinen T. (2004). Acetylcholinesterase/paraoxonase genotype and expression predict anxiety scores in health, risk factors, exercise training, and genetics study. Proc. Natl. Acad. Sci. U. S. A. 101 (15), 5512–5517. 10.1073/pnas.0307659101 15060281 PMC397414

[B195] Solon M. Ge N. Hambro S. Haller S. Jiang J. Baca M. (2024). ZBP1 and TRIF trigger lethal necroptosis in mice lacking caspase-8 and TNFR1. Cell. Death Differ. 31 (5), 672–682. 10.1038/s41418-024-01286-6 38548850 PMC11093969

[B196] Song X. Zhu S. Chen P. Hou W. Wen Q. Liu J. (2018). AMPK-mediated BECN1 phosphorylation promotes ferroptosis by directly blocking system xc- activity. Curr. Biol. 28 (15), 2388–2399. 10.1016/j.cub.2018.05.094 30057310 PMC6081251

[B197] Song Z. H. Yu H. Y. Wang P. Mao G. K. Liu W. X. Li M. N. (2015). Germ cell-specific Atg7 knockout results in primary ovarian insufficiency in female mice. Cell. Death Dis. 6 (1), e1589. 10.1038/cddis.2014.559 25590799 PMC4669757

[B198] Sonigo C. Beau I. Grynberg M. Binart N. (2019). AMH prevents primordial ovarian follicle loss and fertility alteration in cyclophosphamide-treated mice. FASEB J. 33 (1), 1278–1287. 10.1096/fj.201801089R 30113879

[B199] Stadtmauer L. Vidali A. Lindheim S. R. Sauer M. V. (1998). Follicular fluid insulin-like growth factor-I and insulin-like growth factor-binding protein-1 and -3 vary as a function of ovarian reserve and ovarian stimulation. J. Assist. Reprod. Genet. 15 (10), 587–593. 10.1023/a:1020377209952 9866066 PMC3454853

[B200] Steiner A. Z. Pritchard D. Stanczyk F. Z. Kesner J. S. Meadows J. W. Herring A. H. (2017). Association between biomarkers of ovarian reserve and infertility among older women of reproductive age. JAMA 318 (14), 1367–1376. 10.1001/jama.2017.14588 29049585 PMC5744252

[B201] Stockwell B. R. Friedmann Angeli J. P. Bayir H. Bush A. I. Conrad M. Dixon S. J. (2017). Ferroptosis: a regulated cell death nexus linking metabolism, redox biology, and disease. Cell. 171 (2), 273–285. 10.1016/j.cell.2017.09.021 28985560 PMC5685180

[B202] Suh E. K. Yang A. Kettenbach A. Bamberger C. Michaelis A. H. Zhu Z. (2006). p63 protects the female germ line during meiotic arrest. Nature 444 (7119), 624–628. 10.1038/nature05337 17122775

[B203] Sun D. Wang Y. Sun N. Jiang Z. Wang L. (2023a). LncRNA DANCR counteracts premature ovarian insufficiency by regulating the senescence process of granulosa cells through stabilizing the interaction between p53 and hNRNPC. J. Ovarian Res. 16, 41. 10.1186/s13048-023-01115-3 36805799 PMC9938559

[B204] Sun J. Gan L. Lv S. Wang T. Dai C. Sun J. (2023b). Exposure to Di-(2-Ethylhexyl) phthalate drives ovarian dysfunction by inducing granulosa cell pyroptosis via the SLC39A5/NF-κB/NLRP3 axis. Ecotoxicol. Environ. Saf. 252, 114625. 10.1016/j.ecoenv.2023.114625 36774801

[B205] Sun Y. C. Wang Y. Y. Sun X. F. Cheng S. F. Li L. Zhao Y. (2018). The role of autophagy during murine primordial follicle assembly. Aging 10 (2), 197–211. 10.18632/aging.101376 29410391 PMC5842841

[B206] Sze S. C. W. Zhang L. Zhang S. Lin K. Ng T. B. Ng M. L. (2022). Aberrant transferrin and ferritin upregulation elicits iron accumulation and oxidative inflammaging causing ferroptosis and undermines estradiol biosynthesis in aging rat ovaries by upregulating NF-Κb-Activated inducible nitric oxide synthase: first demonstration of an intricate mechanism. Int. J. Mol. Sci. 23 (20), 12689. 10.3390/ijms232012689 36293552 PMC9604315

[B207] Talebi A. Roodbari N. H. Sameni H. R. Zarbakhsh S. (2020). Impact of coadministration of apigenin and bone marrow stromal cells on damaged ovaries due to chemotherapy in rat: an experimental study. Int. J. Reprod. Biomed. 18 (7), 551–560. 10.18502/ijrm.v13i7.7372 32803119 PMC7385912

[B208] Taneja N. Tjalkens R. Philbert M. A. Rehemtulla A. (2001). Irradiation of mitochondria initiates apoptosis in a cell free system. Oncogene 20 (2), 167–177. 10.1038/sj.onc.1204054 11313941

[B209] Tanner E. A. Blute T. A. Brachmann C. B. McCall K. (2011). Bcl-2 proteins and autophagy regulate mitochondrial dynamics during programmed cell death in the Drosophila ovary. Dev. Camb. Engl. 138 (2), 327–338. 10.1242/dev.057943 PMC3005606 21177345

[B210] Tao F. Zhai Q. Cao Y. Gao H. Cai Y. Jia W. (2024). Inhibition of p38 MAPK/NF-κB p65 signaling pathway activity by rare ginsenosides ameliorates cyclophosphamide-induced premature ovarian failure and KGN cell injury. J. Ethnopharmacol. 326, 117944. 10.1016/j.jep.2024.117944 38382656

[B211] Tartagni M. Cicinelli M. V. Baldini D. Tartagni M. V. Alrasheed H. DeSalvia M. A. (2015). Dehydroepiandrosterone decreases the age-related decline of the *in vitro* fertilization outcome in women younger than 40 years old. Reprod. Biol. Endocrinol. 13, 18. 10.1186/s12958-015-0014-3 25884390 PMC4355976

[B212] Terranova P. F. (1997). Potential roles of tumor necrosis factor-alpha in follicular development, ovulation, and the life span of the corpus luteum. Domest. Anim. Endocrinol. 14 (1), 1–15. 10.1016/s0739-7240(96)00094-x 8985665

[B213] Texada M. J. Malita A. Rewitz K. (2019). Autophagy regulates steroid production by mediating cholesterol trafficking in endocrine cells. Autophagy 15 (8), 1478–1480. 10.1080/15548627.2019.1617608 31084464 PMC6613879

[B214] Tilly J. L. Billig H. Kowalski K. I. Hsueh A. J. (1992). Epidermal growth factor and basic fibroblast growth factor suppress the spontaneous onset of apoptosis in cultured rat ovarian granulosa cells and follicles by a tyrosine kinase-dependent mechanism. Mol. Endocrinol. Balt. Md 6 (11), 1942–1950. 10.1210/mend.6.11.1480180 1480180

[B215] Tilly J. L. Kowalski K. I. Johnson A. L. Hsueh A. J. (1991). Involvement of apoptosis in ovarian follicular atresia and postovulatory regression. Endocrinol. 129 (5), 2799–2801. 10.1210/endo-129-5-2799 1718732

[B216] Toda K. Takeda K. Okada T. Akira S. Saibara T. Kaname T. (2001). Targeted disruption of the aromatase P450 gene (Cyp19) in mice and their ovarian and uterine responses to 17beta-oestradiol. J. Endocrinol. 170 (1), 99–111. 10.1677/joe.0.1700099 11431142

[B217] Tower J. (2015). Programmed cell death in aging. Ageing Res. Rev. 23, 90–100. 10.1016/j.arr.2015.04.002 25862945 PMC4480161

[B218] Tsui K. H. Wang P. H. Lin L. T. Li C. J. (2017). DHEA protects mitochondria against dual modes of apoptosis and necroptosis in human granulosa HO23 cells. Reprod. Camb. Engl. 154 (2), 101–110. 10.1530/REP-17-0016 28624766

[B219] Umehara T. Winstanley Y. E. Andreas E. Morimoto A. Williams E. J. Smith K. M. (2022). Female reproductive life span is extended by targeted removal of fibrotic collagen from the mouse ovary. Sci. Adv. 8 (24), eabn4564. 10.1126/sciadv.abn4564 35714185 PMC9205599

[B220] Valckx S. D. Arias-Alvarez M. De Pauw I. Fievez V. Vlaeminck B. Fransen E. (2014). Fatty acid composition of the follicular fluid of normal weight, overweight and obese women undergoing assisted reproductive treatment: a descriptive cross-sectional study. Reprod. Biol. Endocrinol. 12, 13. 10.1186/1477-7827-12-13 24498875 PMC3916060

[B221] Wang C. Sun H. Davis J. S. Wang X. Huo L. Sun N. (2023b). FHL2 deficiency impairs follicular development and fertility by attenuating EGF/EGFR/YAP signaling in ovarian granulosa cells. Cell. Death Dis. 14 (4), 239. 10.1038/s41419-023-05759-3 37015904 PMC10073124

[B222] Wang C. Yang T. Xiao J. Xu C. Alippe Y. Sun K. (2021). NLRP3 inflammasome activation triggers gasdermin D-independent inflammation. Sci. Immunol. 6 (64), eabj3859. 10.1126/sciimmunol.abj3859 34678046 PMC8780201

[B223] Wang D. Weng Y. Zhang Y. Wang R. Wang T. Zhou J. (2020a). Exposure to hyperandrogen drives ovarian dysfunction and fibrosis by activating the NLRP3 inflammasome in mice. Sci. Total Environ. 745, 141049. 10.1016/j.scitotenv.2020.141049 32758727

[B224] Wang F. Liu Y. Ni F. Jin J. Wu Y. Huang Y. (2022b). BNC1 deficiency-triggered ferroptosis through the NF2-YAP pathway induces primary ovarian insufficiency. Nat. Commun. 13 (1), 5871. 10.1038/s41467-022-33323-8 36198708 PMC9534854

[B225] Wang F. Min J. (2021). DHODH tangoing with GPX4 on the ferroptotic stage. Signal Transduct. Target Ther. 6 (1), 244. 10.1038/s41392-021-00656-7 34145214 PMC8212586

[B226] Wang H. X. Lu X. L. Huang W. J. Zhang J. M. (2019). Pyroptosis is involved in cryopreservation and auto-transplantation of mouse ovarian tissues and pyroptosis inhibition improves ovarian graft function. Res. Vet. Sci. 124, 52–56. 10.1016/j.rvsc.2019.02.004 30849614

[B227] Wang J. J. Ge W. Zhai Q. Y. Liu J. C. Sun X. W. Liu W. X. (2020c). Single-cell transcriptome landscape of ovarian cells during primordial follicle assembly in mice. PLoS Biol. 18 (12), e3001025. 10.1371/journal.pbio.3001025 33351795 PMC7787681

[B228] Wang L. Liu Y. Du T. Yang H. Lei L. Guo M. (2020b). ATF3 promotes erastin-induced ferroptosis by suppressing system Xc. Cell. Death Differ. 27 (2), 662–675. 10.1038/s41418-019-0380-z 31273299 PMC7206049

[B229] Wang S. Li X. Li J. Wang A. Li F. Hu H. (2024). Inhibition of cisplatin-induced Acsl4-mediated ferroptosis alleviated ovarian injury. Chem. Biol. Interact. 387, 110825. 10.1016/j.cbi.2023.110825 38056807

[B230] Wang S. Long H. Hou L. Feng B. Ma Z. Wu Y. (2023d). The mitophagy pathway and its implications in human diseases. Signal Transduct. Target Ther. 8 (1), 304. 10.1038/s41392-023-01503-7 37582956 PMC10427715

[B231] Wang X. Yang J. Li H. Mu H. Zeng L. Cai S. (2023a). miR-484 mediates oxidative stress-induced ovarian dysfunction and promotes granulosa cell apoptosis via SESN2 downregulation. Redox Biol. 62, 102684. 10.1016/j.redox.2023.102684 36963287 PMC10060268

[B232] Wang X. Yuan P. Zeng M. Sun M. Zheng X. (2023c). Allantoin derived from Dioscorea opposita thunb ameliorates cyclophosphamide-induced premature ovarian failure in female rats by attenuating apoptosis, autophagy and pyroptosis. Cureus 15 (12), e50351. 10.7759/cureus.50351 38089953 PMC10713354

[B233] Wang Y. Gao W. Shi X. Ding J. Liu W. He H. (2017). Chemotherapy drugs induce pyroptosis through caspase-3 cleavage of a gasdermin. Nature 547 (7661), 99–103. 10.1038/nature22393 28459430

[B234] Wang Y. Li C. Ali I. Li L. Wang G. (2020d). N-acetylcysteine modulates non-esterified fatty acid-induced pyroptosis and inflammation in granulosa cells. Mol. Immunol. 127, 157–163. 10.1016/j.molimm.2020.09.011 32987256

[B235] Wang Y. Xu Y. Guo W. Fang Y. Hu L. Wang R. (2022a). Ablation of Shank3 alleviates cardiac dysfunction in aging mice by promoting CaMKII activation and Parkin-mediated mitophagy. Redox Biol. 58, 102537. 10.1016/j.redox.2022.102537 36436456 PMC9709154

[B236] Wang Y. F. Sun X. F. Han Z. L. Li L. Ge W. Zhao Y. (2018). Protective effects of melatonin against nicotine-induced disorder of mouse early folliculogenesis. Aging 10 (3), 463–480. 10.18632/aging.101405 29615536 PMC5892698

[B237] Wang Z. Miao G. Xue X. Guo X. Yuan C. Wang Z. (2016). The vici syndrome protein EPG5 is a Rab7 effector that determines the fusion specificity of autophagosomes with late endosomes/lysosomes. Mol. Cell. 63 (5), 781–795. 10.1016/j.molcel.2016.08.021 27588602

[B238] Wang Z. Xiong Y. Peng Y. Zhang X. Li S. Peng Y. (2023e). Natural product evodiamine-inspired medicinal chemistry: anticancer activity, structural optimization and structure-activity relationship. Eur. J. Med. Chem. 247, 115031. 10.1016/j.ejmech.2022.115031 36549115

[B239] Wang Z. Yang A. Bao H. Wang A. Deng X. Xue D. (2022c). Effect of dehydroepiandrosterone administration before *in vitro* fertilization on the live birth rate in poor ovarian responders according to the Bologna criteria: a randomised controlled trial. BJOG Int. J. Obstet. Gynaecol. 129 (7), 1030–1038. 10.1111/1471-0528.17045 34882964

[B240] Wiser A. Gonen O. Ghetler Y. Shavit T. Berkovitz A. Shulman A. (2010). Addition of dehydroepiandrosterone (DHEA) for poor-responder patients before and during IVF treatment improves the pregnancy rate: a randomized prospective study. Hum. Reprod. Oxf. Engl. 25 (10), 2496–2500. 10.1093/humrep/deq220 20729538

[B241] Won J. H. Park S. Hong S. Son S. Yu J. W. (2015). Rotenone-induced impairment of mitochondrial electron transport chain confers a selective priming signal for NLRP3 inflammasome activation. J. Biol. Chem. 290 (45), 27425–27437. 10.1074/jbc.M115.667063 26416893 PMC4646374

[B242] Wu D. Zhao W. Xu C. Zhou X. Leng X. Li Y. (2022). Melatonin suppresses serum starvation-induced autophagy of ovarian granulosa cells in premature ovarian insufficiency. BMC Womens Health 22 (1), 474. 10.1186/s12905-022-02056-7 36434569 PMC9700896

[B243] Wu H. Liu Q. Yang N. Xu S. (2023b). Polystyrene-microplastics and DEHP co-exposure induced DNA damage, cell cycle arrest and necroptosis of ovarian granulosa cells in mice by promoting ROS production. Sci. Total Environ. 871, 161962. 10.1016/j.scitotenv.2023.161962 36775173

[B244] Wu J. Li J. Liu Y. Liao X. Wu D. Chen Y. (2021). Tannic acid repair of zearalenone-induced damage by regulating the death receptor and mitochondrial apoptosis signaling pathway in mice. Environ. Pollut. Barking Essex 1987 287, 117557. 10.1016/j.envpol.2021.117557 34167001

[B245] Wu M. Tang W. Chen Y. Xue L. Dai J. Li Y. (2024). Spatiotemporal transcriptomic changes of human ovarian aging and the regulatory role of FOXP1. Nat. Aging 4 (4), 527–545. 10.1038/s43587-024-00607-1 38594460 PMC11031396

[B246] Wu Y. Zhou S. Zhao A. Mi Y. Zhang C. (2023a). Protective effect of rutin on ferroptosis-induced oxidative stress in aging laying hens through Nrf2/HO-1 signaling. Cell. Biol. Int. 47 (3), 598–611. 10.1002/cbin.11960 36378583

[B247] Wu Y. Y. Liang C. Y. Liu T. T. Liang Y. M. Li S. J. Lu Y. Y. (2018). Protective roles and mechanisms of polysaccharides from Dendrobium officinal on natural aging-induced premature ovarian failure. Biomed. Pharmacother. 101, 953–960. 10.1016/j.biopha.2018.03.030 29635905

[B248] Xi H. Wang Z. Li M. Duan X. Li Y. (2024). Paeoniflorin promotes ovarian development in mice by activating mitophagy and preventing oxidative stress. Int. J. Mol. Sci. 25 (15), 8355. 10.3390/ijms25158355 39125927 PMC11313479

[B249] Xie J. Yang Y. Zhuo A. Gao M. Tang L. Xiao Y. (2024). Exosomes derived from mesenchymal stem cells attenuate NLRP3-related pyroptosis in autoimmune premature ovarian insufficiency via the NF-κB pathway. Reprod. Biomed. Online 48 (6), 103814. 10.1016/j.rbmo.2024.103814 38569224

[B250] Xie Q. E. Wang M. Y. Cao Z. P. Du X. Ji D. M. Liang D. (2021). Melatonin protects against excessive autophagy-induced mitochondrial and ovarian reserve function deficiency though ERK signaling pathway in Chinese hamster ovary (CHO) cells. Mitochondrion 61, 44–53. 10.1016/j.mito.2021.09.009 34571250

[B251] Xie Y. Kang R. Klionsky D. J. Tang D. (2023). GPX4 in cell death, autophagy, and disease. Autophagy 19 (10), 2621–2638. 10.1080/15548627.2023.2218764 37272058 PMC10472888

[B252] Xin L. Li F. Yu H. Xiong Q. Hou Q. Meng Y. (2023). Honokiol alleviates radiation-induced premature ovarian failure via enhancing Nrf2. Am. J. Reprod. Immunol. N. Y. N. 1989 90 (4), e13769. 10.1111/aji.13769 37766410

[B253] Xu G. Dong Y. Wang Z. Ding H. Wang J. Zhao J. (2023a). Melatonin attenuates oxidative stress-induced apoptosis of bovine ovarian granulosa cells by promoting mitophagy via SIRT1/FoxO1 signaling pathway. Int. J. Mol. Sci. 24 (16), 12854. 10.3390/ijms241612854 37629033 PMC10454225

[B254] Xu M. Li F. Xu X. Hu N. Miao J. Zhao Y. (2024). Proteomic analysis reveals that cigarette smoke exposure diminishes ovarian reserve in mice by disrupting the CREB1-mediated ovarian granulosa cell proliferation-apoptosis balance. Ecotoxicol. Environ. Saf. 271, 115989. 10.1016/j.ecoenv.2024.115989 38242047

[B255] Xu R. Zhao H. Qi J. Yao G. He Y. Lu Y. (2023b). Local glucose elevation activates pyroptosis via NLRP3 inflammasome in ovarian granulosa cells of overweight patients. FASEB J. 37 (3), e22807. 10.1096/fj.202201796RR 36826432

[B256] Xu Y. Nisenblat V. Lu C. Li R. Qiao J. Zhen X. (2018). Pretreatment with coenzyme Q10 improves ovarian response and embryo quality in low-prognosis young women with decreased ovarian reserve: a randomized controlled trial. Reprod. Biol. Endocrinol. 16 (1), 29. 10.1186/s12958-018-0343-0 29587861 PMC5870379

[B257] Yan Z. Dai Y. Fu H. Zheng Y. Bao D. Yin Y. (2018). Curcumin exerts a protective effect against premature ovarian failure in mice. J. Mol. Endocrinol. 60 (3), 261–271. 10.1530/JME-17-0214 29437881 PMC5863768

[B258] Yang X. Wu L. L. Chura L. R. Liang X. Lane M. Norman R. J. (2012). Exposure to lipid-rich follicular fluid is associated with endoplasmic reticulum stress and impaired oocyte maturation in cumulus-oocyte complexes. Fertil. Steril. 97 (6), 1438–1443. 10.1016/j.fertnstert.2012.02.034 22440252

[B259] Yang Y. Klionsky D. J. (2020). Autophagy and disease: unanswered questions. Cell. Death Differ. 27 (3), 858–871. 10.1038/s41418-019-0480-9 31900427 PMC7206137

[B260] Yang Z. Klionsky D. J. (2010). Mammalian autophagy: core molecular machinery and signaling regulation. Curr. Opin. Cell. Biol. 22 (2), 124–131. 10.1016/j.ceb.2009.11.014 20034776 PMC2854249

[B261] Yao H. Liu J. Xu S. Zhu Z. Xu J. (2017). The structural modification of natural products for novel drug discovery. Expert Opin. Drug Discov. 12 (2), 121–140. 10.1080/17460441.2016.1272757 28006993

[B262] Yao X. Wang C. Yu W. Sun L. Lv Z. Xie X. (2023). SRSF1 is essential for primary follicle development by regulating granulosa cell survival via mRNA alternative splicing. Cell. Mol. Life Sci. 80 (11), 343. 10.1007/s00018-023-04979-2 37907803 PMC11072053

[B263] Yeung T. W. Y. Chai J. Li R. H. W. Lee V. C. Y. Ho P. C. Ng E. H. Y. (2014). A randomized, controlled, pilot trial on the effect of dehydroepiandrosterone on ovarian response markers, ovarian response, and *in vitro* fertilization outcomes in poor responders. Fertil. Steril. 102 (1), 108–115. 10.1016/j.fertnstert.2014.03.044 24796766

[B264] Yi Y. Hao Z. Sun P. Fan K. Yin W. Guo J. (2022). Study on the mechanism of scutellarin’s protective effect against ZEA-induced mouse ovarian granulosa cells injury. Food Chem. Toxicol. 170, 113481. 10.1016/j.fct.2022.113481 36252740

[B265] Yi Y. Wan S. Wang S. Khan A. Guo J. Zheng X. (2021). Scutellarin protects mouse ovarian granulosa cells from injury induced by the toxin zearalenone. Food Funct. 12 (3), 1252–1261. 10.1039/D0FO02711A 33433546

[B266] Yoshino J. Baur J. A. Imai S. I. (2018). NAD+ intermediates: the biology and therapeutic potential of NMN and NR. Cell. Metab. 27 (3), 513–528. 10.1016/j.cmet.2017.11.002 29249689 PMC5842119

[B267] Yuan X. Hu T. Zhao H. Huang Y. Ye R. Lin J. (2016). Brown adipose tissue transplantation ameliorates polycystic ovary syndrome. Proc. Natl. Acad. Sci. U. S. A. 113 (10), 2708–2713. 10.1073/pnas.1523236113 26903641 PMC4790997

[B268] Yuan X. Ma W. Chen S. Wang H. Zhong C. Gao L. (2023). CLPP inhibition triggers apoptosis in human ovarian granulosa cells via COX5A abnormality-Mediated mitochondrial dysfunction. Front. Genet. 14, 1141167. 10.3389/fgene.2023.1141167 37007963 PMC10065195

[B269] Zhang D. Liu Y. Zhang Z. Lv P. Li J. Wu Y. (2018). Basonuclin 1 deficiency is a cause of primary ovarian insufficiency. Hum. Mol. Genet. 27 (21), 3787–3800. 10.1093/hmg/ddy261 30010909

[B270] Zhang H. H. Xu P. Y. Wu J. Zou W. W. Xu X. M. Cao X. Y. (2014). Dehydroepiandrosterone improves follicular fluid bone morphogenetic protein-15 and accumulated embryo score of infertility patients with diminished ovarian reserve undergoing *in vitro* fertilization: a randomized controlled trial. J. Ovarian Res. 7, 93. 10.1186/s13048-014-0093-3 25330837 PMC4210503

[B271] Zhang J. Sheng S. Wang W. Dai J. Zhong Y. Ren J. (2022). Molecular mechanisms of iron mediated programmed cell death and its roles in eye diseases. Front. Nutr. 9, 844757. 10.3389/fnut.2022.844757 35495915 PMC9038536

[B272] Zhang J. Q. Gao B. W. Wang J. Ren Q. L. Chen J. F. Ma Q. (2016). Critical role of FoxO1 in granulosa cell apoptosis caused by oxidative stress and protective effects of grape seed procyanidin B2. Oxid. Med. Cell. Longev. 2016, 6147345. 10.1155/2016/6147345 27057282 PMC4745910

[B273] Zhang J. Y. Zhou B. Sun R. Y. Ai Y. L. Cheng K. Li F. N. (2021b). The metabolite α-KG induces GSDMC-dependent pyroptosis through death receptor 6-activated caspase-8. Cell. Res. 31 (9), 980–997. 10.1038/s41422-021-00506-9 34012073 PMC8410789

[B274] Zhang S. Liu Q. Chang M. Pan Y. Yahaya B. H. Liu Y. (2023a). Chemotherapy impairs ovarian function through excessive ROS-induced ferroptosis. Cell. Death Dis. 14 (5), 340. 10.1038/s41419-023-05859-0 37225709 PMC10209065

[B275] Zhang T. Bai S. Ding X. Zeng Q. Xuan Y. Xu S. (2024). Pu-erh tea theabrownin improves the ovarian function and gut microbiota in laying hens. Poult. Sci. 103 (7), 103795. 10.1016/j.psj.2024.103795 38723460 PMC11101868

[B276] Zhang T. He M. Zhao L. Qin S. Zhu Z. Du X. (2021a). HDAC6 regulates primordial follicle activation through mTOR signaling pathway. Cell. Death Dis. 12 (6), 559. 10.1038/s41419-021-03842-1 34052832 PMC8164630

[B277] Zhang Y. Bai J. Cui Z. Li Y. Gao Q. Miao Y. (2023b). Polyamine metabolite spermidine rejuvenates oocyte quality by enhancing mitophagy during female reproductive aging. Nat. Aging. 3 (11), 1372–1386. 10.1038/s43587-023-00498-8 37845508

[B278] Zhao F. Yan L. Zhao X. Wu J. Fang Y. Xin Z. (2024). Aberrantly high FBXO31 impairs oocyte quality in premature ovarian insufficiency. Aging Dis. 15 (2), 804–823. 10.14336/AD.2023.0809 37611899 PMC10917549

[B279] Zhao J. Tang M. Tang H. Wang M. Guan H. Tang L. (2023). Sphingosine 1-phosphate alleviates radiation-induced ferroptosis in ovarian granulosa cells by upregulating glutathione peroxidase 4. Reprod. Toxicol. Elmsford N. 115, 49–55. 10.1016/j.reprotox.2022.12.002 36503164

[B280] Zhao Y. T. Yin H. Hu C. Zeng J. Shi X. Chen S. (2022). Tilapia skin peptides restore cyclophosphamide-induced premature ovarian failure via inhibiting oxidative stress and apoptosis in mice. Food Funct. 13 (3), 1668–1679. 10.1039/d1fo04239d 35083997

[B281] Zheng H. Jiang L. Tsuduki T. Conrad M. Toyokuni S. (2021). Embryonal erythropoiesis and aging exploit ferroptosis. Redox Biol. 48, 102175. 10.1016/j.redox.2021.102175 34736120 PMC8577445

[B282] Zheng Y. Ma L. Liu N. Tang X. Guo S. Zhang B. (2019). Autophagy and apoptosis of porcine ovarian granulosa cells during follicular development. Anim. Open Access J. MDPI 9 (12), 1111. 10.3390/ani9121111 PMC6940823 31835576

[B283] Zheng Z. Zuo W. Ye R. Grunberger J. W. Khurana N. Xu X. (2023). Silica nanoparticles promote apoptosis in ovarian granulosa cells via autophagy dysfunction. Int. J. Mol. Sci. 24 (6), 5189. 10.3390/ijms24065189 36982262 PMC10049489

[B284] Zhou C. Guo Q. Lin J. Wang M. Zeng Z. Li Y. (2024b). Single-cell atlas of human ovaries reveals the role of the pyroptotic macrophage in ovarian aging. Adv. Sci. Weinh Baden-Wurtt Ger. 11 (4), e2305175. 10.1002/advs.202305175 PMC10811476 38036420

[B285] Zhou J. Li X. Y. Liu Y. J. Feng J. Wu Y. Shen H. M. (2022a). Full-coverage regulations of autophagy by ROS: from induction to maturation. Autophagy 18 (6), 1240–1255. 10.1080/15548627.2021.1984656 34662529 PMC9225210

[B286] Zhou J. Peng X. Mei S. (2019). Autophagy in ovarian follicular development and atresia. Int. J. Biol. Sci. 15 (4), 726–737. 10.7150/ijbs.30369 30906205 PMC6429023

[B287] Zhou S. Zhao A. Wu Y. Mi Y. Zhang C. (2022b). Protective effect of grape seed proanthocyanidins on oxidative damage of chicken follicular granulosa cells by inhibiting FoxO1-mediated autophagy. Front. Cell. Dev. Biol. 10, 762228. 10.3389/fcell.2022.762228 35242756 PMC8886245

[B288] Zhou W. Chen A. Ye Y. Ren Y. Lu J. Xuan F. (2023). LIPUS combined with TFSC alleviates premature ovarian failure by promoting autophagy and inhibiting apoptosis. Gynecol. Endocrinol. 39 (1), 2258422. 10.1080/09513590.2023.2258422 37855244

[B289] Zhou X. Y. Lai Y. H. Zhang J. Li Y. Wu X. M. Yang Y. Z. (2024a). Advanced oxidation protein products attenuate the autophagy-lysosome pathway in ovarian granulosa cells by modulating the ROS-dependent mTOR-TFEB pathway. Cell. Death Dis. 15 (2), 161. 10.1038/s41419-024-06540-w 38383507 PMC10881514

[B290] Zhou Z. He H. Wang K. Shi X. Wang Y. Su Y. (2020). Granzyme A from cytotoxic lymphocytes cleaves GSDMB to trigger pyroptosis in target cells. Science 368 (6494), eaaz7548. 10.1126/science.aaz7548 32299851

[B291] Zhu J. Lin F. H. Zhang J. Lin J. Li H. Li Y. W. (2016). The signaling pathways by which the Fas/FasL system accelerates oocyte aging. Aging. 8 (2), 291–303. 10.18632/aging.100893 26869336 PMC4789583

[B292] Zhu J. Zhang J. Li H. Wang T. Y. Zhang C. X. Luo M. J. (2015). Cumulus cells accelerate oocyte aging by releasing soluble Fas Ligand in mice. Sci. Rep. 5, 8683. 10.1038/srep08683 25731893 PMC4346792

