# Supplementary material for: Targeting programmed cell death with natural products: a potential therapeutic strategy for diminished ovarian reserve and fertility preservation
Source: Front Pharmacol. 2025 May 29;16:1546041. doi: 10.3389/fphar.2025.1546041 (PMC12158948; doi:10.3389/fphar.2025.1546041)
Supplement: Supplementary file 1 [file Table1.docx]

Appendix 1 Therapeutic potential of natural products in the treatment of DOR: targeting apoptosis

| No. | Natural products | Source | Structure | Optimal dose | Control | Apoptosis-related targets | Potential effect | Adverse effects | References |
| --- | --- | --- | --- | --- | --- | --- | --- | --- | --- |
| 1 | Allantoin isolated from *Dioscorea oppositifolia* L. | *Dioscorea oppositifolia* L. (*Dioscoreaceae*) | Shown in Appendix 6-1 | Vivo: Sprague-Dawley rats,140 mg/kg/d for 3 weeks | Positivel:estradiol valerate; Negative:distilled water | BAX, BCL-2 | Associated with changes in ovarian reserve markers and granulosa cell apoptosis in cyclophosphamide-treated rats, based on preclinical studies | Unreported | ^85^ |
| 2 | α-ketoglutarate | a naturally occurring compound found in the Krebs cycle | Shown in Appendix 6-2 | Vivo: Sprague Dawley rats aged 10 weeks, 250 mg/kg/d for 21 days via gavage | Positivel:-; Negative:normal saline | Bax, Bcl-2, Caspase 3 | Demonstrated potential to improve ovarian reserve, reduce granulosa cell apoptosis, and enhance pregnancy outcomes in a cyclophosphamide-induced POI rat model. | Unreported | ^86^ |
| 3 | Apigenin | *Camellia sinensis* L. Kuntze (*Theaceae*), *Apium* L. (*Apiaceae*)  , and other organisms | Shown in Appendix 6-3 | Vitro: Ovarian granulosa cells of 6-8 months old landrace prepubertal gilts, 100ug/ml, duration of action unknown | Positivel:-; Negative: DMSO | Bax, Caspase3 | Potential modulation of granulosa cell function, including decreased apoptosis and altered hormone secretion; confirmed *in vitro* only. | Unreported | ^87^ |
| 3 | Apigenin | *Camellia sinensis* (L.) Kuntze (*Theaceae*), *Apium* L (*Apiaceae*).  , and other organisms | Shown in Appendix 6-3 | Vivo: Wistar rats age 10 weeks, 10 mg/kg/d for 14 days via i.p | Positivel:-; Negative: DMSO | Bcl-2, Bax | Demonstrated potential to improve ovarian reserve markers in cyclophosphamide-injured rat models. | Unreported | ^88^ |
| 4 | Capsaicin | *Capsicum pubescens* Ruiz & Pav.(*Solanaceae*)  , *Capsicum annuum* L.(*Solanaceae*) | Shown in Appendix 6-4 | Vivo: Wistar rats age 12-16 weeks, 0.5 mg/kg/d for 2 weeks via i.p | Positivel:-; Negative:DMSO | Bcl-2, BAX, p53 | Demonstrated potential to improve ovarian reserve markers in cyclophosphamide-injured rat models. | Unreported | ^89^ |
| 5 | Chrysin | *Apium* L. (*Apiaceae*), *Populus yunnanensis* Dode (Salicaceae), and other organisms | Shown in Appendix 6-5 | Vivo: C57BL/6 mice aged 7-8 weeks, 144.4 mg/kg/d, form of administration unknown, duration of action unknown | Positivel:-; Negative: DMSO and corn oil | Bax, Bcl-2, Caspase-3 | Potential alleviation of inflammation and oxidative stress, potentially improving ovarian function in D-galactose-induced POF mice. | Unreported | ^90^ |
| 5 | Chrysin | *Apium* L. (*Apiaceae*), *Populus yunnanensis* Dode (Salicaceae), and other organisms | Shown in Appendix 6-5 | Vivo: Sprague-Dawley rats aged 3 weeks, 50 mg/kg/d for 17 days via p.o | Positivel:-; Negative:DMSO and corn oil | cytochrome c, caspase-3 | Potential alleviation of inflammation and apoptosis in γ-radiation-induced POF models, possibly via TGF-β/MAPK signaling modulation. | Unreported | ^91^ |
| 6 | Combined *Citrus × limon* (L.) Osbeck peel extract and resveratrol | *Citrus × limon* (L.) Osbeck (*Rutaceae*) ；Various plants, including *Vitaceae* Juss. (*Vitaceae*), *Reynoutria japonica* Houtt.(*Polygonaceae*) | Complex extract, structure not defined; Resveratrol: Shown in Appendix 6-6 | Vivo: Wistar rats aged 12-16 weeks, 10 mg/kg/d for 3 weeks via p.o | Positivel:-; Negative: salt-water | Caspase-3 | Potential to improve ovarian reserve and modulate iNOS/Caspase-3 pathway activity in cyclophosphamide-induced POF rats. | Unreported | ^92^ |
| 7 | *Cuscuta chinensis* Lam | *Cuscuta chinensis* Lam.(*Convolvulaceae*) | Unknown structure | Vitro: human ovarian granulosa cells tumor cells (KGN), 0.3 mg/mL | Positivel:N-acetylcysteine; Negative: no treatment. | Bcl-2, Bax, Cleaved -caspase-9, Cleaved -caspase-3 | Shown to protect KGN cells from H_2_O_2_-induced oxidative stress and apoptosis, possibly via Keap1/Nrf2/HO-1 and PI3K/Akt pathways. | Unreported | ^93^ |
| 8 | Curcumin | *Curcuma longa* L.(*Zingiberaceae*), *Zingiberaceae* Martinov (*Zingiberaceae*) | 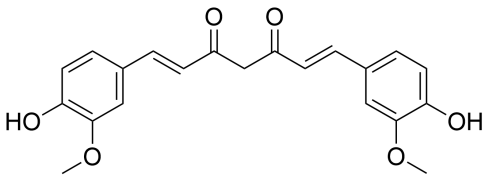 | Vivo: C57BL/6 mice aged 7-8 weeks, 100 mg/kg/d for 6 weeks via i.p | Positivel:-; Negative: salt-water | caspase-3, caspase-9 | Observed reduction in oxidative stress, apoptosis, and ovarian damage in D-galactose-induced models. | Unreported | ^94^ |
| 8 | Curcumin | *Curcuma longa* L.(*Zingiberaceae*), *Zingiberaceae* Martinov (*Zingiberaceae*) | See above | Vivo: ICR mice aged 6-8 weeks,200 mg/kg/d for 14 days via intraperitoneal injection；Vitro:KGN cells,20 μM for 24 hours | Vivo: Positivel:-; Negative: normal saline.  Vitro:  Positivel:N-acetylcysteine; Negative: no treatment. | Bcl-2, Bax | Potential protection of ovarian granulosa cells from H_2_O_2_-induced oxidative stress damage | Unreported | ^95^ |
| 9 | Dehydroepiandrosterone | Synthesized in the adrenal glands in *Homo sapiens;* plant sources, such as *Dioscorea villosa* L. (*Dioscoreaceae*) | Shown in Appendix 6-8 | Vitro: human granulosa cells (HO-23),1 nM for 20 h | Positivel: 5%FBS; Negative: staurosporine | Bax, Bcl-2, Caspase 3, Caspase 9, Cytochrome c | Potential alleviation of serum starvation-induced apoptosis in human granulosa cells *in vitro*. | Unreported | ^96^ |
| 10 | Epigallocatechin-3-gallate | *Camellia sinensis* (L.) Kuntze, *Eschweilera coriacea* (DC.) S.A.Mori, and other organisms | Shown in Appendix 6-9 | Vivo: Swiss mice aged 8 weeks, 50 mg/kg/d for 3 days via i.p | Positivel: N-acetylcysteine; Negative: NaCl solution | Cleaved -caspase-3 | Potential to inhibit oxidative damage and apoptosis in cyclophosphamide-injured ovarian tissue, possibly by regulating p-Akt, p-FOXO3a, and p-rpS6. | Unreported | ^97^ |
| 11 | *Foeniculum vulgare* Mill. extract | *Foeniculum vulgare* Mill. (*Apiaceae*) | Unknown structure | Vitro: porcine ovarian granulosa cells , 10 μg/ml | Positivel: biological-grade human ghrelin; Negative: cell-free medium | PCNA, BAX | Potential alleviation of apoptosis in porcine ovarian granulosa cells *in vitro*. | Unreported | ^98^ |
| 12 | Querceti-Eugenol-Diosgenin mixture (15:6:10) | *Syzygium aromaticum* (L.) Merr. & L.M.Perry (*Myrtaceae*), *Styphnolobium* Schott (*Fabaceae*) and *Dioscorea polystachya* Turcz. (*Dioscoreaceae*) | Mixture of compounds, structures vary; Shown in Appendix 6-10-12 | Vivo: mice at 40 weeks of age, 310 mg/kg for a month via gavage | Positivel:-; Negative: normal saline | Caspase-3, Cleaved -Caspase 3 | Possible reduction of apoptosis, inflammation, and oxidative stress, with fertility improvement in aged mouse ovaries. | Unreported | ^100^ |
| 13 | Procyanidins | *Lathyrus laxiflorus* (Desf.) Kuntze (*Fabaceae*), *Vitis amurensis* Rupr. (*Vitaceae*), and other organisms | Mixture of compounds, structures vary | Vitro: ovaries of D280 (young) and D580 (old) hens, 5μg/mL | Positivel:-; Negative: no treatment | Bax, Bcl-2 | Potential reduction of d-galactose-induced apoptosis and follicular atresia in granulosa cells. | Unreported | ^101^ |
| 13 | Procyanidin B2 | *Lathyrus laxiflorus* (Desf.) Kuntze (*Fabaceae*), *Vitis amurensis* Rupr. (*Vitaceae*), and other organisms | 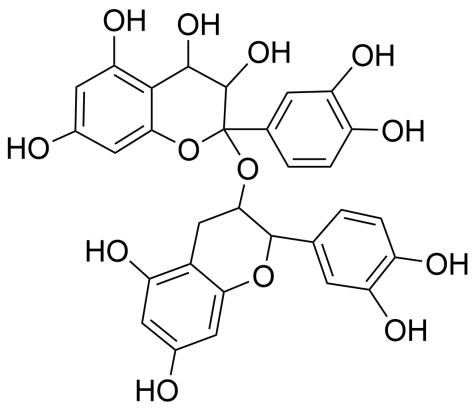 | Vivo: ICR mice, 30 mg/kg for 3 weeks via p.o；Vitro: ovarian granulosa cells of ICR mice, 10μmol/L | Positivel:-; Negative: no treatment | caspase-3, Bcl2/Bax | Potential to reduce diquat-induced ovarian damage and granulosa cell apoptosis in preclinical models. | Unreported | ^102^ |
| 14 | *Panax ginseng* C.A.Mey.  extract | *Panax ginseng* C.A.Mey. (*Araliaceae*) | Unknown structure | Vivo: NMRI mice aged 8-10 weeks, 1g/kg/d for 30 days via gavage | Positivel:-; Negative:1% CMC-saline | Bax, Bcl-2, cytochrome c | Potential to reverse nicotine-induced ovarian apoptosis and influence follicular development and oxidative stress. | Unreported | ^103^ |
| 15 | *Panax ginseng* C.A.Mey. extract | *Panax ginseng* C.A.Mey. (*Araliaceae*) | Unknown structure | Vivo: ICR mice aged 2 weeks,400mg/kg/d for 4 weeks via i.p | Positivel:-; Negative: no treatment | Bax, Bcl-2, caspase-3, cleaved caspase-3, caspase-9, cleaved caspase-9 | Potential improvement of ovarian reserve and estrogen levels in D-galactose-induced POF rats via Nrf2 and PI3K/Akt signaling. | Unreported | ^104^ |
| 16 | Honokiol | *Magnolia officinalis* Rehder & E.H.Wilson (*Magnoliaceae*), *Illicium simonsii* Maxim. (*Schisandraceae*), and other organisms | 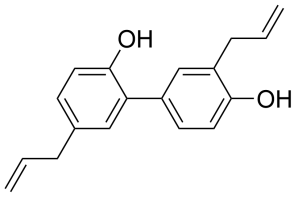 | Vivo: C57BL/6 mice aged 7 weeks,10 mg/kg/d for 1 week via i.p | Positivel:-; Negative:no treatment | P53, Bax, Cyto C, Cleaved -caspase-3 | Potential to improve ovarian reserve in radiation-induced POF models by suppressing inflammation and activating Nrf2/HO-1 pathways. | Unreported | ^105^ |
| 17 | Icariin | *Epimedium brevicornu* Maxim. (*Berberidaceae*), *Epimedium truncatum* H.R.Liang (*Berberidaceae*), and other organisms | 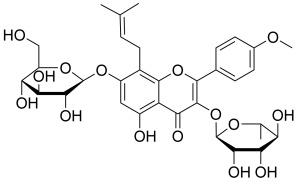 | Vivo: ICR mice aged 6-8 weeks, 30 mg/kg/d for 21d via i.p; Vitro:human ovarian granulosa cells tumor cells (KGN), 5μg/ml for 6h | Positivel:-; Negative: DMSO | Bax | Potential to reduce cisplatin-induced oxidative damage and apoptosis in ovarian tissue. | Unreported | ^106^ |
| 18 | Melatonin | synthesized in the pineal gland in *Homo sapiens;* plant sources, such as *Prunus cerasus* L. (*Rosaceae*) | Shown in Appendix 6-15 | Vivo: C57 BL/6J mice aged 6-8 weeks, Dose unknown, form of administration unknown, duration of action unknown | Positivel:-; Negative:no treatment | Bcl-2 | Potential inhibition of apoptosis in cyclophosphamide-injured ovarian cells via the eIF2α/ATF4 pathway. | Unreported | ^107^ |
| 18 | Melatonin | synthesized in the pineal gland in *Homo sapiens;* plant sources, such as *Prunus cerasus* L. (*Rosaceae*) | Shown in Appendix 6-15 | Vitro: bovine ovarian granulosa cells, 10 μM for 24h | Positivel:-; Negative:no treatment | Bax, Bcl-2, Caspase 3 | Potential inhibition of H_2_O_2_-induced apoptosis. | Unreported | ^108^ |
| 19 | Nobiletin | *Citrus reticulata* Blanco (*Rutaceae*), *Citrus × aurantium f. deliciosa* (Ten.) M.Hiroe (*Rutaceae*), and other organisms | 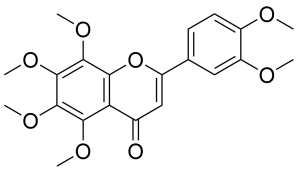 | Vitro: ovarian granulosa cells of hyline white chickens,10 μg/mL for 72h | Positivel:-; Negative: Dorsomorphin | Bax, Bcl-2, caspase-3 | Potential to delay aging-related apoptosis through inhibition of d-galactose-induced oxidative stress in granulosa cells. | Unreported | ^109^ |
| 20 | Polypeptides isolated from *Magallana gigas,Crassostrea virginica,Crassostrea rivularis* | *Magallana gigas (Ostreidae),Crassostrea virginica (Ostreidae),Crassostrea rivularis (Ostreidae), et al* | Unknown structure | Vivo: C57BL/6 mice aged 7-8 weeks,1000 mg/kg/d for 6 weeks via gavage | Positivel:-; Negative: normal saline | Bax, p53 | Potential rescue of D-galactose-induced oxidative damage in ovaries and reduction of apoptosis in ovarian granulosa cells. | Unreported | ^110^ |
| 21 | Paeoniflorin | *Paeonia emodi* Royle (*Paeoniaceae*), *Paeonia obovata* Maxim. (*Paeoniaceae*), and other organisms | Shown in Appendix 6-18 | Vivo: ICR mice aged 3 weeks, 10 mg/kg/d for 7 weeks via intraperitoneal injection | Positivel:-; Negative: normal saline | Bax, Bcl-2 | Potential inhibition of H_2_O_2_-induced oxidative stress and apoptosis. | Unreported | ^111^ |
| 22 | Polysaccharides isolated from *Dendrobium officinale* Kimura & Migo | *Dendrobium officinale* Kimura & Migo (*Orchidaceae*) | Unknown structure | Vivo: Kunming mice aged 15 months, 70 mg/kg/d for 10 weeks via p.o | Positivel:-; Negative: normal saline | Bcl-2, p53 | Potential to reduce apoptosis and oxidative stress in aged ovarian tissues, with improved mitochondrial function. | Unreported | ^112^ |
| 23 | Puerarin | *Bupleurum chinense* DC. (*Apiaceae*), *Pueraria calycina* Franch. (*Fabaceae*), and other organisms | Shown in Appendix 6-19 | Vivo: Kunming mice aged 8 weeks,200 mg/kg/d for 4 weeks via gavage | Positivel:-; Negative: normal saline | Bax, Bcl-2 | Potential to activate the Wnt/β-catenin pathway and reduce oxidative stress from cyclophosphamide and leucovorin in ovarian tissues. | Unreported | ^113^ |
| 24 | Theabrownin | *Camellia sinensis* (L.) Kuntze (*Theaceae*) | Unknown structure | Vivo: Lohman laying hens aged 25 weeks, 100 mg/kg for 12 weeks via p.o. | Positivel:-; Negative: same amount of complete feeding mixture | Bcl-2 | Potential reduction of ovarian cell apoptosis and improvement in estrogen levels in aging models. | Unreported | ^114^ |
| 25 | Quercetin | *Allium cepa* L. (*Amaryllidaceae*), *Malus domestica* (Suckow) Borkh. (*Rosaceae*), *Vitis vinifera* L. (*Vitaceae*), and other organisms | Shown in Appendix 6-20 | Vivo: Wistar rats age 12-16 weeks, 100 mg/kg/d for 2 weeks via i.p | Positivel:-; Negative:DMSO | Bcl-2, Bax, P53 | Demonstrated improvement in ovarian reserve markers in cyclophosphamide-injured rats. | Unreported | ^89^ |
| 26 | Resveratrol | Various plants, including *Vitis vinifera* L. (*Vitaceae*), Reynoutria japonica Houtt. (*Polygonaceae*) | Shown in Appendix 6-6 | Vitro: human primary follicular fluid granulosa cells, 10 μM for 4 hours | Positivel:-; Negative:no treatment | Bax, Bcl-2,Caspase 9 | Observed inhibition of H_2_O_2_-induced apoptosis in human granulosa cells. | Unreported | ^120^ |
| 26 | Resveratrol | Various plants, including *Vitis vinifera* L. (*Vitaceae*), Reynoutria japonica Houtt. (*Polygonaceae*) | Shown in Appendix 6-6 | Vivo:Wistar rats aged 12-14 weeks,10 mg/kg/d for 4 weeks via gavage | Positivel:-; Negative: normal saline | Bax, Bcl-2, Cleaved -Caspase 3 | Potential to reduce cisplatin-induced oxidative damage and apoptosis in ovarian tissue. | Unreported | ^99^ |
| 27 | Scutellarin | *Perilla frutescens* (L.) Britton (*[Lamiaceae](https://powo.science.kew.org/taxon/urn:lsid:ipni.org:names:30000097-2)*  ), *Scutellaria indica* L. (*Lamiaceae*), and other organisms | 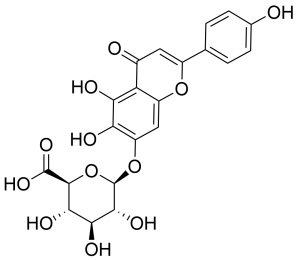 | Vivo:Kunming mice aged 5 weeks,100 mg/kg/d for 3 days via gavage；Vitro: primary ovarian granulosa cells of mouse, 2000 μg/mL for 24 hours | Vivo:Positivel:-; Negative: corn oil；  Vitro: Positivel:-; Negative: DMSO | Caspase-3,Bax,Bcl-2 | Potential to protect granulosa cells from zearalenone-induced apoptosis in mouse models. | Unreported | ^115^ |
| 28 | Tannic acid | *Quercus* L. (*Fagaceae*) | 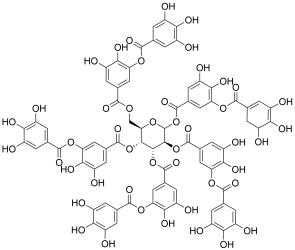 | Vivo: Kunming mice aged 7 weeks, 200 mg/kg/d for 7 days via p.o | Positivel: vitamin E; Negative: 10% ethanol solution | Fas, FasL, Bax, Bcl-2, caspase-3, caspase-8, caspase-9 | Potential reduction of ovarian apoptosis caused by zearalenone-induced oxidative stress. | Unreported | ^116^ |
| 29 | Peptides isolated from *Oreochromis niloticus,Mozambique Tilapia, Blue Tilapia* | *Oreochromis niloticus (Cichlidae),Mozambique Tilapia (Cichlidae), Blue Tilapia (Cichlidae),et al* | Bioactive peptide mixture, no single structure | Vivo: C57BL/6 mice aged 7-8 weeks, 1000 mg/kg/d for 4 weeks via gavage | Positivel:-; Negative: normal saline | Bax, Bcl-2, caspase 3 | Potential protection against cyclophosphamide-induced oxidative stress and granulosa cell apoptosis, with restoration of ovarian reserve in mice. | Unreported | ^117^ |
| 30 | *Tribulus terrestris* L. | *Tribulus terrestris* L. (*Zygophyllaceae*) | Unknown structure | Vitro: Porcine ovarian granulosa cells , 10 μg/ml for 2 days | Positivel: ghrelin; Negative: no treatment | Bax | Potential to reverse apoptosis stimulated by growth hormone-releasing peptide *in vitro*. | Simultaneously promotion of cell proliferation and apoptosis marker expression | ^118^ |
| 31 | The total flavonoids from *Cuscuta chinensis* Lam. | *Cuscuta chinensis* Lam.(*Convolvulaceae*) | Unknown structure | Vivo: Sprague Dawley rats, unknown age, 140 mg/kg/d for 15 days via gavage | Positivel:-; Negative: unknown | Bcl-2, p62, Bax | Potential to improve ovarian reserve in tretinoin and cyclophosphamide-induced POF rats with ultrasound-assisted therapy. | Unreported | ^119^ |

Note: Only representative structures with pharmacological significance or structural complexity are shown; a full list is provided in Supplementary Appendix 6. Potential effects listed are based on experimental models. In vitro-only data do not indicate clinical efficacy. In vivo findings are preliminary and require further validation.
