# Supplementary material for: Targeting programmed cell death with natural products: a potential therapeutic strategy for diminished ovarian reserve and fertility preservation
Source: Front Pharmacol. 2025 May 29;16:1546041. doi: 10.3389/fphar.2025.1546041 (PMC12158948; doi:10.3389/fphar.2025.1546041)
Supplement: Supplementary file 2 [file Table2.docx]

Appendix 2 Therapeutic potential of natural products in the treatment of DOR: targeting autophagy

| No. | Natural products | Activation or inhibition of autophagy | Source | Structure | Optimal dose | Control | Autophagy-related targets | Potential effect | Adverse effects | References |
| --- | --- | --- | --- | --- | --- | --- | --- | --- | --- | --- |
| 1 | Curcumin | Activation | *Curcuma longa* L.(*Zingiberaceae*), *Zingiberaceae* Martinov (*Zingiberaceae*) | 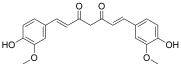 | Vivo: ICR mice aged 6-8 weeks, 200 mg/kg/d for 14 days via intraperitoneal injection; Vitro: KGN cells, 20 μM for 24 hours | Vivo: Positivel:-; Negative: normal saline.  Vitro:  Positivel:N-acetylcysteine; Negative: no treatment. | Beclin-1, LC-3 II/I，AMPK, mTOR | Potential inhibition of AMPK/mTOR pathway activation and activation of autophagy to protect cells from H_2_O_2_-induced oxidative stress damage | Unreported | ^95^ |
| 2 | Procyanidin B2 | Activation | *Lathyrus laxiflorus* (Desf.) Kuntze (*Fabaceae*), *Vitis amurensis* Rupr. (*Vitaceae*), and other organisms | Shown in Appendix 6-13 | Vitro: ovarian granulosa cells of ICR mice, 10μ mol/L, duration of action unknown | Positivel:- ; Negative:no treatment | Atg12, LC3-Ⅱ, Beclin-1 | Potential promotion of autophagy and reversal of H2O2-induced autophagy limitation | Unreported | ^102^ |
| 3 | Melatonin | Activation | synthesized in the pineal gland in *Homo sapiens;* plant sources, such as *Prunus cerasus* L. (*Rosaceae*) | Shown in Appendix 6-15 | Vitro: bovine ovarian granulosa cells, 10 μM for 24h | Positivel:-; Negative:no treatment | PINK1, Parkin, Beclin-1, LC3-Ⅱ/LC3-Ⅰ, SQSMT1 | Potential promotion of mitochondrial and lysosomal fusion and inhibition of H2O2-induced cellular damage | Unreported | ^108^ |
| 3 | Melatonin | Activation | synthesized in the pineal gland in *Homo sapiens;* plant sources, such as *Prunus cerasus* L. (*Rosaceae*) | Shown in Appendix 6-15 | Vitro: follicular fluid granulosa cells from POI patient, 10^-3^M, duration of action unknown | Positivel:-; Negative:no treatment | LC3-Ⅱ, Atg7, Beclin-1, p62 | Potential increase in autophagy-related protein levels in a concentration-dependent manner to promote granulocyte proliferation | Unreported | ^153^ |
| 4 | Nicotinamide mononucleotide | Activation | *Brassica oleracea* L. (*Brassicaceae*), *Brassica oleracea* L. (Brassicaceae), *Cucumis sativus* L. (*Cucurbitaceae*) and other organisms | Shown in Appendix 6-22 | Vivo: ICR mice aged 40 weeks, 0.5mg/ml (replace drinking water) for 20 weeks | Positivel:-; Negative: water | LC3-Ⅱ | Potential increase in mitochondrial autophagy levels in naturally aging ovarian granulosa cells | Unreported | ^164^ |
| 5 | Nobiletin | Activation | *Citrus reticulata* Blanco (*Rutaceae*), *Citrus × aurantium f. deliciosa* (Ten.) M.Hiroe (*Rutaceae*), and other organisms | 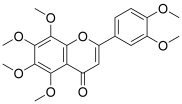 | Vitro: ovarian granulosa cells of hyline white chickens, 10 μg/mL for 72h | Positivel:-; Negative: Dorsomorphin | LC3-Ⅱ, PINK1, Parkin | Potential activation of mitochondrial autophagy via AMPK and SIRT1 pathways, with attenuation of D-galactose-induced mitochondrial damage and delay of apoptosis associated with natural aging | Unreported | ^109^ |
| 6 | Paeoniflorin | Activation | *Paeonia emodi* Royle (*Paeoniaceae*), *Paeonia obovata* Maxim. (*Paeoniaceae*), and other organisms | Shown in Appendix 6-18 | Vivo: ICR mice (3 weeks old), 10 mg/kg/d for 7 weeks via intraperitoneal injection | Positivel:-; Negative: normal saline | LC3-Ⅱ, Beclin-1, p62, PINK1, Parkin | Potential promotion of mitochondrial autophagy to maintain mitochondrial mass in ovarian cells with H2O2-induced oxidative damage | Unreported | ^111^ |
| 7 | Quercetin | Activation | *Allium cepa* L. (*Amaryllidaceae*), *Malus domestica* (Suckow) Borkh. (*Rosaceae*), *Vitis vinifera* L. (*Vitaceae*), and other organisms | Shown in Appendix 6-10 | Vitro: rats ovarian granulosa cells, 20μM for 24h | Positivel:-; Negative: 3-MA | Atg5, Atg12, Atg16L, LC3-Ⅱ/Ⅰ, Beclin-1 | Potential activation of autophagy to mitigate H2O2-induced senescence and damage | Unreported | ^154^ |
| 8 | Resveratrol | Activation | Various plants, including *Vitis vinifera* L. (*Vitaceae*), Reynoutria japonica Houtt. (*Polygonaceae*) | Shown in Appendix 6-6 | Vivo: C57BL/6 mice aged 8 weeks, 50 mg/kg/d gavage every other day for 20 days;  Vitro: mouse primary ovarian granulosa cells, 5μM 24 h | Vivo: Positivel:-; Negative: unknown；  Vitro: Positivel:-; Negative: DMSO | LC3-Ⅱ/Ⅰ, SQSTM1 | Potential promotion of autophagy in leucovorin- and cyclophosphamide-induced ovarian damage | Unreported | ^166^ |
| 9 | Spermidine | Activation | *Glycine max* (L.) Merr. (*Fabaceae*), *Triticum aestivum* L. (*Poaceae*), *Spinacia oleracea* L. (*Amaranthaceae*) and other organisms | 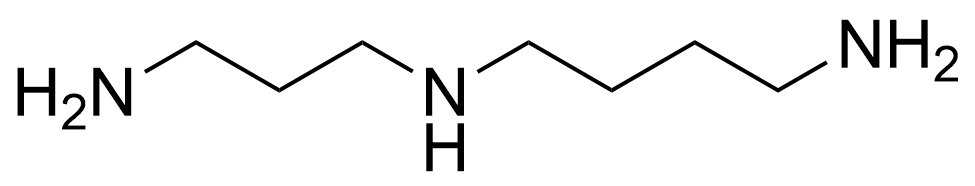 | Vivo: ICR mice aged 7 weeks, 3 mmol/ L for 3 months via p.o | Positivel:-; Negative:normal drinking water | Beclin-1, LC-3II/I, p62 | Potential promotion of autophagy and inhibition of oxidative stress to reduce the number of atretic follicles in naturally aging ovaries | Unreported | ^160^ |
| 10 | Spermidine | Activation | *Glycine max* (L.) Merr. (*Fabaceae*), *Triticum aestivum* L. (*Poaceae*), *Spinacia oleracea* L. (*Amaranthaceae*) and other organisms | See above | Vivo:C57BL/6 J mice aged 6 weeks, 15mg/kg/d for 21 days via intraperitoneal injection; Vitro: porcine ovarian granulosa cells，40 μM for 12 hours | Vivo: Positivel:-; Negative: normal saline; Vitro: Positivel:-; Negative: no treatment | LC3B-II, p62 | Potential restoration of ovarian reserve in a 3-NPA/H2O2-induced oxidative stress model to improve fertility | Unreported | ^161^ |
| 11 | Allantoin isolated from *Dioscorea oppositifolia* L. | Inhibition | *Dioscorea oppositifolia* L. (*Dioscoreaceae*) | Shown in Appendix 6-1 | Vivo: Sprague-Dawley rats，140 mg/kg/d for 3 weeks | Positivel:estradiol valerate; Negative:distilled water | LC3B-II/I | Potential attenuation of ovarian function and inhibition of mitochondrial autophagy in granulosa cells in rats with cyclophosphamide-induced premature ovarian failure | Unreported | ^85^ |
| 12 | Curculigoside | Inhibition | *Curculigo sinensis* S.C.Chen (*Hypoxidaceae*), *Degeneria vitiensis* I.W.Bailey & A.C.Sm. (*Degeneriaceae*), and other organisms | 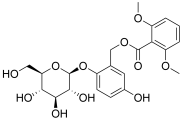 | Vivo: Balb/c mice aged 6-8 weeks, 40 mg/kg/d for 28 days via gavage | Positivel:-; Negative: normal saline | Beclin-1, LC3-Ⅱ/Ⅰ，p62 | Potential inhibition of autophagy in ovarian tissue and alleviation of oxidative stress to improve ovarian reserve | Unreported | ^167^ |
| 13 | Dehydroepiandrosterone | Inhibition | synthesized in the adrenal glands in *Homo sapiens;* plant sources, such as *Dioscorea villosa* L. (*Dioscoreaceae*) | Shown in Appendix 6-8 | Vivo: Sprague-Dawley rats aged 3 months, 60 mg/kg/d for 21 days via subcutaneously injection | Positivel:-; Negative: sesame oil | Pink 1, Parkin, LC3, AMPK, SIRT1 | Potential inhibition of deoxyvinylcyclohexene-induced autophagy in rat ovarian cells in the DOR model and potential improvement of ovarian reserve | Unreported | ^168^ |
| 14 | Procyanidins | Inhibition | *Lathyrus laxiflorus* (Desf.) Kuntze (*Fabaceae*), *Vitis amurensis* Rupr. (*Vitaceae*), and other organisms | Mixture of compounds, structures vary. | Vitro: ovarian granulosa cells of D280 /D580 hens, 10μM, duration of action unknown | Positivel:-; Negative: normal saline | LC3B, SQSTM1 | Potential inhibition of autophagy in naturally senescent granulosa cells and inhibition of H2O2-induced autophagy | Unreported | ^169^ |
| 3 | Melatonin | Inhibition | synthesized in the pineal gland in *Homo sapiens;* plant sources, such as *Prunus cerasus* L. (*Rosaceae*) | Shown in Appendix 6-15 | Vivo: neonatal CD-1 mice, 1 μM intraperitoneally for 5 days; Vitro: neonatal CD-1 mice ovaries, 1 μM for 5 days | Positivel:-; Negative: normal saline | Beclin 1, LC-3II/I, AMPKα-1, AKT, mTOR | Potential inhibition of ovarian autophagy and protection of ovarian reserve in nicotine-exposed mice | Unreported | ^170^ |
| 3 | Melatonin | Inhibition | synthesized in the pineal gland in *Homo sapiens;* plant sources, such as *Prunus cerasus* L. (*Rosaceae*) | Shown in Appendix 6-15 | Vitro: KGN cells, 100 pM for 24 hours | Positivel:-; Negative: PBS | Beclin-1, LC-3II/I, p62 | Potential regulation of the miR-15a-5p/STAT3 axis to activate the PI3K/Akt/mTOR pathway and inhibit serum starvation-induced autophagy in POI model cells | Unreported | ^171^ |
| 3 | Melatonin | Inhibition | synthesized in the pineal gland in *Homo sapiens;* plant sources, such as *Prunus cerasus* L. (*Rosaceae*) | Shown in Appendix 6-15 | Vitro: KGN cells, 100 pM for 24 hours | Positivel:-; Negative:no treatment | LC3-Ⅱ/Ⅰ, Agt5 | Potential activation of the PI3K/Akt/mTOR pathway and inhibition of serum starvation-induced autophagy in POI model cells | Unreported | ^172^ |
| 3 | Melatonin | Inhibition | synthesized in the pineal gland in *Homo sapiens;* plant sources, such as *Prunus cerasus* L. (*Rosaceae*) | Shown in Appendix 6-15 | Vitro: Chinese hamster ovary cells, 10^-9^ M, duration of action unknown | Positivel:-; Negative:no treatment | SDHA, mitofilin, OPA1, MFN2, DRP 1 | Potential rescue of excessive autophagy-induced damage to mitochondrial number and membrane potential in cells | Unreported | ^173^ |
| 3 | Melatonin | Inhibition | synthesized in the pineal gland in *Homo sapiens;* plant sources, such as *Prunus cerasus* L. (*Rosaceae*) | Shown in Appendix 6-15 | Vivo: C57 BL/6 J mice aged 6-8 weeks, dose, form, time of administration unknown | Positivel:-; Negative:no treatment | LC3B | Potential inhibition of the eIF2α/ATF4 pathway and suppression of cyclophosphamide-induced autophagy and apoptosis in ovarian cells | Unreported | ^107^ |

Note: Only representative structures with pharmacological significance or structural complexity are shown; a full list is provided in Supplementary Appendix 6. Therapeutic effects listed are based on experimental models. In vitro-only data do not indicate clinical efficacy. In vivo findings are preliminary and require further validation.
