# Supplementary material for: Targeting programmed cell death with natural products: a potential therapeutic strategy for diminished ovarian reserve and fertility preservation
Source: Front Pharmacol. 2025 May 29;16:1546041. doi: 10.3389/fphar.2025.1546041 (PMC12158948; doi:10.3389/fphar.2025.1546041)
Supplement: Supplementary file 3 [file Table5.docx]

Appendix 5 Therapeutic potential of natural products in the treatment of DOR: targeting necroptosis

| No. | Natural products | Source | Structure | Optimal dose | Control | Necroptosis-related targets | Potential effect | Adverse effects | References |
| --- | --- | --- | --- | --- | --- | --- | --- | --- | --- |
| 1 | Scutellarin | *Perilla frutescens* (L.) Britton (*[Lamiaceae](https://powo.science.kew.org/taxon/urn:lsid:ipni.org:names:30000097-2)*  ), *Scutellaria indica* L. (*Lamiaceae*), and other organisms | Shown in Appendix 6-20 | Vitro: Mouse primary granulosa cells, 2000 μg/mL for 24 hours | Positivel:-; Negative: DMSO | Specifics not available | Potential attenuation of ZEA-induced cell damage | Elevated RIPK1 expression | ^115,255^ |
| 2 | Dehydroepiandrosterone | synthesized in the adrenal glands in *Homo sapiens;* plant sources, such as *Dioscorea villosa* L. (*Dioscoreaceae*) | Shown in Appendix 6-8 | Vitro: human GC cell line HO-23, 1 nM for 20 hours | Positivel: 5%FBS; Negative: staurosporine | RIPK1, RIPK3 | Potential inhibition of serum starvation-induced necrotic apoptosis | Unreported | ^96^ |

Note: A full list is provided in Supplementary Appendix 6. Potential effects listed are based on experimental models. In vitro-only data do not indicate clinical efficacy. In vivo findings are preliminary and require further validation.
