# Supplementary material for: Targeting programmed cell death with natural products: a potential therapeutic strategy for diminished ovarian reserve and fertility preservation
Source: Front Pharmacol. 2025 May 29;16:1546041. doi: 10.3389/fphar.2025.1546041 (PMC12158948; doi:10.3389/fphar.2025.1546041)
Supplement: Supplementary file 4 [file Table3.docx]

Appendix 3 Therapeutic potential of natural products in the treatment of DOR: targeting ferroptosis

| No. | Natural products | Source | Structure | Optimal dose | Control | Ferroptosis-related targets | Potential effect | Adverse effects | References |
| --- | --- | --- | --- | --- | --- | --- | --- | --- | --- |
| 1 | Berberine | *Coptis chinensis* Franch. (*Ranunculaceae*), *Phellodendron amurense* Rupr. (*Rutaceae*), *Pinellia ternata* (Thunb.) Makino (*Araceae*), and other organisms | Shown in Appendix 6-25 | Vivo: C3He-ATP7B^tx-j^ mice aged 8-10 weeks，0.15g/kg/d for 3 weeks via gavage | Positivel:-; Negative: normal saline | GSSG, PTGS2, GSH, GPX4 | Potential inhibition of ferroptosis in ovarian tissue and improvement of ovarian reserve | Unreported | ^201^ |
| 2 | Icariin | *Epimedium brevicornu* Maxim. (*Berberidaceae*), *Epimedium truncatum* H.R.Liang (*Berberidaceae*), and other organisms | 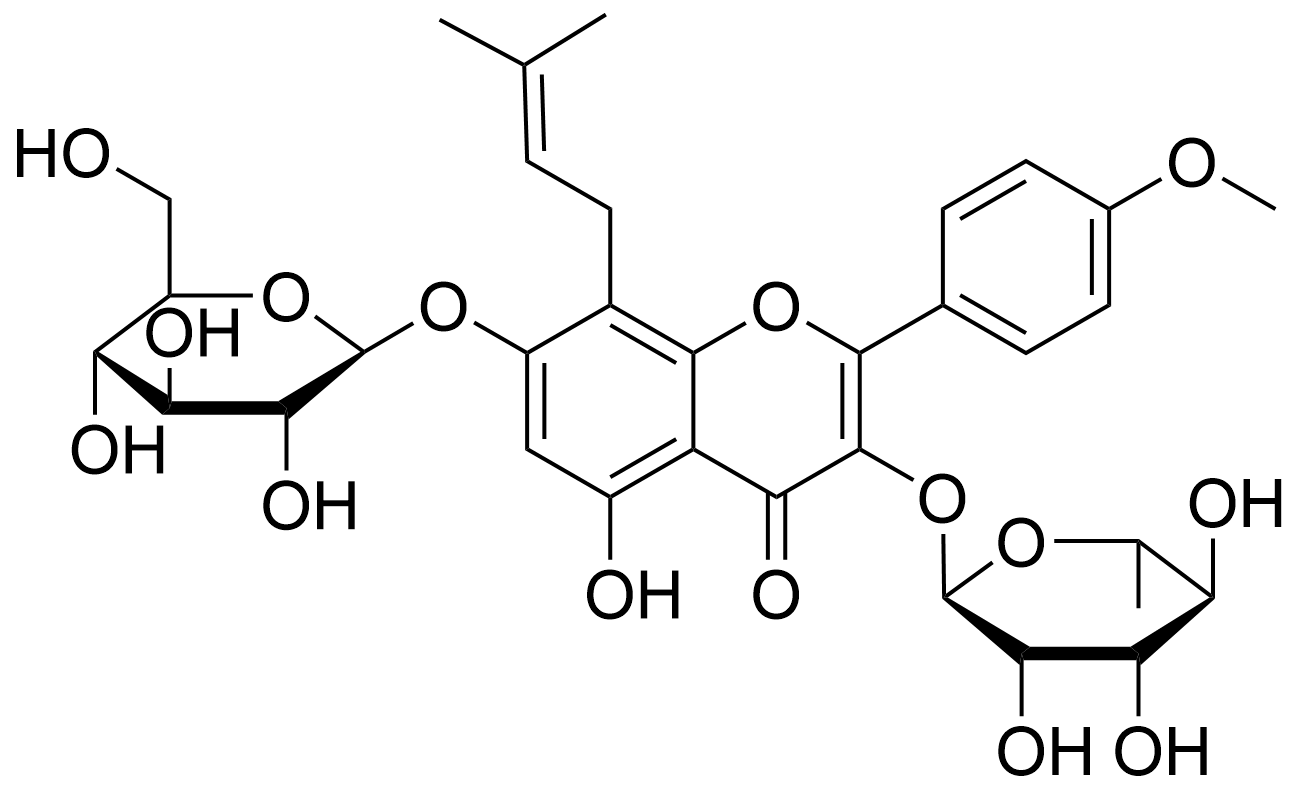 | Vivo: ICR mice aged 6-8 weeks, 30 mg/kg/d for 21days via intraperitoneal injection ；Vitro：KGN cells，5μg/ml for 6 hours | Positivel:-; Negative: DMSO | Nrf 2, HO-1, GPX 4 | Potential reduction of oxidative stress and ferroptosis via the Nrf2/ARE pathway, and protection of ovarian tissue and KGN cells from cisplatin-induced injury | Unreported | ^106^ |
| 3 | Pterostilbene | *Vitis riparia* Michx. (*Vitaceae*), *Vitis vulpina* L. (*Vitaceae*), and other organism | Shown in Appendix 6-26 | Vitro: COV 434 and KGN cells, 20 μM for 6 hours | Positivel:-; Negative: no treatment | GSH, ACSL4, Nrf2, HO-1, GPX4 | Potential inhibition of H2O2-induced ferroptosis, attenuation of oxidative stress, and improvement of cell viability | Unreported | ^198^ |
| 4 | Rutin | *Camellia sinensis* (L.) Kuntze (*Theaceae*), *Amaranthus hybridus* L. (*Amaranthaceae*), and other organisms | Shown in Appendix 6-27 | Vitro：small white follicles from 580-day-old (D580) laying chicken, 0.2 μM for 24 hours | Positivel: dimethyl fumarate; Negative: ML385 | Nrf 2, HO-1 | Potential attenuation of ferroptosis through the Nrf2/HO-1 pathway | Unreported | ^197^ |
| 5 | Spermidine | *Glycine max* (L.) Merr. (*Fabaceae*), *Triticum aestivum* L. (*Poaceae*), *Spinacia oleracea* L. (*Amaranthaceae*) and other organisms | 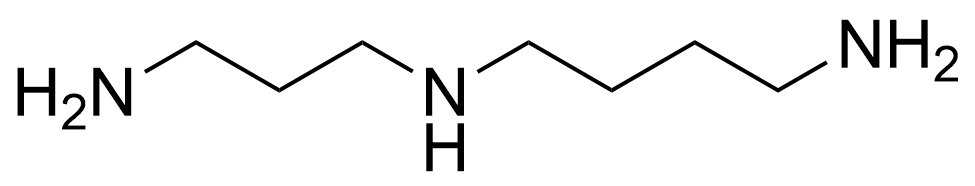 | Vivo：C57 BL/6J mice aged 6weeks，10mg/kg/d for 21 days via intraperitoneal injection ；Vitro：porcine ovarian granulosa cells，40 μM for 12 hours | Vivo: Positivel:-; Negative: normal saline; Vitro: Positivel:-; Negative: no treatment | GSH-Px, GPX4, p-Nrf2, HO-1, FHC, ACSL4 | Potential attenuation of 3-NPA-induced oxidative damage, restoration of ovarian reserve, and improvement of fertility in mice through activation of the Nrf2/HO-1/GPX4 pathway; potential inhibition of ferroptosis in porcine ovarian granulosa cells via the Akt/FHC/ACSL4 pathway | Unreported | ^161^ |
| 6 | Sphingosine-1-phosphate | synthesized within cells (including immune cells, endothelial cells, and neurons) through the action of sphingosine kinase | Shown in Appendix 6-28 | Vitro：KGN cells，10 μM for 2 hours | Positivel: deferoxamine; Negative: no treatment | GPX4, FPN1, TFRC | Potential reduction of radiation-induced ferroptosis | Unreported | ^192^ |

Note: Only representative structures with pharmacological significance or structural complexity are shown; a full list is provided in Supplementary Appendix 6. Potential effects listed are based on experimental models. In vitro-only data do not indicate clinical efficacy. In vivo findings are preliminary and require further validation.
