# Supplementary material for: Targeting programmed cell death with natural products: a potential therapeutic strategy for diminished ovarian reserve and fertility preservation
Source: Front Pharmacol. 2025 May 29;16:1546041. doi: 10.3389/fphar.2025.1546041 (PMC12158948; doi:10.3389/fphar.2025.1546041)
Supplement: Supplementary file 5 [file Table4.docx]

Appendix 4 Therapeutic potential of natural products in the treatment of DOR: targeting pyroptosis

| No. | Natural products | Source | Structure | Optimal dose | Control | Pyroptosis-related targets | Potential effect | Adverse effects | References |
| --- | --- | --- | --- | --- | --- | --- | --- | --- | --- |
| 1 | α-ketoglutarate | a naturally occurring compound found in the Krebs cycle | 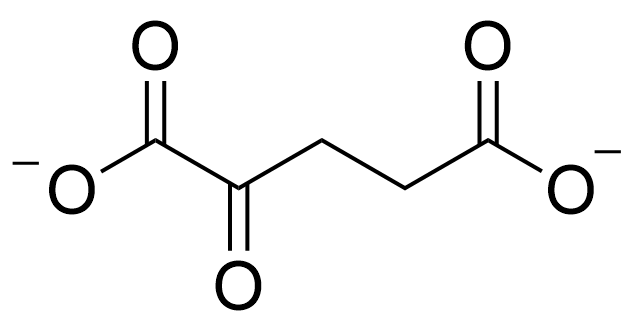 | Vivo: Sprague Dawley rats aged 8 weeks, 250 mg/kg/d for 42 hours ; Vitro: KGN cells, 2nM for 24 hours | Vivo: Positivel:-; Negative: normal saline；  Vitro: Positivel: MCC 950；  Negative:no treatment | NLRP 3, GSDMD, Caspase-1, IL-18, IL-1β | Potential improvement of ovarian reserve, increased lactate levels, and upregulation of glycolysis-limiting enzymes in the ovaries of rats in a cyclophosphamide-induced POI model *in vivo*; potential inhibition of LPS- and Nigerian bacteriocin-induced pyroptosis and restoration of the glycolytic process *in vitro* | Unreported | ^218^ |
| 2 | Allantoin isolated from *Dioscorea oppositifolia* L. | *Dioscorea oppositifolia* L. (*Dioscoreaceae*) | Shown in Appendix 6-1 | Vivo: Sprague-Dawley rats, 140 mg/kg/d for 3 weeks | Positivel:estradiol valerate; Negative:distilled water | NLRP 3, GSDMD, Caspase-1, IL-1β | Potential improvement of ovarian reserve in rats with a cyclophosphamide-induced POI model | Elevated NLRP3 expression | ^85^ |
| 3 | Coenzyme Q10 | primarily synthesized in the liver within the *Homo sapiens*；plants such as *Spinacia oleracea* L. (*Amaranthaceae*) , *Brassica oleracea* L. (*Brassicaceae*  ), *Daucus carota* L. (*Apiaceae*) | Shown in Appendix 6-29 | Vivo: C57BL/6 mice aged 6-8 weeks, 1.25 mg/kg/d for 4 weeks via gavage | Positivel:-; Negative: normal saline | NLRP 3, GSDMD, Caspase-1, IL-1β | Potential inhibition of cyclophosphamide-induced mitochondrial dysfunction and activation of mitochondrial biosynthesis to protect ovarian reserve function | Unreported | ^24^ |
| 4 | Leonurine hydrochloride | *Leonurus japonicus* Houtt. (*Lamiaceae*) | Shown in Appendix 6-30 | Vivo：ICE mice, 30 mg/kg/d for 28 days via i.p | Positivel:-; Negative: normal saline | NLRP 3, GSDMD, Caspase-1, IL-18, IL-1β | Potential resistance to cyclophosphamide-induced ovarian damage | Unreported | ^218^ |
| 5 | Quercetin | *Allium cepa* L. (*Amaryllidaceae*), *Malus domestica* (Suckow) Borkh. (*Rosaceae*), *Vitis vinifera* L. (*Vitaceae*), and other organisms | Shown in Appendix 6-20 | Vivo：C57BL/6 mice aged 6-8 weeks, 50 mg/kg/d for 4 weeks via gavage | Positivel:-; Negative: normal saline | NLRP 3, GSDMD, Caspase-1, IL-1β | Potential inhibition of cyclophosphamide-induced mitochondrial dysfunction and activation of mitochondrial biosynthesis to protect ovarian reserve function | Unreported | ^85^ |

Note: Only representative structures with pharmacological significance or structural complexity are shown; a full list is provided in Supplementary Appendix 6. Potential effects listed are based on experimental models. In vitro-only data do not indicate clinical efficacy. In vivo findings are preliminary and require further validation.
