# Supplementary material for: Targeting programmed cell death with natural products: a potential therapeutic strategy for diminished ovarian reserve and fertility preservation
Source: Front Pharmacol. 2025 May 29;16:1546041. doi: 10.3389/fphar.2025.1546041 (PMC12158948; doi:10.3389/fphar.2025.1546041)
Supplement: Supplementary file 6 [file Table6.docx]

Appendix 6 Chemical line drawings

| No. | Natural products | Structure |
| --- | --- | --- |
| 1 | Allantoin isolated from *Dioscorea oppositifolia* L. | 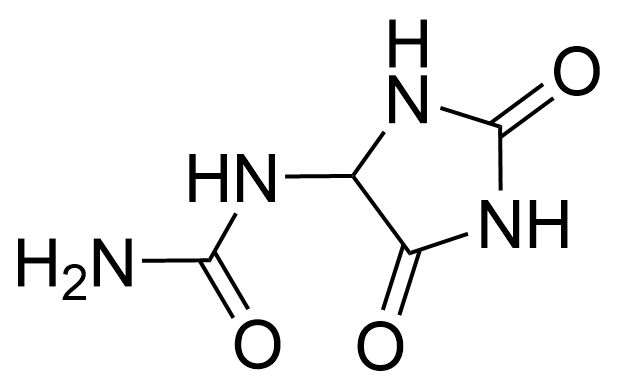 |
| 2 | α-ketoglutarate | 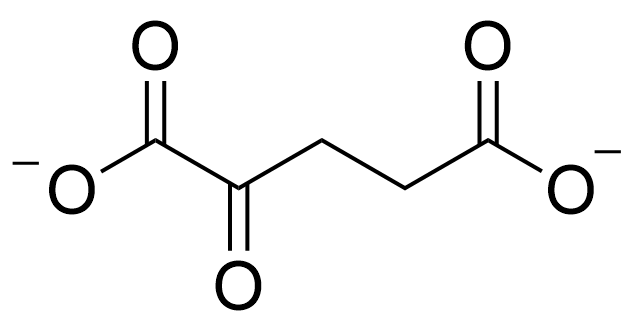 |
| 3 | Apigenin | 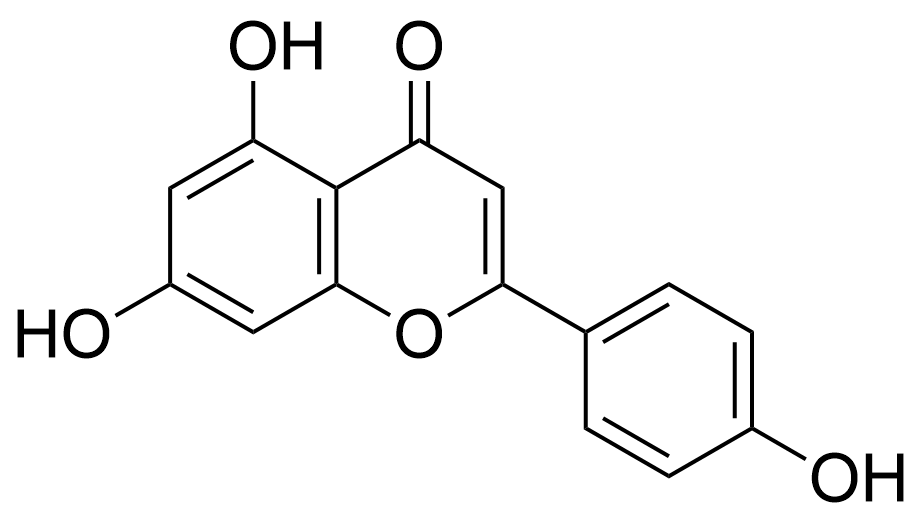 |
| 4 | Capsaicin | 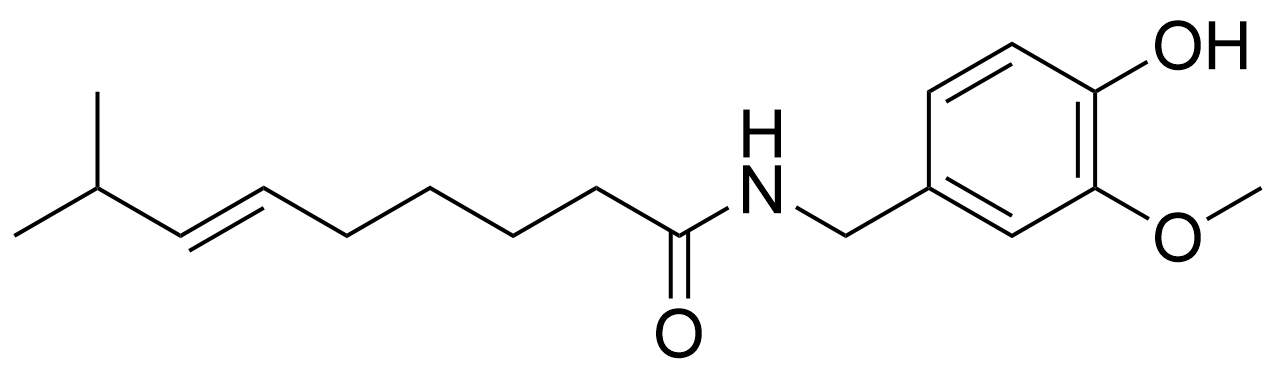 |
| 5 | Chrysin | 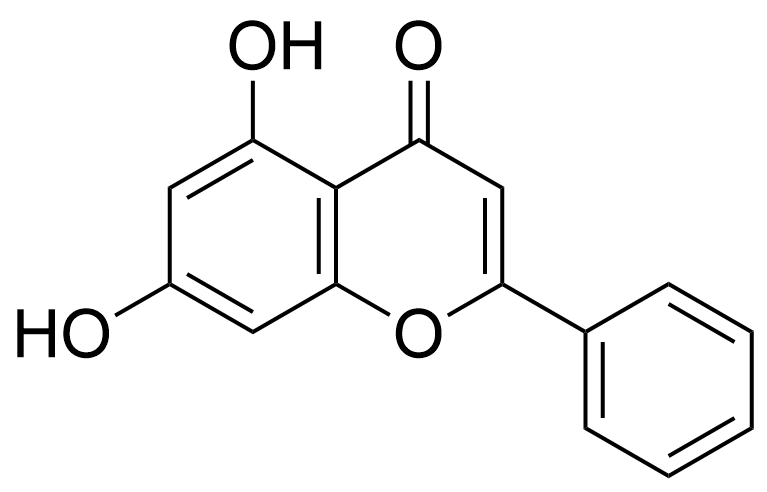 |
| 6 | Resveratrol | 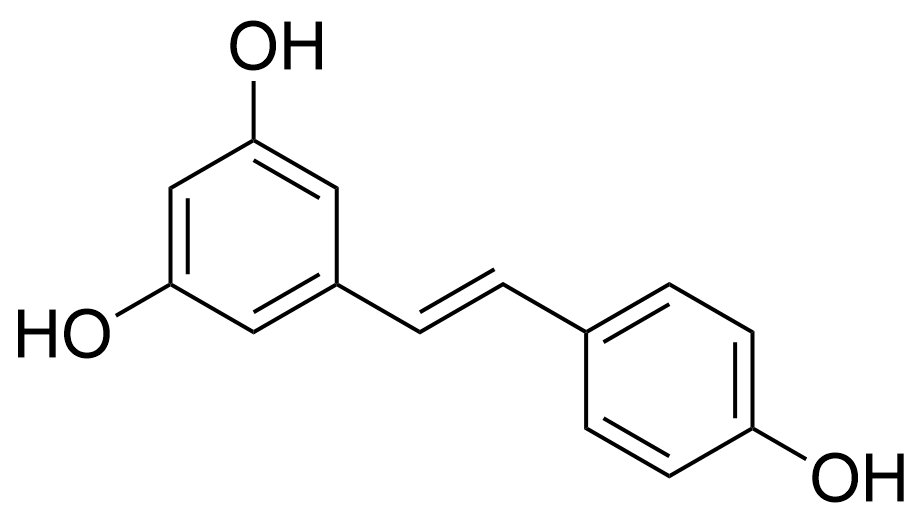 |
| 7 | Curcumin | 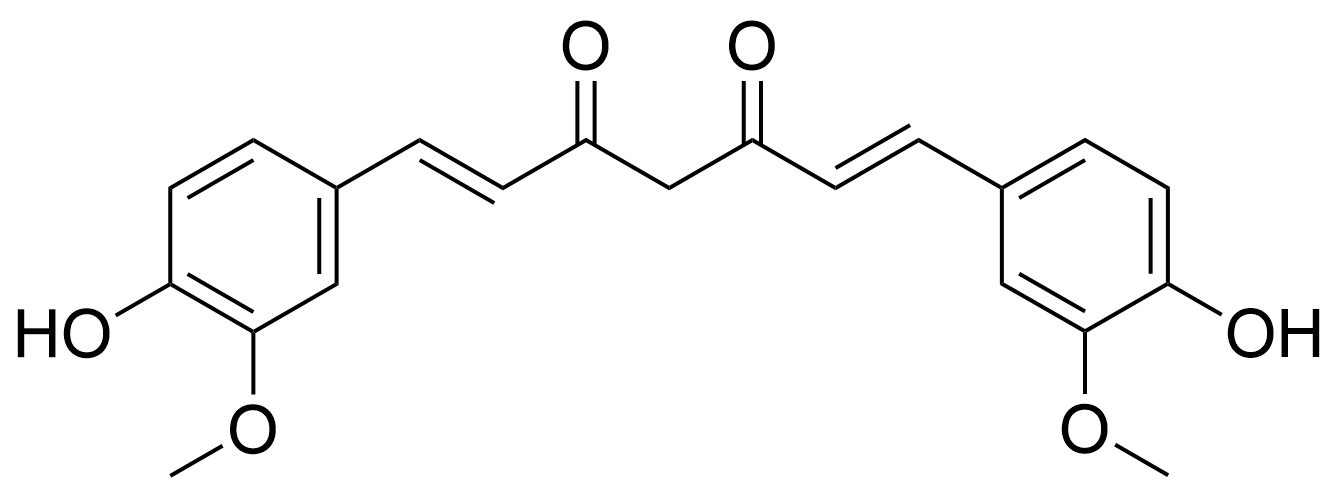 |
| 8 | Dehydroepiandrosterone | 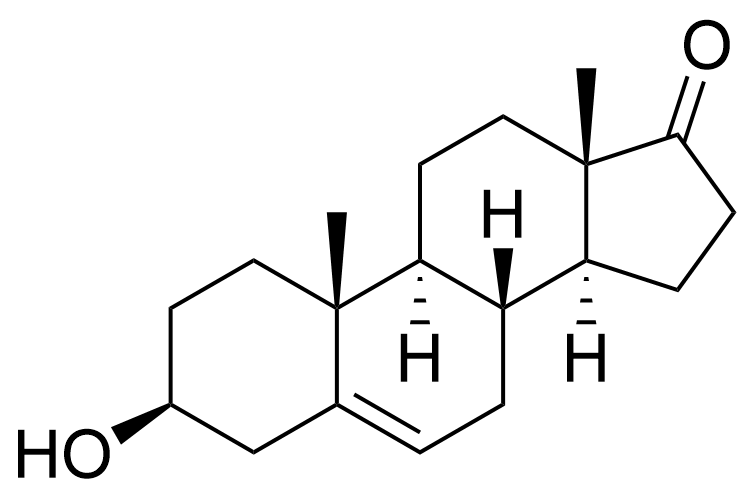 |
| 9 | Epigallocatechin-3-gallate | 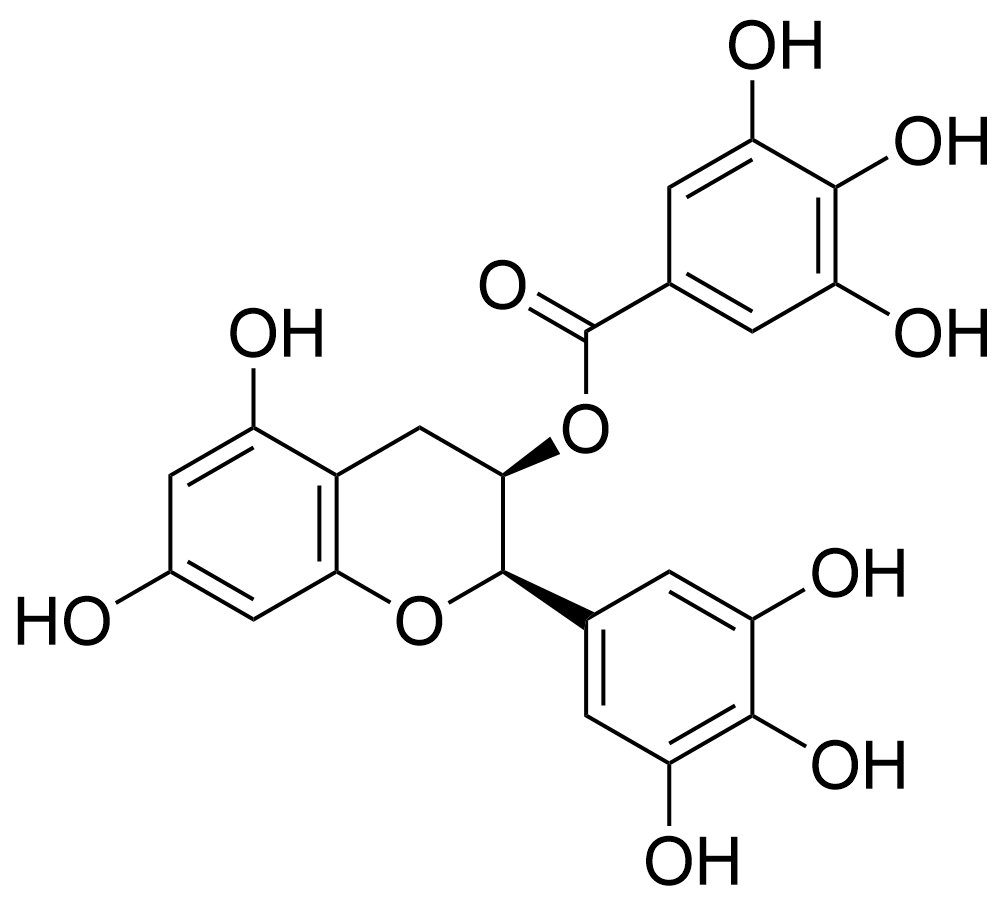 |
| 10 | Quercetin | 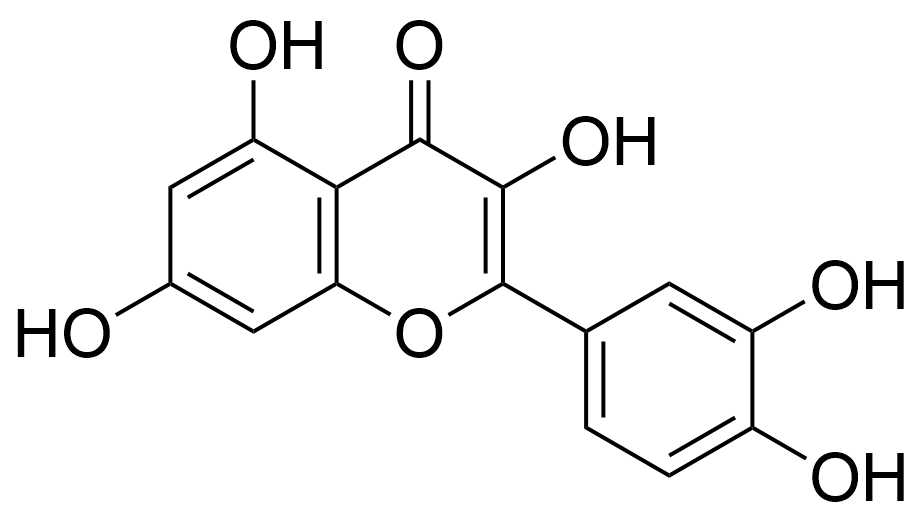 |
| 11 | Diosgenin | 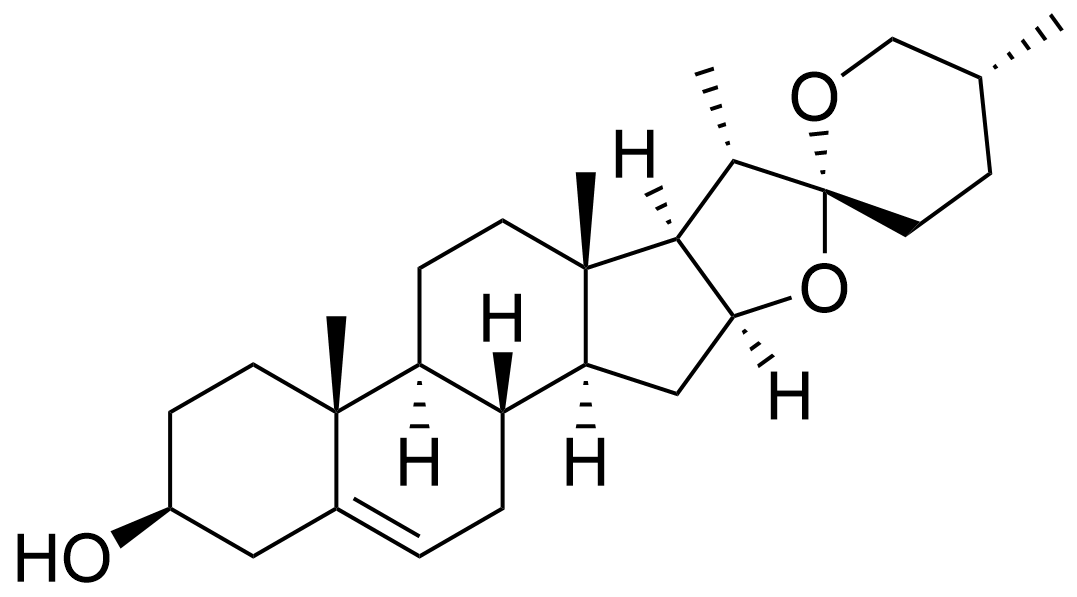 |
| 12 | Eugenol | 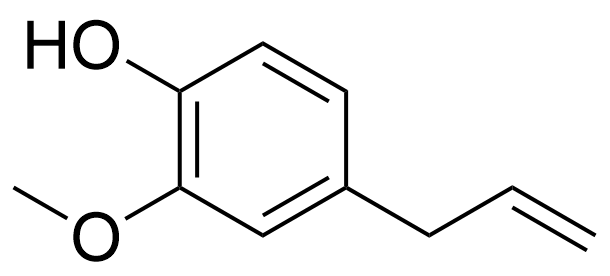 |
| 13 | Procyanidin B2 | 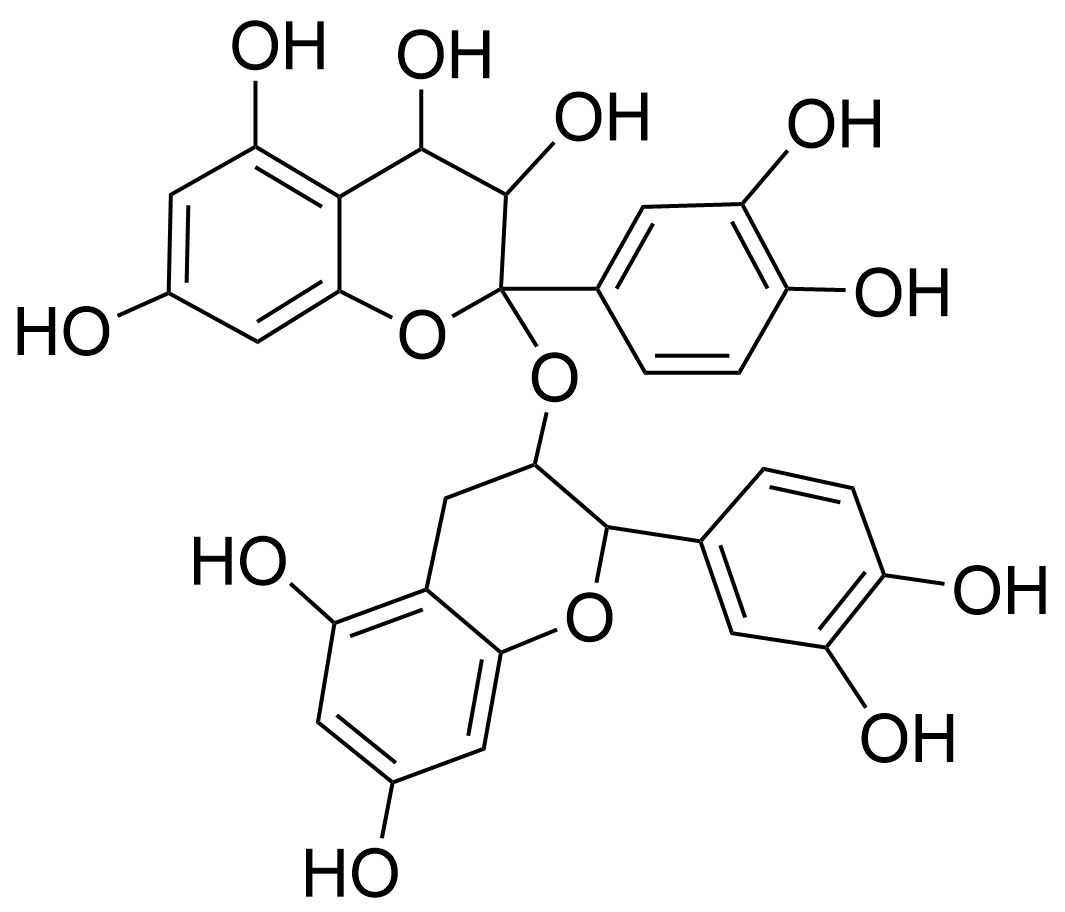 |
| 14 | Honokiol | 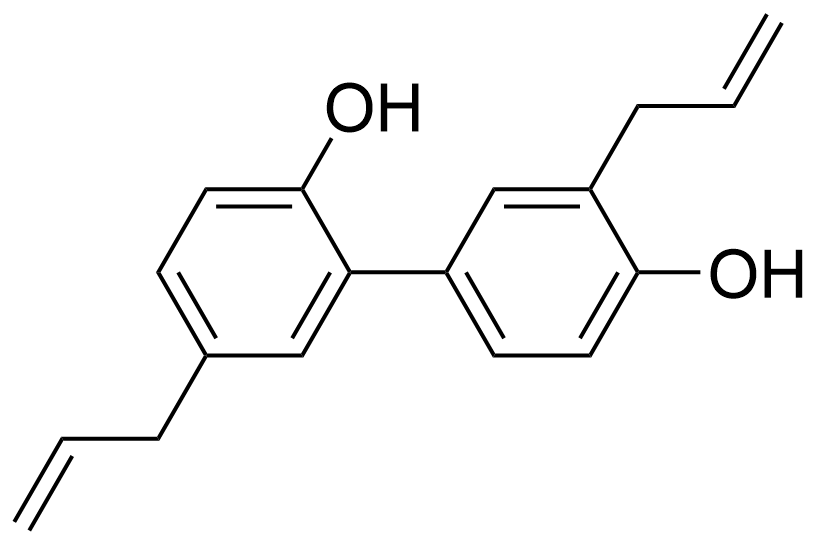 |
| 15 | Icariin | 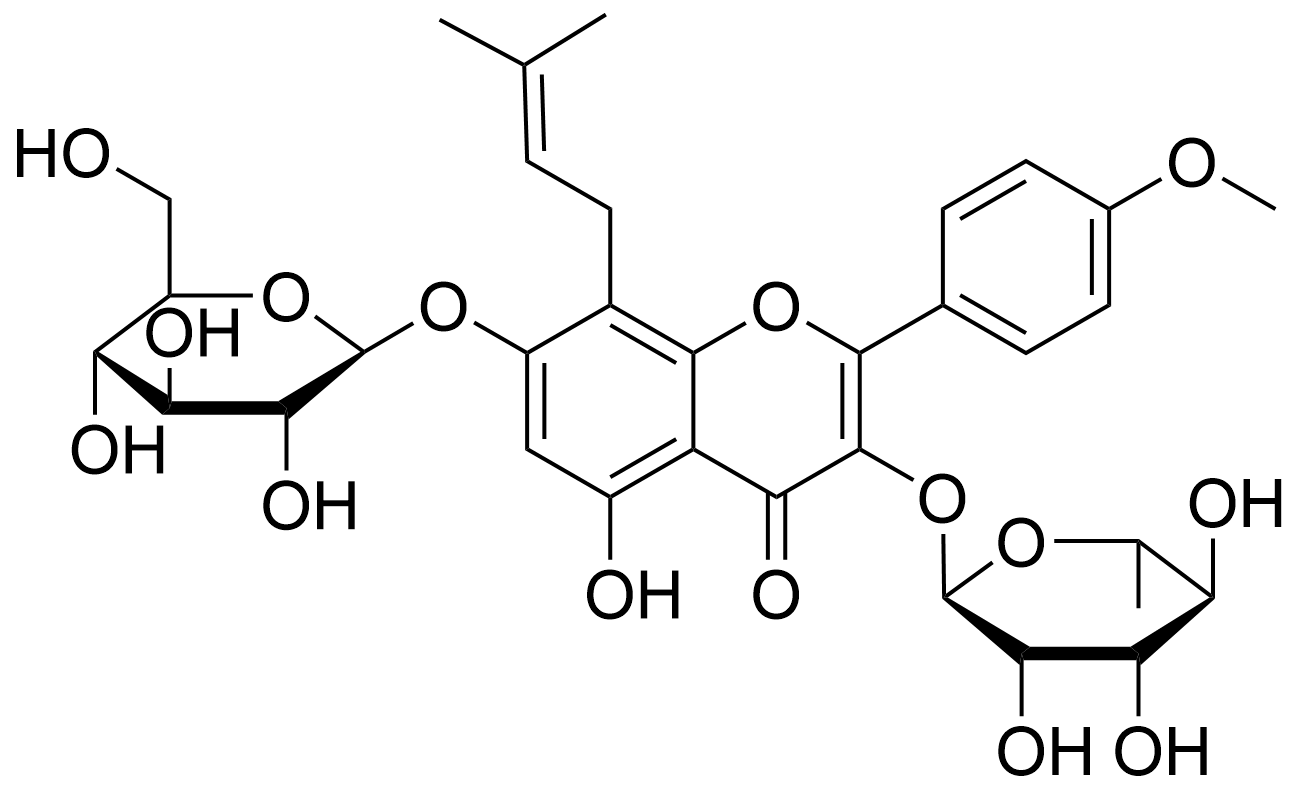 |
| 16 | Melatonin | 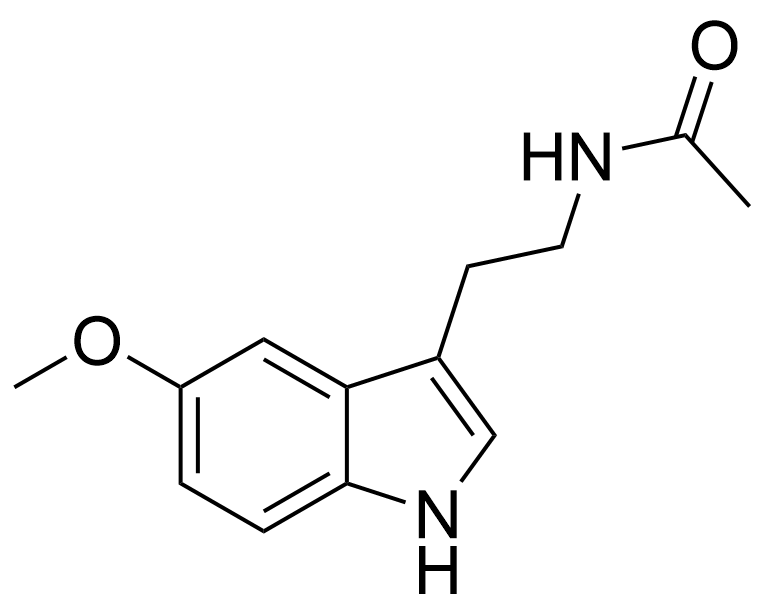 |
| 17 | Nobiletin | 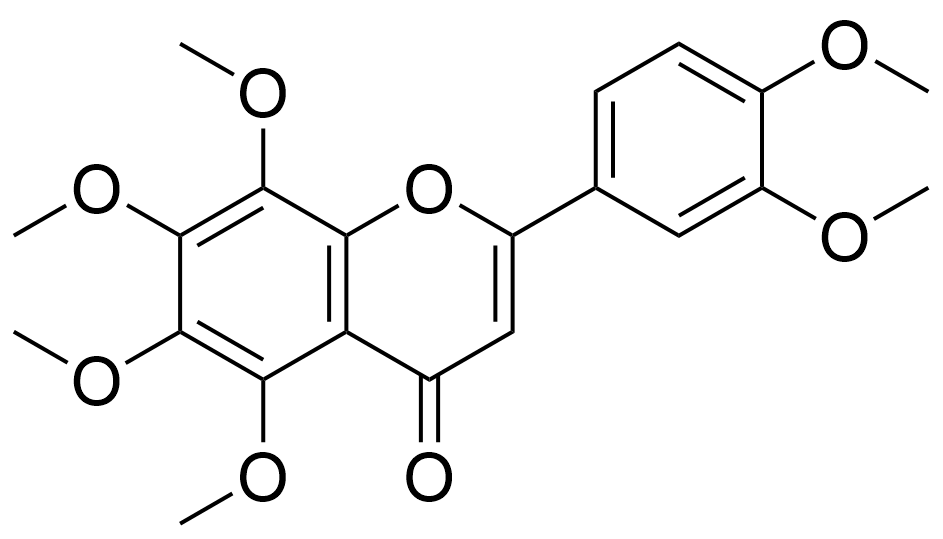 |
| 18 | Paeoniflorin | 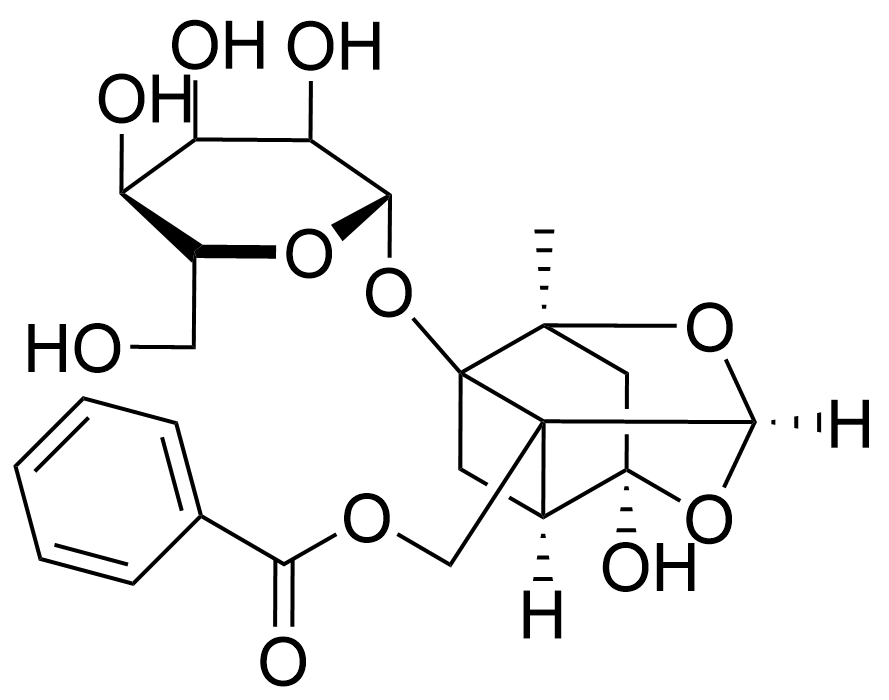 |
| 19 | Puerarin | 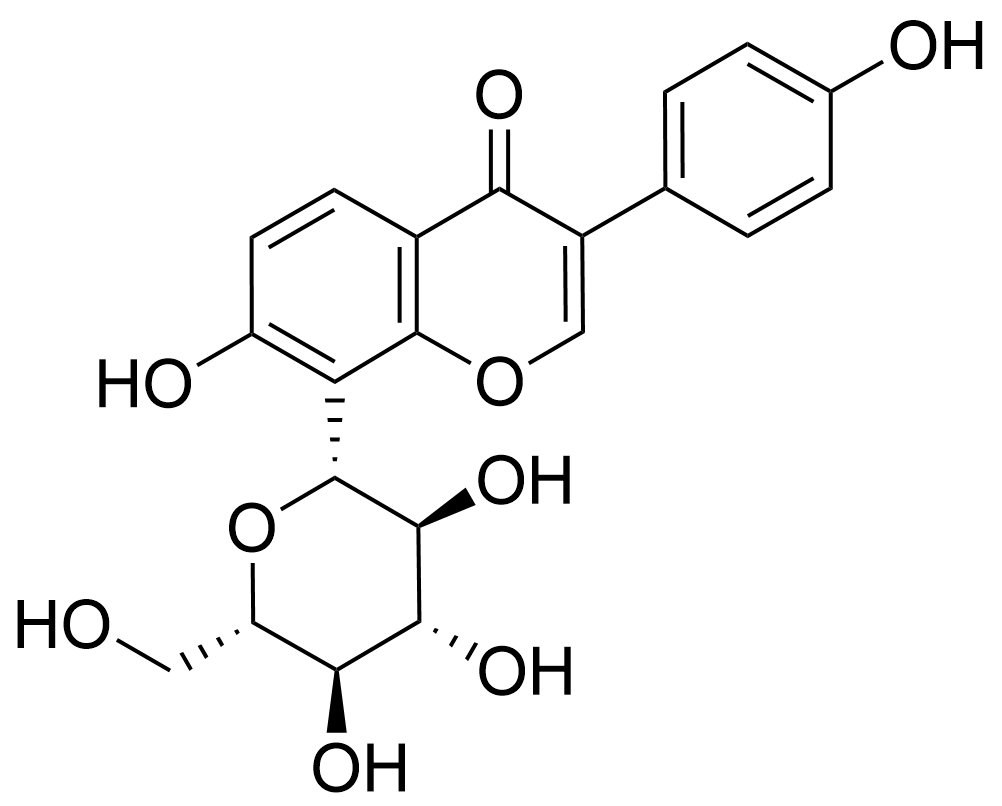 |
| 20 | Scutellarin | 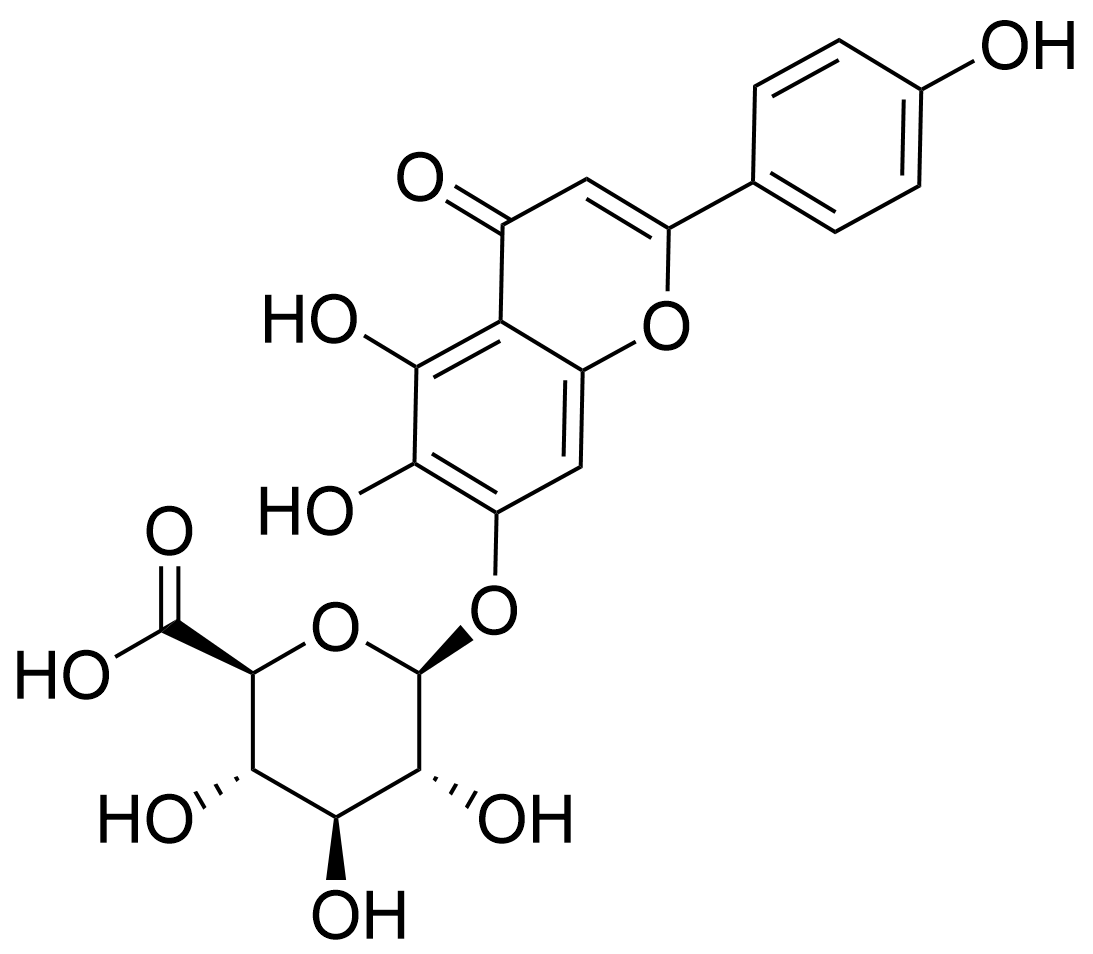 |
| 21 | Tannic acid | 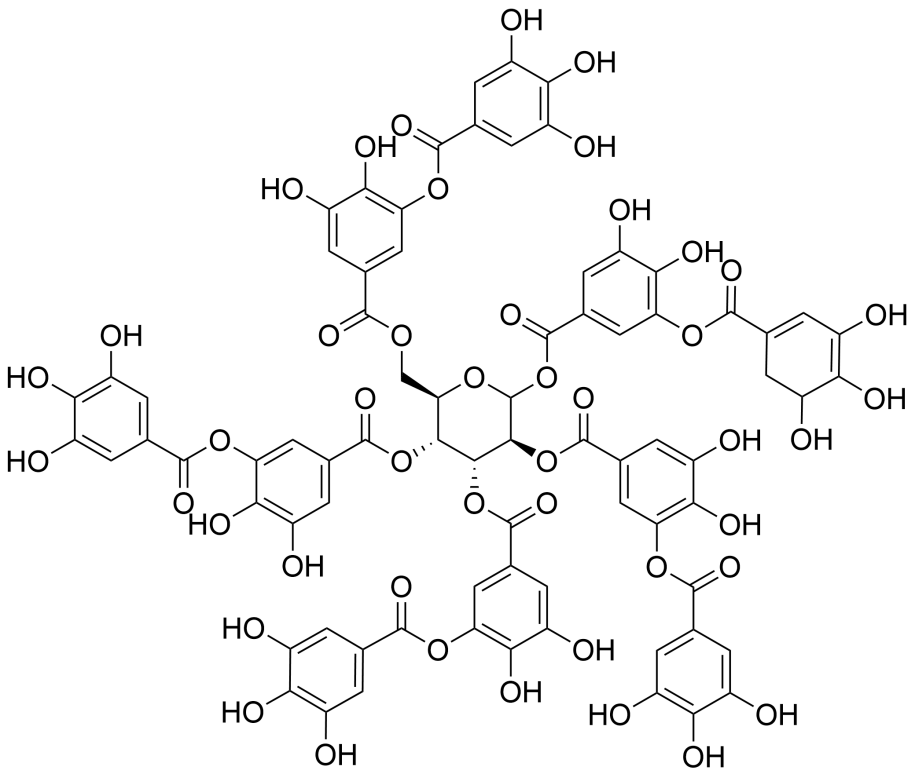 |
| 22 | Nicotinamide mononucleotide | 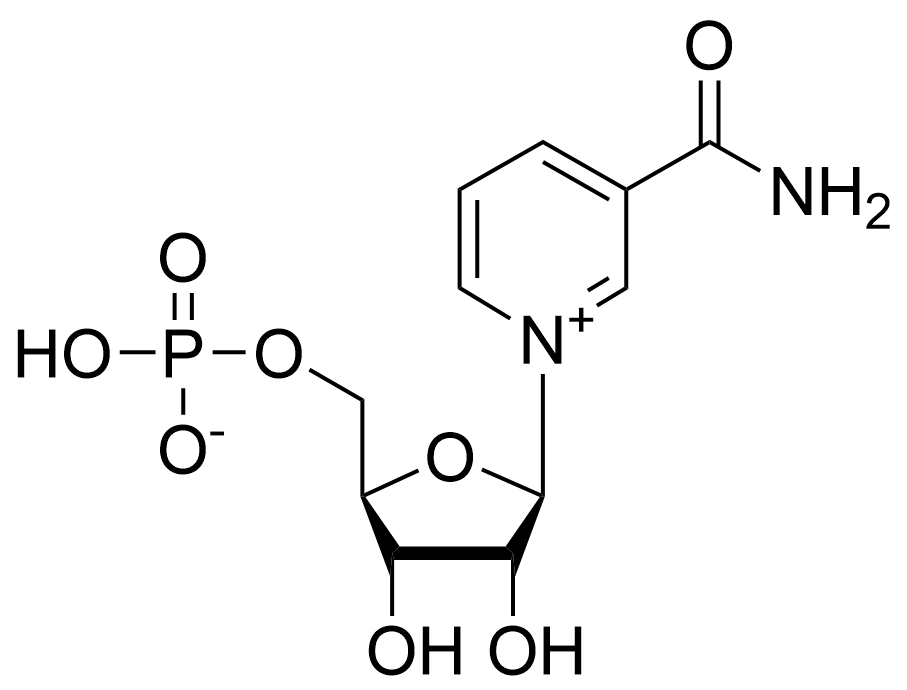 |
| 23 | Spermidine | 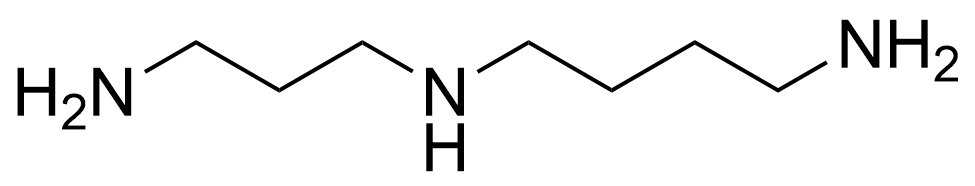 |
| 24 | Curculigoside | 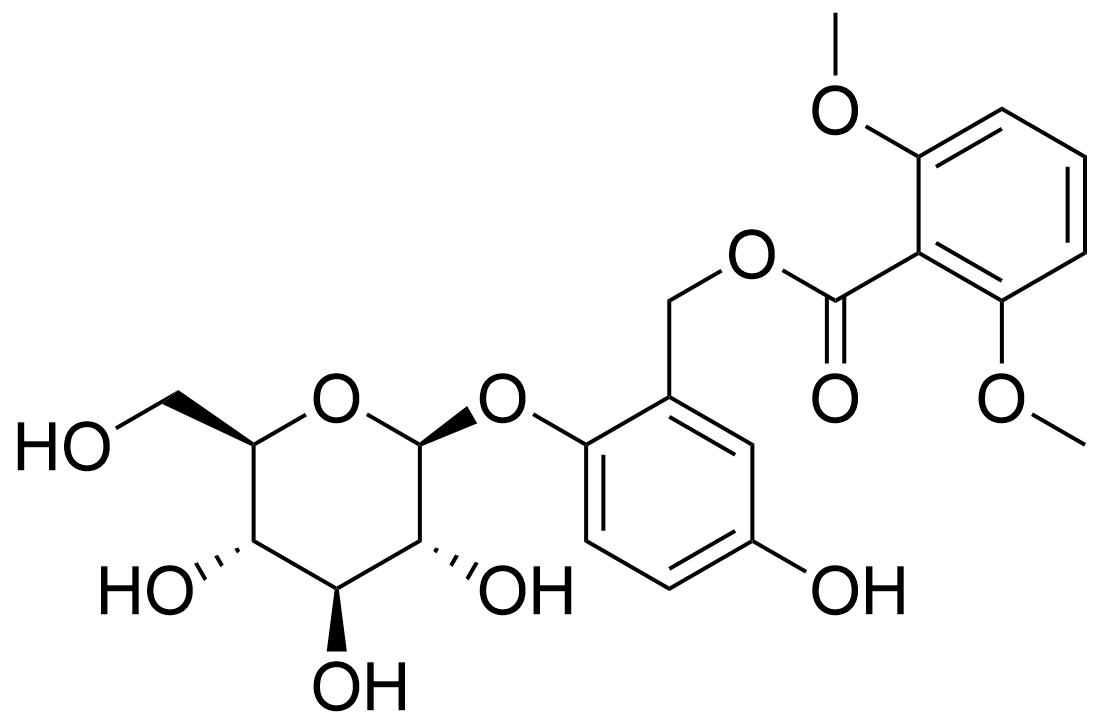 |
| 25 | Berberine | 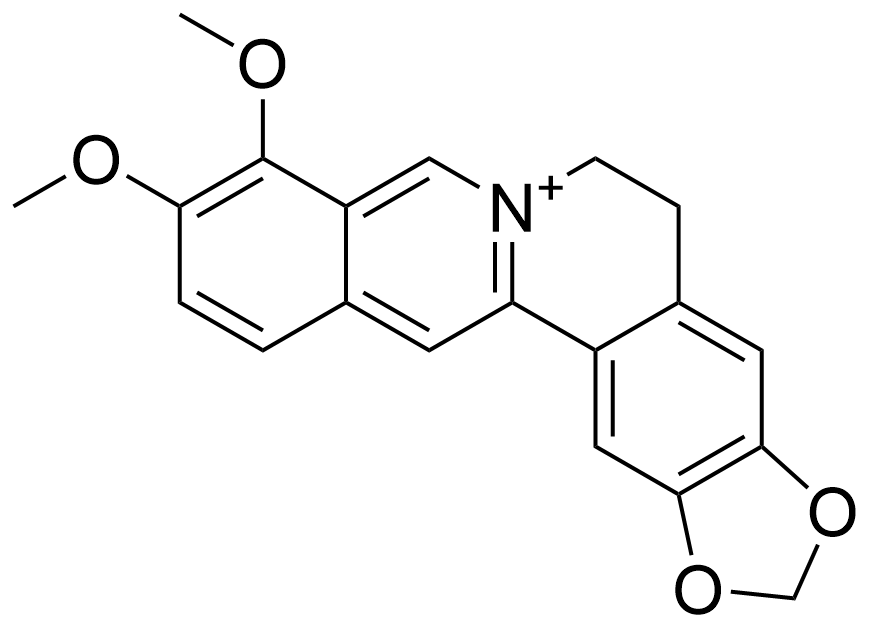 |
| 26 | Pterostilbene | 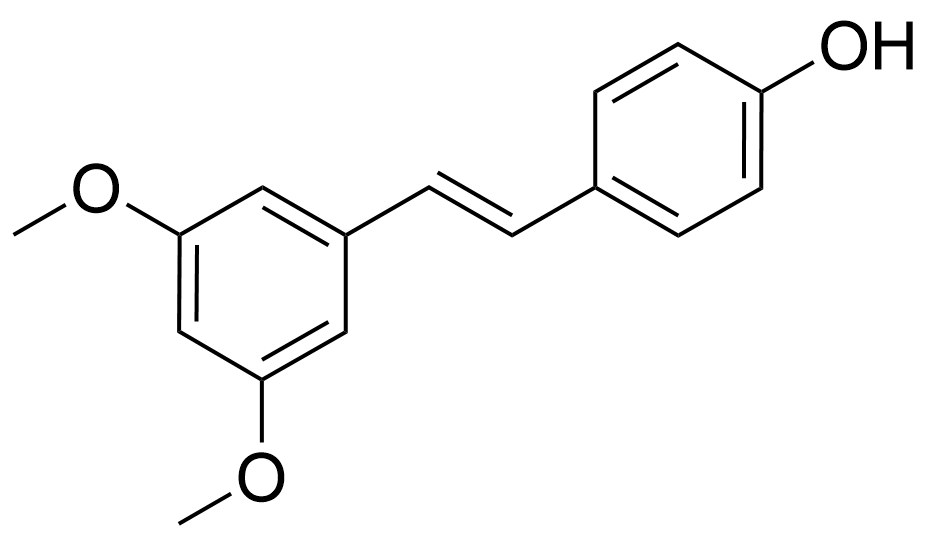 |
| 27 | Rutin | 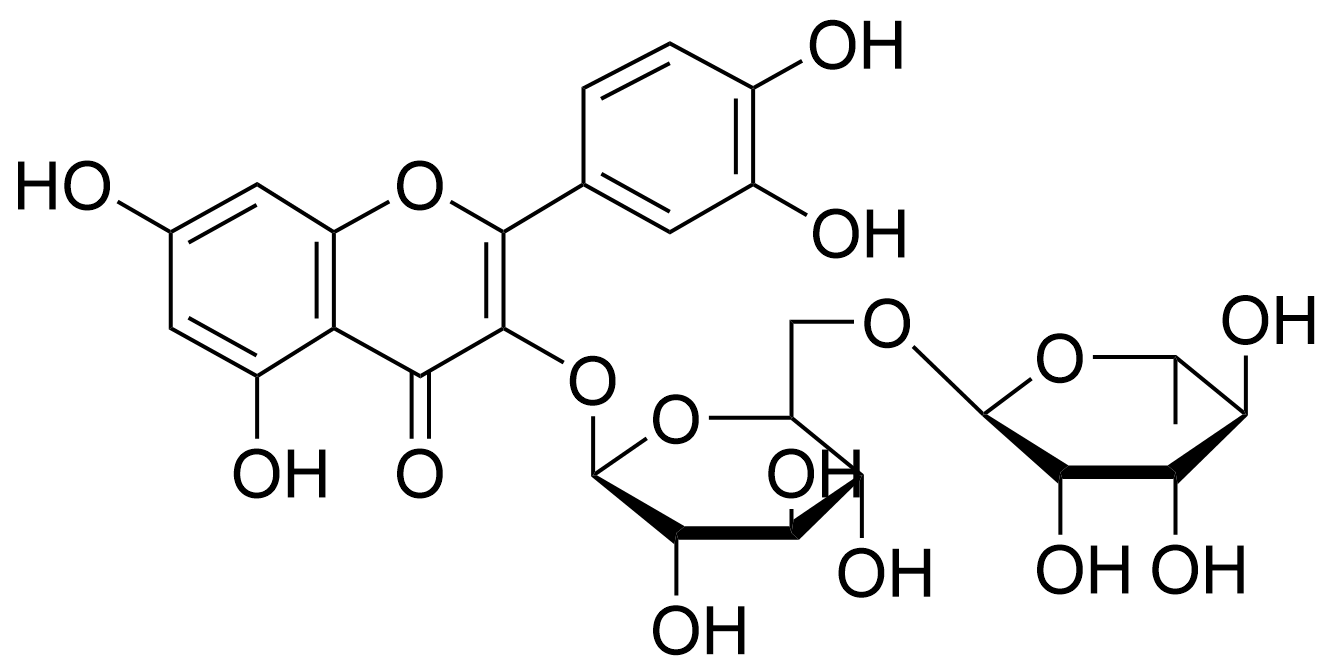 |
| 28 | Sphingosine-1-phosphate | 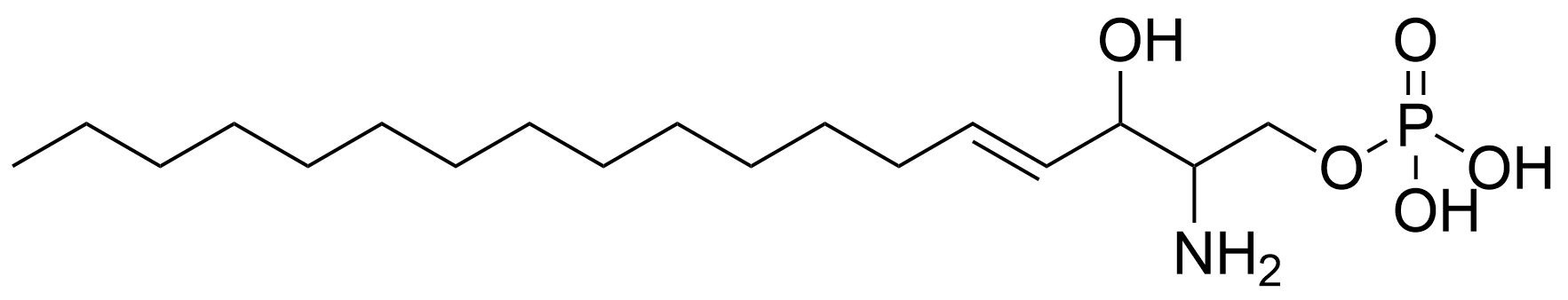 |
| 29 | Coenzyme Q10 | 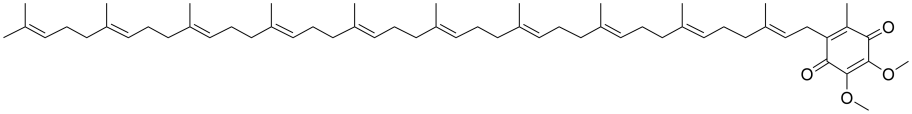 |
| 30 | Leonurine hydrochloride | 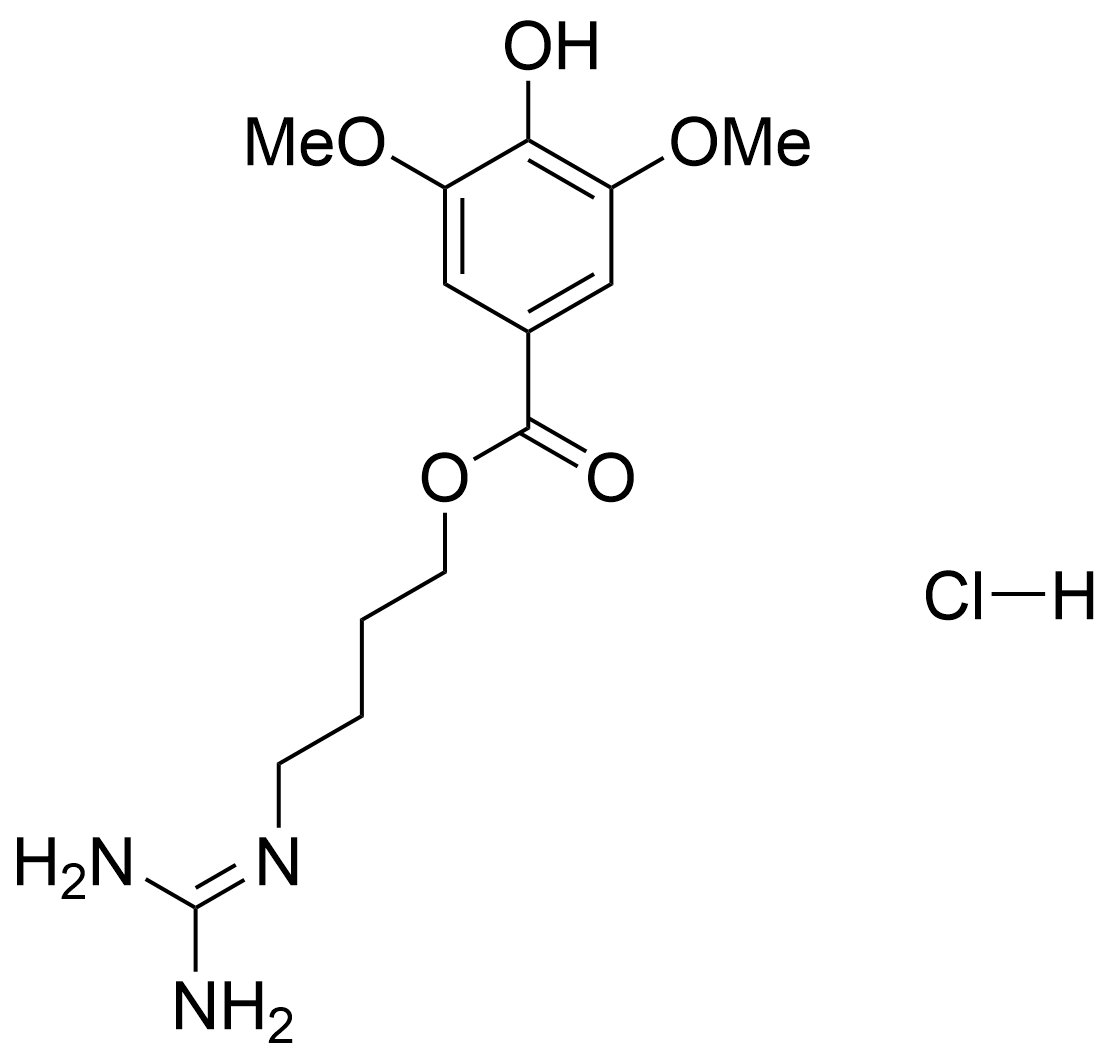 |
